# Anticancer Activity of Triazolo-Thiadiazole Derivatives and Inhibition of AKT1 and AKT2 Activation

**DOI:** 10.3390/pharmaceutics13040493

**Published:** 2021-04-05

**Authors:** Dimitrios T. Trafalis, Sofia Sagredou, Panayiotis Dalezis, Maria Voura, Stella Fountoulaki, Nikolaos Nikoleousakos, Konstantinos Almpanakis, Maria V. Deligiorgi, Vasiliki Sarli

**Affiliations:** 1 Laboratory of Pharmacology, Faculty of Medicine, National and Kapodistrian University of Athens, 115 27 Athens, Greece; ssagredou@med.uoa.gr (S.S.); pdalezis@med.uoa.gr (P.D.); nikosakos@hotmail.com (N.N.); mdeligiorgi@yahoo.com (M.V.D.); 2 Department of Chemistry, Aristotle University of Thessaloniki, University Campus, 541 24 Thessaloniki, Greece; vouram@gmail.com (M.V.); sfountou@chem.auth.gr (S.F.); konstalmp@yahoo.gr (K.A.)

**Keywords:** 1,2,4-triazolo[3,4-*b*]-1,2,4-thiadiazole, inhibitor of Akt phosphorylation, anticancer, MTT assay, HT-29 human colon tumor xenograft, ATP binding site

## Abstract

The fusion of 1,2,4-triazole and 1,3,4-thiadiazole rings results in a class of heterocycles compounds with an extensive range of pharmacological properties. A series of 1,2,4-triazolo[3,4-*b*]-1,2,4-thiadiazoles was synthesized and tested for its enzyme inhibition potential and anticancer activity. The results show that 1,2,4-triazolo[3,4-*b*]-1,2,4-thiadiazoles display potent anticancer properties in vitro against a panel of cancer cells and in vivo efficacy in HT-29 human colon tumor xenograft in CB17 severe combined immunodeficient (SCID) mice. Preliminary mechanistic studies revealed that KA25 and KA39 exhibit time- and concentration-dependent inhibition of Akt Ser-473 phosphorylation. Molecular modeling experiments indicated that 1,2,4-triazolo[3,4-*b*]-1,2,4-thiadiazoles bind well to the ATP binding site in Akt1 and Akt2. The low acute toxicity combined with in vitro and in vivo anticancer activity render triazolo[3,4-*b*]thiadiazoles KA25, KA26, and KA39 promising cancer therapeutic agents.

## 1. Introduction

Akt, termed protein kinase B (PKB), is a serine/threonine kinase composed of three isoforms: Akt1, Akt2, and Akt3. All three isoforms share very similar amino acid sequences, with their expression level differentiated; Akt1 and Akt2 are abundantly expressed, while Akt3 is particularly detected in the brain, heart, and kidneys [1,2]. All Akt isoforms display the same basic structure comprised of three regions: (1) an amino terminal pleckstrin homology (PH) domain that interacts with membrane phospholipids such as phosphatidylinositol-3,4,5-triphosphate (PIP3) and phosphatidylinositol 4,5-bisphosphate (PIP2); (2) a central kinase domain that contains the threonine regulatory residue, Thr308, in which phosphorylation activates Akt; and (3) a carboxyl-terminal regulatory domain that consists of a hydrophobic region of 40 amino acids including the serine regulatory residue (Ser473) [3]. The structural elements, PH domain, and the regulatory residues Thr308 and Ser473 play critical roles in the activation of Akt. Two events are required in Akt’s activation: (a) PH-domain-dependent translocation to the plasma membrane and (b) phosphorylation at the Thr308 and Ser473 residues. The first step includes the interaction of the PH domain with PIP3, which is followed by the translocation of Akt to the plasma membrane. Afterwards, Akt adopts a new conformation such that the Thr308 residue would be phosphorylated by phosphoinositide-dependent kinase-1 (PDK1). This signaling event leads to phosphorylated Ser473 via the mechanistic targeting of rapamycin (mTOR)C2 [4,5,6].

Phosphatidylinositol 3-kinase (PI3K), an upstream signaling molecule, along with Akt constitute the PI3K/Akt signaling transduction pathway through which cellular survival and growth are induced in response to extracellular signal [7]. Among all protein components of the PI3K/Akt pathway, the inhibition of Akt has been widely explored due to its association with tumor progression and aggressiveness [8]. Significant alterations have been demonstrated in the expression levels of Akt isoforms in certain malignancies, for instance, Akt1 is particularly elevated in breast, prostate, and gastric tumors whilst Akt2 is overexpressed in prostate, ovarian, breast, pancreatic, and colorectal cancers [9,10,11]. Thus far, Akt inhibitors are divided in four basic categories: (1) ATP-competitive inhibitors, (2) allosteric inhibitors, (3) lipid-based inhibitors, and 4) PH domain inhibitors [12]. The first category of ATP-competitive inhibitors (CCT128930 as an Akt2 inhibitor and BAY-1125976 as an Akt1/2 inhibitor), including pan-Akt kinase inhibitors (afuresertib, GSK690693, AZD5363, GDC-0068, and AT7867 as inhibitors of all Akt isoforms), targets the kinase domain and precisely binds to the ATP-binding pocket [13]. The high degree of homology of the ATP-binding site among different serine/threonine kinases as well as the extensive conservation of this domain within the AGC kinase family (protein kinase A, G, and C families) contributes to a low specificity of ATP-competitive inhibitors. The development of such molecules can be further obstructed due to the lack of strong efficacy against tumors in vivo, with toxicities to normal tissues being recorded at the same time [14,15]. Regarding allosteric inhibitors, for example, the MK-2206 compound, are associated with the Akt kinase domain. Compared with ATP-competitive inhibitors that show efficacy only against cancer cell lines with Akt mutations, allosteric inhibitors display broader anticancer activity as compounds of this type are potent against cancer cell lines with PI3KCA mutations or loss of phosphatase and tensin homologue (PTEN) activity [16]. The third category of lipid-based inhibitors (PX-866 and perifosine) blocks the interaction of Akt with PIP3 since this type of molecule inhibits PI3K and therefore prevents the production of PIP3 from PIP2. Finally, PH domain inhibitors (triciribine and PX-316) inactivate Akt via interactions with the PH-domain, interrupting membrane translocation, which is required for activating Akt [17].

**Figure 1:**
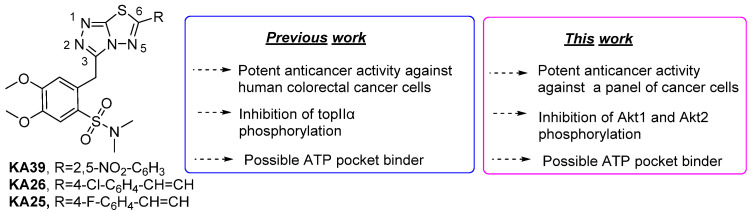
Structures of active triazolo[3,4-*b*][1,3,4]thiadiazoles and overview of their activity.

The fusion of 1,2,4-triazole and 1,3,4-thiadiazole rings results in a class of heterocyclic compounds with an extensive range of pharmacological properties including antifungal, antibacterial, antiviral, anti-inflammatory, analgesic, and anthelmintic properties [18]. Earlier studies indicated that previous 3,6-disubstituted 1,2,4-[3,4-*b*]thiadiazoles induced potent anti-inflammatory activity along with a minimal ulcerogenic effect and lipid peroxidation compared to ibuprofen and flurbiprofen. Some of these compounds demonstrated moderate to weak antibacterial activity against *Staphylococcus aureus* and *Escherichia coli* while their antifungal activity against *Candida albicans* was quite weak [19,20,21]. Furthermore, 1,2,4-triazolo[3,4-*b*][1,3,4]thiadiazole derivatives containing 3-methyl or benzyl moiety showed moderate anti-HIV-1 activity at subcytotoxic concentrations [22]. In previous work, we have shown that triazolo[3,4-*b*]thiadiazole derivatives show potent in vitro antiproliferative activities [23]. Our studies resulted in the identification of three bioactive compounds (KA39, KA25, and KA26) against three human colorectal cancer cell lines (Figure 1). Among them, KA39 was the most potent anticancer agent and inhibitor of topIIα phosphorylation at Ser-1106 as well. Additional molecular docking studies revealed that KA39 can occupy the same binding site as etoposide and can interact with the ATPase domain of topIIα, but the exact mechanism of inhibition of topIIα phosphorylation by KA39 is still unclear. As part of our ongoing work on the assessment of the biological activity of triazolo[3,4-*b*][1,3,4]thiadiazoles, we report the synthesis, structural characterization, and evaluation of inhibitory effects of fifteen new derivatives. In particular, we examined the introduction of ethyl or propyl substituents on sulfonamide combined with different substituents on C-6 of the triazolo[3,4-*b*][1,3,4]thiadiazole template. In the current work, the anticancer activities of all derivatives were primarily assessed in vitro while the most active molecules KA39, KA25, and KA26, according to in vitro screening, were tested in vivo. Further studies were carried out to investigate whether the most potent antitumor compound blocks the phosphorylation of Αkt1 and Αkt2 kinases. The molecular modeling studies indicated that 1,2,4-triazolo[3,4-*b*]-1,2,4-thiadiazoles bind well to the ATP binding site in Akt1 and Akt2.

## 2. Materials and Methods

### 2.1. Synthesis of Triazolo-Thiadiazole Derivatives

All reactions were carried out under an atmosphere of argon unless otherwise specified. Commercial reagents of high purity were purchased and used without further purification, unless otherwise noted. Reactions were monitored by thin-layer chromatography (TLC) and by using UV light as a visualizing agent and ethanolic *p*-anisaldehyde solution or using aqueous ceric sulfate/phosphomolybdic acid and heat as developing agents. The ^1^H and ^13^C NMR spectra were recorded at 500 and 126 MHz, and tetramethylsilane was used as an internal standard. Chemical shifts are indicated in δ values (ppm) from internal reference peaks (Tetramethylsilane TMS ^1^H 0.00; CDCl_3_^1^H 7.26, ^13^C 77.00; DMSO-*d_6_* ^1^H 2.50, and ^13^C 39.51). Melting points (m.p.) are uncorrected. The LC-MS spectra were recorded on a LC20AD Shimadzu connected to Shimadzu LCMS-2010EV equipped with C18 analytical column (SUPELCO Discovery (C18, 25 cm × 4.6 mm, 5 μm)). Compound 2, KA25, KA26, and KA39 were synthesized according to a previously reported procedure [23].

Methyl 2-(3,4-dimethoxyphenyl)acetate, S1: 2-(3,4-dimethoxyphenyl)acetic acid (1 g, 5.1 mmol) was dissolved in 24 mL dry dichloromethane (DCM), a catalytic amount of dry dimethylformamide (DMF) was added, and the mixture was cooled at 0 °C. Then, 1.32 mL (15.31 mmol) of oxalyl chloride was added dropwise and the reaction was left stirring at room temperature for 0.5 h. The addition of MeOH (20 mL) in portions at 0 °C was followed, and the reaction was stirred at room temperature for 10 min. The reaction was quenched with 20 mL of H_2_O, and the mixture was extracted with dichloromethane (3 × 20 mL). The organic extracts were dried over Na_2_SO_4_, filtered, and concentrated under reduced pressure. The residue was purified by silica gel column chromatography (eluent; hexane/ethyl acetate, 4/1) to afford S1 as a yellow oil in 99% yield. The spectral data were in accordance with those reported in the literature [24]. S1: ^1^H NMR (500 MHz, CDCl_3_) δ 6.81 (s, 3H), 3.88 (s, 3H), 3.86 (s, 3H), 3.69 (s, 3H), 3.57 (s, 2H).

Methyl 2-(2-(chlorosulfonyl)-4,5-dimethoxyphenyl)acetate, 2: to a solution of S1 (3.7 g, 17.64 mmol) in 35 mL CHCl_3_, 5.28 mL (79.4 mmol) of chlorosulfonic acid was added dropwise at 0 °C, and the reaction was stirred at room temperature for 1 h. The reaction was quenched with H_2_O (35 mL), and the mixture was extracted with dichloromethane (×3). The organic layers were combined, dried over Na_2_SO_4_, filtered, and concentrated under reduced pressure. The residue was purified by silica gel column chromatography (eluent; hexane/ethyl acetate = 2/1) to yield compound 2 as a white solid (88%). The spectral data were in accordance with those reported in the literature [23]. 2: ^1^H NMR (500 MHz, CDCl_3_) δ 7.52 (s, 1H), 6.88 (s, 1H), 4.11 (s, 2H), 3.97 (s, 3H), 3.95 (s, 3H), 3.73 (s, 3H); ^13^C NMR (126 MHz, CDCl_3_) δ 170.5, 154.1, 148.0, 134.6, 127.8, 115.3, 111.3, 56.4, 56.4, 52.4, 38.0.

Methyl 2-(2-(*N*,*N*-diethylsulfamoyl)-4,5-dimethoxyphenyl)acetate, 3a: to the solution of **2** (450 mg, 1.46 mmol) in 1.8 mL tetrahydrofuran (THF), 0.3 mL diethylamine (2.92 mmol) was added at 0 °C. The reaction mixture was stirred for 1 h at room temperature. The mixture was concentrated under reduced pressure to obtain 3a as a yellow solid (99% yield). 3a: m.p. = 108–110 °C; ^1^H NMR (500 MHz, CDCl_3_) δ 7.45 (s, 1H), 6.82 (s, 1H), 3.99 (s, 2H), 3.93 (s, 3H), 3.92 (s, 3H), 3.71 (s, 3H), 3.25 (q, *J* = 7.1 Hz, 4H), 1.12 (t, *J* = 7.1 Hz, 6H); ^13^C NMR (126 MHz, CDCl_3_) δ 171.6, 151.9, 147.5, 130.0, 127.0, 115.2, 112.9, 56.3, 56.1, 52.0, 40.5, 37.6, 13.4; ESI–MS *m/z* for C_15_H_23_NNaO_6_S [M + Na]^+^ calcd 368.11, found 367.95.

Methyl 2-(2-(*N*,*N*-diisopropylsulfamoyl)-4,5-dimethoxyphenyl)acetate, 3b: 450 mg (1.46 mmol) of 2 was dissolved in 1.8 mL of THF, and 0.41 mL of diisopropylamine (2.92 mmol) was added at 0 °C. The reaction was stirred for 1 h at room temperature. THF and amine excess were concentrated under reduced pressure to afford 3b as a yellow solid in 99% yield. 3b: m.p. = 105–107 °C; ^1^H NMR (500 MHz, CDCl_3_) δ 7.44 (s, 1H), 6.76 (s, 1H), 4.08 (s, 2H), 3.92 (s, 3H), 3.90 (s, 3H), 3.70 (s, 3H), 3.58 (m, 2H), 1.31 (d, *J* = 6.8 Hz, 12H); ^13^C NMR (126 MHz, CDCl_3_) δ 171.7, 151.7, 147.5, 131.1, 127.3, 114.8, 112.7, 56.1, 56.1, 52.0, 48.6, 37.8, 21.8; ESI–MS *m/z* for C_17_H_27_NNaO_6_S [M + Na]^+^ calcd 396.15, found 395.95.

2-(2-hydrazinyl-2-oxoethyl)-*N*,*N*-diethyl-4,5-dimethoxybenzenesulfonamide, 4a: a mixture of compound 3a (185 mg, 0.513 mmol) in hydrazine hydrate 80% (0.22mL, 4.56 mmol) was refluxed for 0.5 h. The mixture was extracted with dichloromethane (3 × 8 mL), and the combined organic extracts were dried over Na_2_SO_4_, filtered, and concentrated under reduced pressure to afford 161 mg of compound 4a (87% yield, white solid). 4a: m.p. = 121–123 °C; ^1^H NMR (500 MHz, CDCl_3_) δ 7.82 (s, 1H), 7.23 (s, 1H), 6.89 (s, 1H), 3.79 (s, 3H), 3.76 (s, 3H), 3.66 (s, 2H), 3.14 (q, *J* = 7.1 Hz, 4H), 0.98 (t, *J* =7.1 Hz, 6H); ^13^C NMR (126 MHz, CDCl_3_) δ 170.3, 151.8, 147.1, 129.1, 127.4, 114.2, 112.0, 55.9, 40.4, 37.7, 13.2 (1 carbon missing due to overlapping); ESI–MS *m/z* for C_14_H_23_N_3_NaO_5_S [M + Na]^+^ calcd 368.13, found 367.95.

2-(2-hydrazinyl-2-oxoethyl)-*N*,*N*-diisopropyl-4,5-dimethoxybenzenesulfonamide, 4b: a mixture of compound 3b (250 mg, 0.67 mmol) in hydrazine hydrate 80% (0.28 mL, 5.66 mmol) was refluxed for 0.5 h. The mixture was extracted with dichloromethane (3 × 10 mL), and the combined organic extracts were dried over Na_2_SO_4_, filtered, and concentrated under reduced pressure to afford 230 mg of compound 4b in 92% yield as a yellow solid. 4b: m.p. = 121–123 °C; ^1^H NMR (500 MHz, CDCl_3_) 7.90 (s,1H), 7.43 (s, 1H), 7.01 (s, 1H), 3.93 (s, 3H), 3.90 (s,3H), 3.89 (s, 2H), 1.32 (d, *J* = 6.8 Hz, 12H); ^13^C NMR (126 MHz, CDCl_3_) δ 171.0, 152.2, 147.5, 130.1, 128.0, 114.1, 112.7, 56.3, 56.2, 49.0, 37.8, 21.9; ESI–MS *m/z* for C_16_H_27_N_3_NaO_5_S [M + Na]^+^ calcd 396.16, found 396.00.

2-((4-amino-5-mercapto-4*H*-1,2,4-triazol-3-yl)methyl)-*N*,*N*-diethyl-4,5-dimethoxybenzene sulfonamide, 5a: to a flask containing 400 mg (1.16 mmol) of 4a, abs EtOH (16 mL) and KOH (97.6 mg, 1.74 mmol) were successively added. Then, 105 μL (1.74 mmol) of CS_2_ was added dropwise at 0 °C and the reaction mixture was allowed to warm to room temperature and was stirred for 24 h. Subsequently, 16 mL of diethyl ether was added, and the solid product was filtered and washed with diethyl ether. The product was dried and used in the next reaction without further purification. To a sealed vial, the previous salt and hydrazine hydrate 80% (4.49 mL, 92.41 mmol) was added and the mixture was refluxed for 2 h. Acidification with 10% aq. HCl was followed, and the pH was adjusted to 7. The product was formed, filtered through a Gooch funnel, washed with cold water, and dried to afford 326 mg of compound 5a in 70% yield as a white solid. 5a: m.p. = 187–189 °C; ^1^H NMR (500 MHz, DMSO-*d_6_*) δ 13.42 (s, 1H), 7.30 (s, 1H), 6.97 (s, 1H), 5.58 (s, 2H), 4.28 (s, 2H), 3.82 (s, 3H), 3.78 (s, 3H), 3.16 (q, *J* = 7.0, 4H), 0.99 (t, *J* =7.0 Hz, 6H); ^13^C NMR (126 MHz, DMSO-*d_6_*) δ 165.9, 151.5, 151.3, 147.0, 129.6, 127.1, 115.4, 112.3, 55.8, 40.5, 27.7, 13.5 (1 carbon missing due to overlapping); ESI–MS *m/z* for C_15_H_23_N_5_NaO_4_S_2_ [M + Na]^+^ calcd 424.11, found 423.90.

2-((4-amino-5-mercapto-4*H*-1,2,4-triazol-3-yl)methyl)-*N*,*N*-diisopropyl-4,5-dimethoxy benzene sulfonamide, 5b: to a flask containing 300 mg (0.803 mmol) of 4b, abs EtOH (12 mL) and KOH (67.3 mg, 1.2 mmol) were successively added. Then, 73 μL (1.2 mmol) of CS_2_ was added dropwise at 0 °C and the reaction mixture was allowed to warm to room temperature and was stirred for 24 h. Subsequently, 12 mL of diethyl ether was added, and the solid product was filtered and washed with diethyl ether. The product was dried and used in the next reaction without further purification. To a sealed vial, the previous salt and hydrazine hydrate 80% (3.11 mL, 63.97 mmol) was added and the mixture was refluxed for 2 h. Acidification with 10% aq. HCl was followed, and the pH was adjusted to 7. The product was formed, filtered through a Gooch funnel, washed with cold water, and dried to afford 258 mg of 5b as a white solid (75% yield). 5b: m.p. = 162–164 °C; ^1^H NMR (500 MHz, DMSO-*d_6_*) δ 13.42 (s, 1H), 7.31 (s, 1H), 6.88 (s, 1H), 5.55 (s, 2H), 4.38 (s, 2H), 3.80 (s, 3H), 3.77 (s, 3H), 3.65–3.53 (m, 2H), 1.21 (d, *J* = 6.7 Hz, 12H); ^13^C NMR (126 MHz, DMSO-*d_6_*) 165.9, 151.4, 151.4, 147.1, 130.6, 127.6, 114.8, 112.2, 55.8, 55.7, 48.0, 27.7, 21.6; ESI–MS *m/z* for C_17_H_27_N_5_NaO_4_S_2_ [M + Na]^+^ calcd 452.14, found 452.00.

General procedure for the preparation of 1,2,4-triazolo-[3,4-*b*]-1,3,4-thiadiazoles: the mixture of 0.268 mmol of 5a,b and 0.268 mmol of carboxylic acids in phosphorous oxychloride (0.4 mL) was stirred while heated under reflux for 2 h. The reaction mixture was cooled to room temperature and then poured into ice. Then, the pH was adjusted to 8 with aqueous K_2_CO_3_. The mixture was extracted with dichloromethane, and the organic extracts were dried over Na_2_SO_4_, filtered, and concentrated under reduced pressure. The residue was purified by silica gel column chromatography.

2-((6-(3-chlorophenyl)-[1,2,4]triazolo[3,4-*b*][1,3,4]thiadiazol-3-yl)methyl)-*N,N*-diethyl-4,5-dimethoxybenzenesulfonamide, 6a: beige solid, 55% yield, m.p. = 203–205 °C; ^1^H NMR (500 MHz, CDCl_3_) δ 7.95 (s, 1H), 7.91 (d, *J* = 7.7 Hz, 1H), 7.76 (d, *J* = 8.0 Hz, 1H), 7.66 (d, *J* = 8.0 Hz, 1H), 7.32 (s, 1H), 7.14 (s, 1H), 4.73 (s, 2H), 3.81 (s, 3H), 3.77 (s, 3H), 3.17 (d, *J* = 7.1 Hz, 4H), 0.99 (t, *J* = 7.1 Hz, 6H); ^13^C NMR (126 MHz, CDCl_3_) δ 164.6, 152.6, 151.6, 147.2, 146.2, 134.3, 132.6, 131.7, 131.0, 129.5, 127.3, 126.3, 125.9, 115.5, 112.3, 55.9, 40.5, 27.4, 13.4 (1 carbon is missing due to overlapping); ESI–MS *m/z* for C_22_H_24_ClN_5_NaO_4_S_2_ [M + Na]^+^ calcd 544.09, found 543.95.

2-((6-(4-chlorophenyl)-[1,2,4]triazolo[3,4-*b*][1,3,4]thiadiazol-3-yl)methyl)-*N,N*-diethyl-4,5-dimethoxybenzenesulfonamide, 6b: beige solid, 63% yield, m.p. = 128–130 °C; ^1^H NMR (500 MHz, CDCl_3_) δ 7.80 (d, *J* = 8.3 Hz, 2H), 7.49–7.51 (m, 3H), 6.93 (s, 1H), 4.87 (s, 2H), 3.92 (s, 3H), 3.84 (s, 3H), 3.27 (d, *J* = 7.1 Hz, 4H), 1.10 (t, *J* = 7.1 Hz, 6H); ^13^C NMR (126 MHz, CDCl_3_) δ 165.4, 152.2, 147.6, 139.1, 129.8, 128.3, 127.8, 127.4, 114.1, 113.0, 56.3, 56.2, 40.7, 27.7, 13.6, 13.4; (3 carbons missing due to overlapping) ESI–MS *m/z* for C_22_H_24_ClN_5_NaO_4_S_2_ [M + Na]^+^ calcd 544.09, found 543.95.

*N,N*-diethyl-2-((6-(4-fluorophenyl)-[1,2,4]triazolo[3,4-*b*][1,3,4]thiadiazol-3-yl)methyl)-4,5-dimethoxybenzenesulfonamide, 6c: yellow solid, 58% yield, m.p. = 169–171 °C; ^1^H NMR (500 MHz, DMSO-*d_6_*) δ 8.01 (m, 2H), 7.48 (t, *J* = 8.7 Hz, 2H), 7.33 (s, 1H), 7.15 (s, 1H), 4.74 (s, 2H), 3.83 (s, 3H), 3.78 (s, 3H), 3.18 (d, *J* = 7.1 Hz, 4H), 0.98 (t, *J* = 7.1 Hz, 6H); ^13^C NMR (126 MHz, DMSO-*d_6_*) δ 165.0, 164.6, 152.6, 151.6, 147.2, 146.2, 129.7, 129.4, 127.4, 125.7, 117.0, 115.5, 112.3, 55.9, 55.9, 40.5, 27.4, 13.4; ESI–MS *m/z* C_22_H_24_FN_5_NaO_4_S_2_ [M + Na]^+^ calcd 528.12, found 527.85.

*N,N*-diethyl-4,5-dimethoxy-2-((6-(4-nitrophenyl)-[1,2,4]triazolo[3,4-*b*][1,3,4]thiadiazol-3-yl) methyl)benzenesulfonamide, 6d: red solid, 58% yield, m.p. = 192–194 °C; ^1^H NMR (500 MHz, CDCl_3_) δ 8.44 (d, *J* = 8.7 Hz, 2H), 8.22 (d, *J* = 8.7 Hz, 2H), 7.34 (s, 1H), 7.16 (s, 1H), 4.77 (s, 2H), 3.84 (s, 3H), 3.79 (s, 3H), 3.19 (q, *J* = 7.1 Hz, 4H), 0.99 (t, *J* = 7.1 Hz, 6H); ^13^C NMR (126 MHz, CDCl_3_) δ 164.2, 151.6, 149.6, 147.2, 134.6, 130.7, 129.5, 128.5, 127.3, 124.8, 123.7, 115.5, 112.3, 55.9, 40.5, 27.4, 13.5 (1 carbon is missing due to overlapping); ESI–MS *m/z* for C_22_H_24_N_6_NaO_6_S_2_ [M + Na]^+^ calcd 555.11, found 554.90.

2-((6-(2,5-dinitrophenyl)-[1,2,4]triazolo[3,4-*b*][1,3,4]thiadiazol-3-yl)methyl)-*N,N*-diethyl-4,5-dimethoxybenzenesulfonamide, 6e: orange solid, 32% yield, m.p. = 201–203 °C; ^1^H NMR (500 MHz, DMSO-*d_6_*) δ 8.73 (s, 1H), 8.70 (d, *J* = 8.8 Hz, 1H), 8.50 (d, *J* = 8.8 Hz, 1H), 7.33 (s, 1H), 7.08 (s, 1H), 4.70 (s, 2H), 3.83 (s, 3H), 3.78 (s, 3H), 3.12 (q, *J* = 7.0, 4H), 0.94 (t, *J* = 7.0 Hz, 6H); ^13^C NMR (126 MHz, DMSO-*d_6_*) δ 161.2, 157.6, 151.7, 150.6, 149.4, 147.2, 129.4, 128.2, 127.7, 127.1, 127.0, 124.4, 115.3, 112.5, 55.9, 55.8, 40.3, 27.3, 13.3 (1 carbon is missing due to overlapping); ESI–MS *m/z* for C_22_H_23_N_7_NaO_8_S_2_ [M + Na]^+^ calcd 600.09, found 599.90.

2-((6-(3-chlorophenyl)-[1,2,4]triazolo[3,4-*b*][1,3,4]thiadiazol-3-yl)methyl)-*N,N*-diisopropyl-4,5-dimethoxybenzenesulfonamide, 7a: yellow solid, 53% yield, m.p. = 160–162 °C; ^1^H NMR (500 MHz, DMSO-*d_6_*) δ 7.96 (s, 1H), 7.90 (d, *J* = 7.6 Hz, 1H), 7.76 (d, *J* = 7.9 Hz, 1H), 7.65 (t, *J* = 7.9 Hz, 1H), 7.37 (s, 1H), 7.09 (s, 1H), 4.85 (s, 2H), 3.82 (s, 3H), 3.77 (s, 3H), 3.59–3.65 (m, 2H), 1.23 (d, *J* = 6.7 Hz, 12H); ^13^C NMR (126 MHz, DMSO-*d_6_*) δ 164.6, 151.9, 151.6, 147.2, 146.3, 134.3, 132.5, 131.6, 130.9, 130.4, 127.6, 126.3, 125.9, 115.0, 112.4, 55.8, 55.8, 48.1. 27.3, 21.5; ESI–MS *m/z* for C_24_H_29_ClN_5_O_4_S_2_ [M + H]^+^ calcd 550.13, found 549.85.

2-((6-(4-fluorophenyl)-[1,2,4]triazolo[3,4-*b*][1,3,4]thiadiazol-3-yl)methyl)-*N,N*-diisopropyl-4,5-dimethoxybenzenesulfonamide, 7b: beige solid, 65% yield, m.p. = 177–179 °C; ^1^H NMR (500 MHz, DMSO-*d_6_*) δ 8.00 (dd, *J* = 8.8, 5.2 Hz, 2H), 7.47 (t, *J* = 8.8 Hz, 2H), 7.37 (s, 1H), 7.09 (s, 1H), 4.84 (s, 2H), 3.82 (s, 3H), 3.77 (s, 3H), 3.36–3.64 (m, 2H), 1.22 (d, *J* = 6.8, 12H); ^13^C NMR (126 MHz, DMSO-*d_6_*) δ 165.0, 164.5, 152.6, 151.6, 147.2, 146.3, 130.4, 129.7, 127.6, 125.7, 117.0, 115.0, 112.5, 55.8, 55.8, 48.1, 27.3, 21.5; ESI–MS *m/z* for C_24_H_29_FN_5_NaO_4_S_2_ [M + Na]^+^ calcd 556.15, found 555.95.

*N,N*-diisopropyl-4,5-dimethoxy-2-((6-(4-nitrophenyl)-[1,2,4]triazolo[3,4-*b*][1,3,4]thiadiazol-3-yl)methyl)benzenesulfonamide, 7c: beige solid, 54% yield, m.p. = 212–214 °C; ^1^H NMR (500 MHz, CDCl_3_) δ 8.36 (d, *J* = 8.8, 5.2 Hz, 2H), 8.07 (d, *J* = 8.8 Hz, 2H), 7.49 (s, 1H), 6.89 (s, 1H), 5.00 (s, 2H), 3.90 (s, 3H), 3.84 (s, 3H), 3.59–3.66 (m, 2H), 1.33 (d, *J* = 6.8Hz, 12H); ^13^C NMR (126 MHz, CDCl_3_) δ 163.8, 153.3, 152.2, 150.1, 147.9, 147.4, 135.1, 131.3, 128.3, 127.5, 124.7, 113.9, 113.0, 56.3, 56.3, 48.9, 27.9, 22.1; ESI–MS *m/z* C_24_H_28_N_6_NaO_6_S_2_ [M + Na]^+^ calcd 583.14, found 582.95.

2-((6-(2,5-dinitrophenyl)-[1,2,4]triazolo[3,4-*b*][1,3,4]thiadiazol-3-yl)methyl)-*N,N*-diisopropyl-4,5-dimethoxybenzenesulfonamide, 7d: yellow solid, 35% yield, m.p. = 180–182 °C; ^1^H NMR (500 MHz, DMSO-*d_6_*) δ 8.71 (dd, *J* = 15.3, 5.5 Hz, 2H), 8.50 (d, *J* = 8.8 Hz, 1H), 7.35 (s, 1H), 6.98 (s, 1H), 4.81 (s, 2H), 3.81 (s, 3H), 3.76 (s, 3H), 3.55 (2H, overlapping with H_2_O), 1.19 (d, *J* = 6.7 Hz, 12H); ^13^C NMR (126 MHz, DMSO-*d_6_*) δ 161.2, 151.6, 150.5, 149.3, 147.2, 146.1, 130.3, 128.2, 127.7, 127.4, 127.0, 124.5, 114.6, 112.5, 55.8, 55.7, 48.1, 27.2, 21.5; (1 carbon is missing due to overlapping) ESI–MS *m/z* for C_24_H_27_N_7_KO_8_S_2_ [M + H]^+^ calcd 606.14, found 605.90.

(*E*)-2-((6-(4-chlorostyryl)-[1,2,4]triazolo[3,4-*b*][1,3,4]thiadiazol-3-yl)methyl)-*N,N*-diethyl-4,5-dimethoxybenzenesulfonamide, 9a: orange solid, 55% yield, m.p. = 196–198 °C; ^1^H NMR (500 MHz, DMSO-*d_6_*) δ 7.84 (d, *J* = 8.6 Hz, 2H), 7.64 (s, 2H), 7.53 (d, *J* = 8.6 Hz, 2H), 7.33 (s, 1H) 7.10 (s, 1H), 4.69 (s, 2H), 3.83 (s, 3H), 3.78 (s, 3H), 3.16 (q, *J* = 7.1 Hz, 4H), 0.98 (t, *J* = 7.1 Hz, 6H); ^13^C NMR (126 MHz, DMSO-*d_6_*) δ 165.7, 151.6, 147.1, 146.1, 139.4, 134.8, 133.4, 129.8, 129.4, 129.1, 127.3, 119.0, 115.4, 112.3, 55.9, 55.9, 40.5, 27.3, 13.4, 13.4 (1 carbon is missing due to overlapping); ESI–MS *m/z* for C_24_H_26_ClN_5_NaO_4_S_2_ [M + Na]^+^ calcd 570.10, found 569.95.

(*E*)-*N,N*-diethyl-2-((6-(4-fluorostyryl)-[1,2,4]triazolo[3,4-*b*][1,3,4]thiadiazol-3-yl)methyl)-4,5-dimethoxybenzenesulfonamide, 9b: yellow solid, 67% yield, m.p. = 118–120 °C, ^1^H NMR (500 MHz, DMSO-*d_6_*) δ 7.87 (dd, *J* = 8.6, 5.6 Hz, 2H), 7.65 (d, *J* = 16.4 Hz, 1H), 7.57 (d, *J* = 16.4 Hz, 1H), 7.31 (s, 1H) 7.30 (d, *J* = 8.6 Hz, 2H), 7.10 (s, 1H), 4.69 (s, 2H), 3.83 (s, 3H), 3.78 (s, 3H), 3.17 (q, *J* = 7.1 Hz, 4H), 0.98 (t, *J* =7.1 Hz, 6H); ^13^C NMR (126 MHz, DMSO-*d_6_*) δ 165.9, 163.2, 151.6, 147.2, 146.1, 139.6, 131.6, 130.5, 129.4, 127.4, 118.1, 116.1, 115.4, 112.3, 55.8, 40.5, 27.3, 13.5; (2 carbons are missing due to overlapping) ESI–MS *m/z* for C_24_H_27_FN_5_O_4_S_2_ [M + H]^+^ calcd 531.15, found 532.00.

(*E*)-*N,N*-diethyl-4,5-dimethoxy-2-((6-(3-nitrostyryl)-[1,2,4]triazolo[3,4-*b*][1,3,4]thiadiazol-3-yl)methyl)benzenesulfonamide, 9c: brown solid, 37% yield, m.p. = 110–112 °C; ^1^H NMR (500 MHz, CDCl_3_) δ 8.09 (d, *J* = 8.2 Hz, 1H), 7.85 (d, *J* = 16.2 Hz, 1H), 7.70 (d, *J* = 4.1 Hz, 2H), 7.58 (dd, *J* = 8.4, 4.2 Hz, 1H), 7.47 (s, 1H), 7.11 (d, *J* = 16.2 Hz, 1H), 6.88 (s, 1H), 4.81 (s, 2H), 3.90 (s, 3H), 3.82 (s, 3H), 3.25 (q, *J* = 7.1 Hz, 4H), 1.08 (t, *J* = 7.1 Hz, 6H); ^13^C NMR (126 MHz, CDCl_3_) δ 164.6, 152.6, 152.1, 147.7, 147.5, 146.5, 136.0, 133.9, 130.6, 130.0, 129.6, 128.8, 127.4, 125.3, 122.6, 113.9, 113.0, 56.2, 56.1, 40.6, 27.5, 13.4; ESI–MS *m/z* for C_24_H_26_N_6_NaO_6_S_2_ [M + Na]^+^ calcd 581.13, found 581.10.

(*E*)-*N,N*-diethyl-4,5-dimethoxy-2-((6-(4-nitrostyryl)-[1,2,4]triazolo[3,4-*b*][1,3,4]thiadiazol-3-yl)methyl)benzenesulfonamide, 9d: brown solid, 26% yield, m.p. = 126–128 °C; ^1^H NMR (500 MHz, CDCl_3_) δ 8.28 (d, *J* = 8.4 Hz, 2H), 7.71 (d, *J* = 8.4 Hz, 2H), 7.48 (s, 1H), 7.33 (d, *J* = 12.8 Hz, 2H), 6.89 (s, 1H), 4.83 (s, 2H), 3.91 (s, 3H), 3.83 (s, 3H), 3.26 (q, *J* = 7.1 Hz, 4H), 1.11 (t, *J* = 7.1 Hz, 6H); ^13^C NMR (126 MHz, CDCl_3_) δ 164.4, 152.5, 152.1, 148.5, 147.6, 146.7, 139.9, 137.6, 129.7, 128.3, 127.3, 124.4, 122.1, 114.0, 113.0, 56.3, 56.2, 40.7, 27.6, 13.5. ESI–MS *m/z* for C_24_H_26_N_6_NaO_6_S_2_ [M + Na]^+^ calcd 581.13, found 580.90.

(*E*)-2-((6-(4-chlorostyryl)-[1,2,4]triazolo[3,4-*b*][1,3,4]thiadiazol-3-yl)methyl)-*N,N*-diisopro-pyl-4,5-dimethoxybenzenesulfonamide, 10a: white solid, 54% yield, m.p. = 135–137 °C; ^1^H NMR (500 MHz, DMSO-*d_6_*) δ 7.83 (d, *J* = 8.6 Hz, 2H), 7.63 (d, *J* = 2.5 Hz, 2H), 7.52 (d, *J* = 8.5 Hz, 2H), 7.35 (s, 1H), 7.03 (s, 1H), 4.78 (s, 2H), 3.82 (s, 3H), 3.77 (s, 3H), 3.63–3.56 (m, 2H), 1.21 (d, *J* = 6.8 Hz, 12H); ^13^C NMR (126 MHz, DMSO-*d_6_*) δ 165.7, 151.5, 147.2, 139.3, 134.8, 133.4, 130.4, 129.8, 129.0, 127.7, 119.0, 114.8, 112.4, 55.8, 55.8, 48.1, 27.3, 21.5; (2 carbons are missing due to overlapping) ESI–MS *m/z* for C_26_H_30_ClN_5_O_4_S_2_ [M + H]^+^ calcd 576.15, found 575.95.

(*E*)-2-((6-(4-fluorostyryl)-[1,2,4]triazolo[3,4-*b*][1,3,4]thiadiazol-3-yl)methyl)-*N,N*-diisopro-pyl-4,5-dimethoxybenzenesulfonamide, 10b: yellow solid, 66% yield, m.p. = 127–129 °C; ^1^H NMR (500 MHz, DMSO-*d_6_*) δ 7.88 (dd, *J* = 8.6, 5.6 Hz, 2H), 7.65(d, *J* = 16.4 Hz, 1H), 7.56 (d, *J* = 16.4 Hz, 1H), 7.36 (s, 1H), 7.31 (d, *J* = 8.6, 2H), 7.08 (s, 1H), 4.78 (s, 2H), 3.82 (s, 3H), 3.77 (s, 3H), 3.57–3.62 (m, 2H), 1.22 (d, *J* = 6.7 Hz, 12H); ^13^C NMR (126 MHz, DMSO-*d_6_*) δ 165.8, 163.2, 151.5, 147.2, 146.2, 139.5, 131.1, 130.5, 130.4, 127.7, 118.1, 116.1, 114.8, 112.4, 55.8, 55.8, 48.1, 27.3, 21.5; (1 carbon is missing due to overlapping) ESI–MS *m/z* for C_26_H_31_FN_5_O_4_S_2_ [M + H]^+^ calcd 560.18, found 560.05.

The NMR spectra of triazolo[3,4-*b*]thiadiazoles derivatives are presented in the Supplementary Materials.

### 2.2. Cell Lines and Culture Conditions

The in vitro antiproliferative activities of the novel triazolo[3,4-*b*]thiadiazole derivatives were estimated in ten well-established human cancer cell lines including three ovarian (SKOV-3, UWB 1.289, and UWB 1.289+BRCA1), five colorectal adenocarcinoma (DLD-1, HT-29, LS174T, LoVo, and SW403), and two prostate cancer cell lines (PC-3 and DU-145). All cancer cell lines were obtained from the American Type Culture Collection (ATCC) and cultivated in different culture media according to the instructions. The required growth mediums were supplemented with 10% fetal bovine serum and 1% streptomycin/penicillin. All cancer cell lines were cultured as monolayers and maintained at 37 °C, 5% CO_2_ in humidified air.

### 2.3. Drug Preparation for In Vitro Studies

Stock solutions of all tested compounds were prepared by dissolving the required amount of substances in the DMSO solvent to reach the desired concentration of 15 mM. The final volume of DMSO did not exceed 1% in the culture medium so that no cytotoxic effects could be revealed.

### 2.4. In Vitro Antiproliferative Assay

To evaluate the in vitro antiproliferative activity of the triazolo[3,4-*b*]thiadiazole derivatives, 3-(4,5-dimethylthiazol-2-yl)-2,5-diphenyltetrazolium bromide (MTT) assay was assessed. According to the protocol, cells were firstly seeded into a 96-well plate at a density of 8 × 10^3^ cells and grown for 24 h at 37 °C, 5% CO_2_ in humidified air. Subsequently, cells were treated with the tested compounds in a specific range of drug concentrations (1–100 μΜ). Following 48 h of drug exposure, the culture media were discarded and replaced with 100 μL of fresh medium. Afterwards, 50 μL of dissolved MTT in phosphate-buffered saline (PBS) solution (5 mg/mL) were added in each well and cells were incubated for 3 h at 37 °C, 5% CO_2_ in humidified air. Incubation time was followed by MTT discard and the addition of 100 μL of DMSO solvent so that the formazan crystals could be dissolved. The absorbance of the converted dye was immediately measured at a wavelength of 540 nm on an ELISA reader (VersaMax Microplate Reader, Orleans, CA, USA) [25].

The in vitro anticancer effect of a tested compound on cell viability was determined by three dose–response parameters termed GI_50_, TGI, and IC_50_. More specifically, the GI_50_ value indicates the required concentration of the compound that impels 50% cell growth inhibition, the TGI value corresponds to the concentration that induces 100% cell growth inhibition, and the IC_50_ value is the concentration that causes 50% cell death. The two parameters GI_50_ and TGI represent the cytostatic activity, whereas IC_50_ reflects the cytotoxic effect of a drug [26,27]. To estimate the three parameters, nine absorbance measurements were required including control 24 h (Ct24), control 72 h (Ct72), and the mean of cell survival in all seven drug concentrations (Tt72). The percentage growth inhibition was calculated according to National Cancer Institute’s (NCI) protocol: [(Tt72x) − (Ct24)/(Ct72) − (Ct24)] × 100 for concentrations for which Tt72x > Ct24 and [(Tt72x) − (Ct24)/Ct24] × 100 for concentrations for which Tt72x < Ct24. GI_50_ was calculated from [(Tt72x) − (Ct24)/(Ct72) − (Ct24)] × 100 = 50, TGI was calculated from [(Tt72x) − (Ct24)/(Ct72) − (Ct24)] × 100 = 0, and IC_50_ was calculated from [(Tt72x) − (Ct24)/Ct24] × 100 = 50. All the experiments were carried out in triplicate.

### 2.5. Animals

CB17 severe combined immunodeficient (SCID) male and female mice were used for toxicity and antitumor activity studies. Mice were obtained from National Center for Scientific Research (NCSR) Demokritos, Institute of Biology, Athens, Greece. CB17 SCID mice were fed water and an irradiated standard rodent diet ad libitum and housed under specific pathogen-free conditions. For ethical reasons, the weight and tumor volumes of the animals were measured twice a week during the experiment. In case of cachexia, suffering, or increase in the tumor volume up to 2000 mm^3^, the animals were sacrificed by cervical disruption. The protocol was approved by the ethical committee of the National and Kapodistrian University of Athens and was conducted according to the European Directive 86/609/EEC guidelines for the care and use of laboratory animals. The study received a permit from the Veterinary Directorate of the Prefecture of Athens (approval #: 2023/2017) according to the Greek legislation conforming to the 2010/53/ EU Council Directive.

### 2.6. In Vivo Acute Toxicity

For intraperitoneal (i.p.) treatment, stock solutions of the 3 tested compounds were prepared immediately before use. They were suspended in corn oil in the desired concentrations following initial dissolution in 10% dimethyl sulfoxide (DMSO). This concentration by itself produced no observable toxic effect. Briefly, the acute toxicity of the tested compounds was assessed for lethality in CB17 SCID mice and determined as previously described [28,29]. The three derivatives (KA25, KA26, and KA39) were tested at four dosages: 50, 100, 150, and 250 mg/kg. Each dosage comprised a group of ten mice, including males and females, that received single i.p. injections. Overall, forty mice were required to estimate the acute toxicity for each compound (10 mice/dosage). All mice were observed for 30 days. The therapeutic dose of the compounds is usually defined as LD10 (lethal dose for 10% of animals), while the short-term poisoning potential of a substance is termed LD50 (lethal dose for 50% of animals). Both doses were estimated graphically (30-day curves).

### 2.7. HT-29 Colorectal Adenocarcinoma

HT-29 (human colorectal adenocarcinoma) tumor volume was developed by subcutaneously implanting 2 × 10^6^ HT-29 cells/mouse in the right hind flank. In vivo antitumor activity was investigated in 22 mice, of which 7 mice served as the common control group and the 15 remaining mice were divided into three groups of 5 mice. Therefore, each derivative was administered in a group of five mice. The animals were randomized into test groups on day 28 when their tumor volumes were in the 150–200 mm^3^ range. After randomization, the mice were treated intraperitoneally (day 1) with the tested compounds (KA25, KA26, and KA39) as a single dose LD10. The control group received saline buffer (0.2 mL).

### 2.8. Estimation of Tumor Growth

The tumor volumes were measured using an analogue caliper twice a week starting on the first day of treatment. To determine the volume (mm^3^) of each tumor in an animal, the following formula was used: (D × d^2^/2), where D and d refer to the larger and smaller perpendicular dimensions collected at each measurement, respectively. The diameters were determined from two orthogonal measurements on each tumor. The mean tumor volume was determined by average all tumor volumes in each group [30]. The weekly mean tumor area change was determined and tumor inhibition (TI) was calculated by the formula: TI (%) = [1 − (TWT − TWZ)/[(TWC − TWZ)] × 100, where TWT is determined as the tumor area (mm^3^) in treated animals at the time of evaluation, TWZ is determined as the tumor area (mm^3^) at the time of initiation of treatment (zero time), and TWC is determined as the tumor volume (mm^3^) in untreated animals (controls) at the time of evaluation. The animals were sacrificed when the tumor volume reached 2000 mm^3^ (endpoint).

### 2.9. Detection of Akt1 and Akt2 Phosphorylation by Intracellular Flow Cytometry

To investigate whether KA25 and KA39 triazolo[3,4-*b*]thiadiazole derivatives inhibit the phosphorylation of Akt1 and Akt2 isoforms, three colorectal cancer cell lines including HT-29, LoVo, and SW403 were intracellularly stained upon treatment with the two referred compounds. Firstly, cells were seeded into a 6-well plate at a density of 7 × 10^5^ cells/well and maintained at 37 °C in a 5% humidified air. After 24 h of cell growth, the culture medium was discarded and replaced with 2 mL of fresh medium. Then, the cells were treated with KA25 and KA39 derivatives at GI50 and TGI concentrations (μΜ) for 3 and 24 h. The required GI50 and TGI concentrations (μΜ) of the KA25 and KA39 derivatives in HT-29 and LoVo cells have been determined in previous studies [23]. To estimate the TGI concentration (μΜ) of the KA25 compound in SW403 cells, further MTT assays were carried out as previously described. Following drug exposure at the mentioned treatment conditions, the cells were collected after being washed with ice-cold PBS (pH = 7.4) and detached enzymatically with standard trypsinization. Two centrifugations were followed at 1500 rpm for 5 min, including two washing steps with 2 mL of cell staining buffer (BioLegend, San Diego, CA, USA). Cell pellets were fixed with 500 μL of pre-warmed fixation buffer (BioLegend, San Diego, CA, USA) and incubated at 37 °C for 15 min. Afterwards, one centrifugation was carried out at 1000 rpm for 5 min, followed by supernatant discard and resuspension of cell pellet through vortex. Then, the cells were washed with 2 mL of cell staining buffer twice, including two centrifugations at 1000 rpm for 5 min at room temperature. After the cell pellet was resuspended with gentle pipetting, cells were permeabilized by adding 1000 μL of pre-chilled True-Phos Permeabilization buffer (BioLegend, San Diego, CA, USA) while vortexing. Following overnight incubation at −20 °C, the cells were centrifuged at 2000 rpm for 5 min and the supernatant was discarded. After the cell pellet was resuspended by vortexing, two centrifugations were performed at 2000 rpm for 5 min including two washes with 2 mL of cell staining buffer. The cell pellet was resuspended with 200 μL of cell staining buffer, and equal volumes were transferred into the appropriate tubes. To detect total Akt1 and Akt2, 2 μL of purified anti-Akt1 and anti-Akt2 (BioLegend, San Diego, CA, USA) was added, respectively. Additionally, 2 μL of purified anti-Akt phospho(Ser473) (BioLegend, San Diego, CA, USA) was added for the detection of either phosphorylated AKT1 or AKT2. Furthermore, a cocktail of isotype controls (5 μL of purified mouse IgG1, 5 μL of purified mouse IgG2b, and 1 μL of purified rabbit polyclonal isotype control antibody) was used to differentiate nonspecific background signals from specific antibody signals. The cells were incubated for 30 min at room temperature in the dark and then stained with 5 μL of phycoerythrin (PE) donkey anti-rabbit IgG and 2 μL of Alexa Fluor 488 Goat anti mouse IgG (BioLegend, San Diego, CA, USA). After 15 min of incubation in the dark, the cells were resuspended with 1000 μL of cell staining buffer and analyzed in the flow cytometer (CyFlow^®^, SL, Partec, GmbH, Muenster, Germany). Flow cytometric analysis was carried out in triplicate and performed by Partec Flomax software.

The inhibitory impact of the two triazolo[3,4-*b*]thiadiazole derivatives KA25 and KA39 on the phosphorylation of either Akt1 (p-Akt1) or Akt2 (p-Akt2) was determined by the following formulas: p-Akt1(%) or p-Akt2 (%) = [(mean-xtreated − isotypetreated)]/[(mean-xuntreated − isotypeuntreated)] × 100, where the isotype value is subtracted from the mean-x value of the corresponding condition, including treated and untreated cells. As the formulas indicate, the difference in value of treated cells is divided by the difference in value of untreated cells (control group) to estimate the extent of inhibition. In addition to the above formulas, the ratios phosphoAkt1/total Akt1 as well as phphoAkt2/total Akt2 were calculated and correlated to the corresponding ratios of untreated cells (control).

### 2.10. In Silico Studies

Molecular docking studies were carried out using Autodock Vina package [31], and crystal structures of Akt1 (Protein Data Bank (PDB) ID: 4GV1) and Akt2 (PDB ID: 2JDR) were taken from Protein Data Bank. For all PDB structures, during docking, co-crystallized inhibitors and water molecules were removed while polar hydrogens and Gasteiger charges were added using Auto Dock Tools 1.5.6. The triazolo[3,4-*b*]thiadiazole derivatives were built and optimized through energy minimization in ChemOffice 15.0. Prior to docking simulation, a grid box with spacing 1 Å and size of 20 × 20 × 20 (Å) around the ATP binding sites of all kinases was defined, and the “exhaustiveness” was set to 100. The docking poses with the lowest binding affinity were selected for presentation. PyMOL 2.1.1 viewer (Schrödinger, LLC) and BIOVIA/Discovery Studio were used for visualization of the docked complexes.

### 2.11. Statistical Analysis

Student’s *t*-test was used to compare the level of significance between the experimental groups. Differences with *p*-valued less than 0.05 were considered significant (*p* < 0.05, two tailed paired *t*-test). Microsoft Excel (Microsoft Hellas, Athens, Greece) was used.

## 3. Results

### 3.1. Synthesis of triazolo[3,4-b]thiadiazole Derivatives

#### 3.1.1. Synthesis of 3,6-disubstituted 1,2,4-triazolo-[3,4-*b*]-[1,3,4]-thiadiazoles

**Scheme 1:**
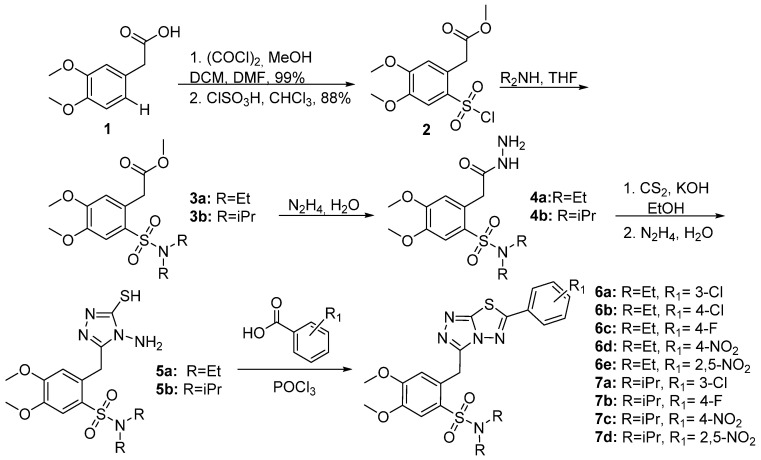
Synthesis of benzoic acid derivatives.

**Scheme 2:**
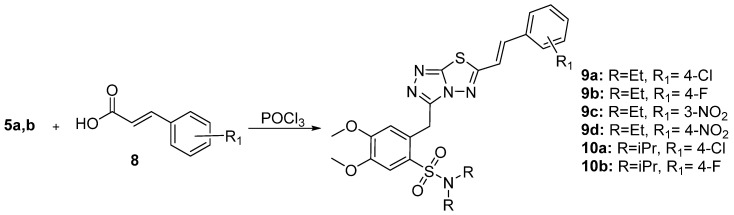
Synthesis of cinnamic acid derivatives.

Synthesis of the 1,2,4-triazolo-[3,4-*b*]-[1,3,4]-thiadiazoles was accomplished according to the synthetic methodology developed by our group (Scheme 1 and Scheme 2) [23]. After a reaction with oxalyl chloride, 2-(3,4-dimethoxyphenyl)acetic acid **1** was esterified and the corresponding methyl ester reacted with chlorosulfonic acid in CHCl_3_ to give methyl 2-(2-(chlorosulfonyl)-4,5-dimethoxyphenyl)acetate **2** in 88% yield. Subsequently, sulfonyl chloride **2** furnished the corresponding sulfonamides 3a,b after reaction with Et_2_NH or iPr_2_NH. Furthermore, these compounds reacted with hydrazine hydrate to give hydrazides 4a,b that reacted with KOH and CS_2_ in EtOH to give the potassium thiocarbamates. The latter products were cyclized in the presence of hydrazine hydrate to triazoles 5a,b [32]. The desired 1,2,4-triazolo[3,4-*b*]-[1,2,4]-thiadiazoles (6a–e, 7a–d, 9a–d, and 10a,b) were finally prepared via the reaction of triazoles 5a,b with POCl_3_ and various benzoic or cinnamic acids.

#### 3.1.2. In Vitro Anticancer Activity

The fifteen novel triazolo-thiadiazole derivatives and the previously described KA25, KA26, and KA39 were tested in vitro in two well-established human cancer cell lines: PC-3 and SKOV-3 cells. According to the first drug screening results, the 13 derivatives were inactive at the tested concentrations while the most active compounds (KA25, KA26, KA39, 6e, and 7d) were further tested in vitro in colorectal, ovarian, and prostate cancer cell lines. As Table 1, Table 2 and Table 3 show, the most potent anticancer activities in all human cancer cell lines were induced by the KA39, 6e, and 7d compounds (*p* < 0.001, two tailed paired *t*-test). All these compounds bear the 2,5*-*dinitrophenyl substituent on C-6 of the triazolo[3,4-*b*][1,3,4]thiadiazole core. Nevertheless, the triazolo[3,4-*b*]thiadiazole KA39 was significantly more active than 6e and 7d, which displayed less anticancer potency (Table 1, Table 2 and Table 3). Concerning KA25 and KA26 derivatives, both exhibited cytostatic activity in all human cancer cell lines, whereas KA25 induced cytotoxic effects only in ovarian and prostate cancer cells.

#### 3.1.3. In Vivo Acute Toxicity

The triazolo[3,4-*b*]thiadiazole derivatives (KA25, KA26, and KA39) were also evaluated for their in vivo acute toxicity, and such values are illustrated in Table 4. More specifically, the data showed that the LD10 values for KA25, KA26, and KA39 were 200, 100, and 70 mg/kg, respectively. The low acute toxicity in correlation with the in vitro anticancer activity provided evidence that the triazolo[3,4-*b*]thiadiazoles KA25, KA26, and KA39 are of high interest for cancer therapeutics since they promise high therapeutic ratios.

#### 3.1.4. In Vivo Anticancer Activity

**Figure 2:**
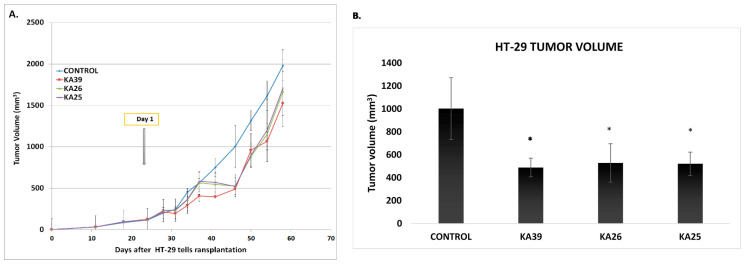
The in vivo antitumor activity of triazolo[3,4-*b*]thiadiazoles derivatives, KA39, KA26, and KA25 in mice bearing that HT-29 tumor. (**A**) In vivo growth inhibition of HT-29 human colorectal adenocarcinoma induced by KA39, KA26, and KA25 intraperitoneal administration. (**B**) The best response of tumor was induced by KA39 on day 18. Two-tailed paired *t*-test; * *p* < 0.05.

We also examined the antitumor activity of compounds KA25, KA39, and KA26 using the LD10 dose in an HT-29 human tumor (colorectal adenocarcinoma) xenograft in CB17 SCID mice. The results are presented in Figure 2A,B. As it is shown, the treatments with KA25, KA39, and KA26 caused statistically significant (*p* < 0.001) reductions in the size of HT-29 tumors in mice (Figure 2A). Eighteen days after treatment, the volume size of the tumors was 1003 ± 287.8 mm^3^ for the control group compared to 489.17 ± 81.5 mm^3^ for the group that received a single dose of KA39, to 528.5 ± 167.7 mm^3^ for the group that received a single dose of KA26, and to 521.6 ± 101.6 mm^3^ for the group that received a single dose of KA25 at the respective LD10 concentration levels (Table 4 and Figure 2B). With respect to tumor inhibition (%), the KA39, KA26, and KA25 derivatives displayed 67.6%, 62.4%, and 62.1%, respectively. As it is indicated by the TI% values, all compounds were equally effective in the actual reduction in HT-29 tumor volume and rate of tumor growth.

#### 3.1.5. Phosphorylation of Akt1 and Akt2 Isoforms

**Figure 3:**
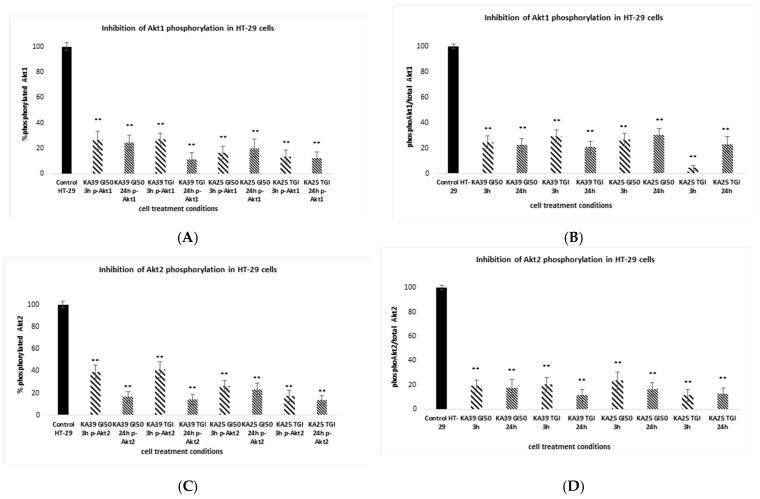
Inhibition of Akt1 and Akt2 phosphorylation in HT-29 cancer cells treated with the triazolo[3,4-*b*]thiadiazole derivatives KA25 and KA39 at GI50 and TGI concentrations for 3 and 24 h. (**A**) The diagram illustrates the KA25- and KA39-induced inhibition of Akt1 phosphorylation expressed as absolute values (%). (**B**) The diagram shows the induced inhibition of Akt1 phosphorylation by KA25 and KA39 according to the ratio phosphoAkt1/total Akt1 correlated with control (untreated cells). (**C**) The diagram displays the KA25- and KA39-induced inhibition of Akt2 phosphorylation expressed as absolute values (%). (**D**) The diagram presents the induced inhibition of Akt2 phosphorylation by KA25 and KA39 according to the ratio phosphoAkt2/total Akt2 correlated with the control (untreated cells). * *p* < 0.05, ** *p* < 0.01, and *** *p* < 0.001.

Today, it is well-established that the PI3K/Akt pathway regulates both repair and response to genotoxic damage. Therefore, we aimed to investigate the effect of KA39 and KA25 on Akt1 and Akt2 phosphorylation. Three colorectal cancer cell lines (HT-29, LoVo, and SW403) were treated with KA39 and KA25 at GI50 and TGI concentrations (μΜ) for 3 and 24 h. With respect to HT-29 cancer cells, treatment with KA39 at a TGI concentration of 15.9 μΜ for 3 and 24 h induced significant inhibition of Akt1 phosphorylation, decreasing the Akt1 phospho-form by 72.9% and 88.5%, respectively (*p* < 0.01) (Figure 3A).

Moreover, the phosphorylated form of Akt1 was reduced by 73.66% and 75.71% upon cell treatment with the referred derivative at a GI50 concentration of 11.5 μΜ for 3 and 24 h, respectively (*p* < 0.01) (Figure 3A). As Figure 3B presents, the ratio phosphoAkt1/total Akt1, correlated with control, is in line with the above-referenced absolute values (%), indicating that the Akt1 phospho-form was declined to a greater extent when cells were treated with KA39 at TGI concentration (μΜ) for 24 h (*p* < 0.01). The phosphorylated form of Akt1 was significantly diminished after HT-29 cells were treated with KA25 at a TGI concentration of 120 μΜ for 3 and 24 h, as the Akt1 phospho-form was decreased by 86.45% and 87.73%, respectively (*p* < 0.01) (Figure 3A). It is noteworthy that cell exposure to the same derivative at a GI50 concentration of 1 μΜ for 3 and 24 h impeded Akt1 phosphorylation (83.38% and 79.8%, respectively) to the same extent as TGI concentration (μΜ) at both treatment times (*p* < 0.01) (Figure 3A). The KA25-induced inhibition of Akt1 phosphorylation, estimated by the ratio phosphoAkt1/total Akt1, supports that the Akt1 phospho-form was significantly reduced under all treatment conditions whereas the greatest decrease was impelled after treatment at TGI concentration (μΜ) for 3 h (*p* < 0.01) (Figure 3B).

Phosphorylation of the Akt2 isoform was also inhibited by the two derivatives KA39 and KA25. More specifically, following treatment with KA39, at TGI (TGI = 15.9 μΜ) and GI50 concentrations (GI50 =11.5 μΜ) for 24 h, the Akt2 phospho-form was reduced by 86% and 83.55%, respectively (*p* < 0.01) (Figure 3C). Contrary to 24 h, Akt2 phosphorylation was less decreased when HT-29 cells were treated at TGI and GI50 concentrations (μΜ) for 3 h, diminishing the phosphorylated form of Akt2 by 58.87% and 60.76%, respectively (*p* < 0.01) (Figure 3C). The KA39-induced inhibition of Akt2 phosphorylation was also evaluated by the ratio phosphoAkt2/total Akt2, according to which the Akt2 phospho-form was significantly decreased under all treatment conditions, with no remarkable fluctuations arising (*p* < 0.01) (Figure 3D). However, according to the ratio phosphoAkt2/total Akt2, KA39 impelled a greater inhibition of Akt2 phosphorylation after cell exposure a TGI concentration (μΜ) for 24 h (Figure 3D). Furthermore, KA25 resulted in significant inhibition of Akt2 phosphorylation after cells were treated at TGI concentration (TGI = 120 μΜ) for 3 and 24 h, reducing the Akt2 phospho-form by 82.6% and 86.4%, respectively (*p* < 0.01) (Figure 3C). The inhibition of Akt2 phosphorylation, induced by KA25, was also recorded when cells were treated at GI50 concentration (GI50 =1 μΜ) for 3 and 24 h, diminishing the Akt2 phospho-form by 73.74% and 76.9%, respectively (*p* < 0.01) (Figure 3C). According to the ratio phosphoAkt2/total Akt2, KA25 declined the phosphorylated form of Akt2 to a similar extent, though cell exposure at GI50 concentration (μΜ) for 3 h exhibited a rather low inhibitory effect on Akt2 phosphorylation (*p* < 0.01) (Figure 3D).

Further investigation showed that phosphorylation of the two Akt isoforms (Akt1 and Akt2) was significantly inhibited by KA39 and KA25 in SW403 and LoVo cancer cells cancer cells (See the Supplementary Materials, Figures S47A–D and S48A–D). Even though KA39 inhibited Akt1 phosphorylation to a similar extent, the greatest reduction in Akt1 phospho-form (%) was observed upon treatment at TGI concentration (μΜ) for 24 h in all three cancer cell lines. On the other hand, KA39 particularly decreased the phosphorylated form of Akt2 (%) when the cells were treated at both concentrations (μΜ) for 24 h. The KA39-induced inhibition of Akt1 and Akt2 phosphorylation, expressed as absolute values (%), also corresponds to the ratios phopshoAkt1/total Akt1 and phosphoAkt2/total Akt2, respectively. In relation to KA25, the Akt1 and Akt2 phospho-forms were remarkably reduced (%) under all treatment conditions, though cell exposure at TGI concentration (μΜ) for 3 and 24 h impeded phosphorylation of the two Akt isoforms to a greater extent. Nevertheless, according to the ratio phosphoAkt1/total Akt1, Akt1 phosphorylation was significantly inhibited upon cell treatment at TGI concentration (μΜ) for 3 h in all three cancer cell lines. On the other hand, as the ratio phosphoAkt2/total Akt2 suggests, the KA25-induced inhibition of Akt2 phosphorylation was similar in HT-29 and SW403 cells whereas the greatest decrease in the Akt2 phospho-form was found in LoVo cells after treatment at TGI concentrations (μΜ) for 3 and 24 h.

#### 3.1.6. In Silico Studies

**Figure 4:**
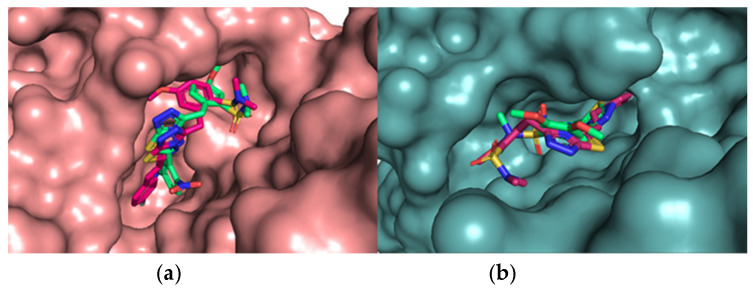
Structures of KA25 (magenta) and KA39 (green) occupying the ATP binding site of (**a**) 4GV1 (Akt1, pink) and (**b**) 2JDR (Akt2, blue).

**Figure 5:**
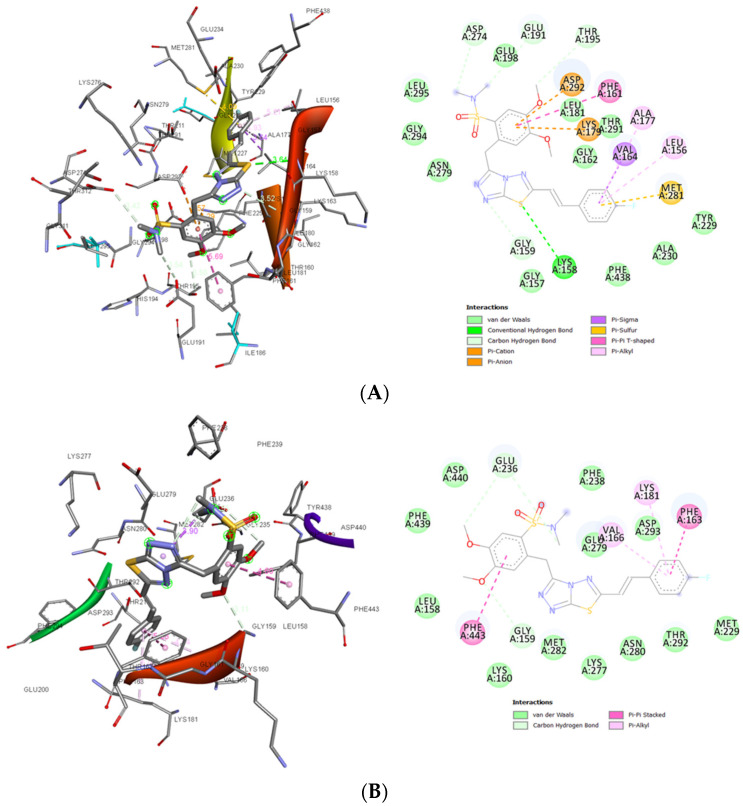
Binding mode of **KA25** (**left**) in the active site of (**A**) Akt1 (4GV1) and (**B**) Akt2 (2JDR). Dotted lines in different colors reflect various types of interaction such as hydrogen bonding, charge or polar interactions, and van der Waals and π-sigma interactions.

**Figure 6:**
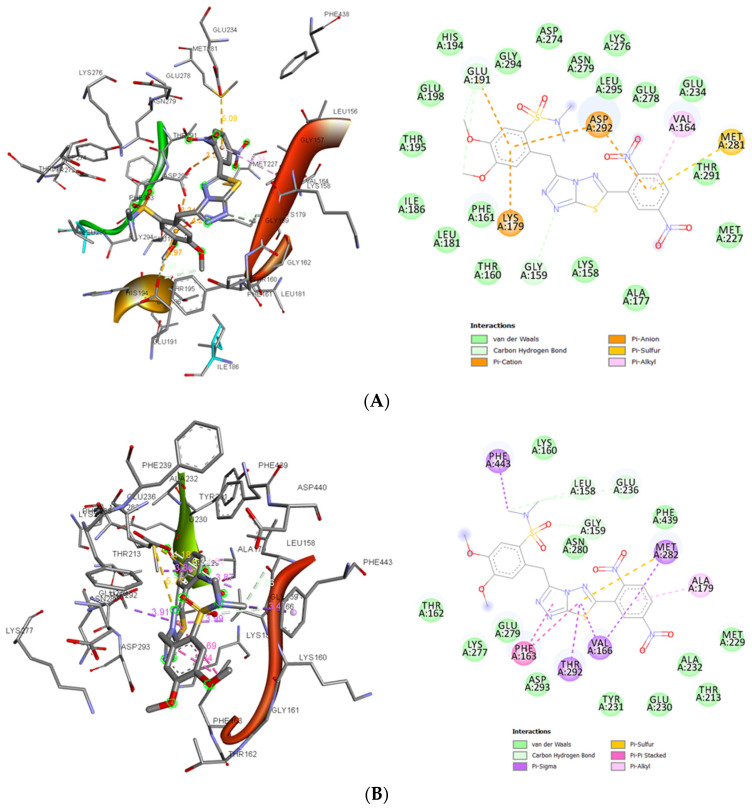
Binding mode of KA39 (**right**) in the active site of (**A**) Akt1 (4GV1) and (**B**) Akt2 (2JDR). Dotted lines in different colors reflect various types of interaction such as hydrogen bonding, charge or polar interactions, and van der Waals and π-sigma interactions.

In a following step, in order to understand the interactions of KA25 and KA39 with Akt1 and Akt2 kinases, molecular docking studies were performed using the Autodock Vina software 1.1.2 [31]. Prior to docking, the docking protocol was validated. The co-crystallized inhibitors were removed and re-docked into the catalytic sites of Akt1 (PDB ID: 4GV1) and Akt2 (PDB ID: 2JDR) to determine the reliability of the applied docking protocol. As illustrated in Figure S1, the docking predictions of ligands in both crystal structures using Autodock Vina had acceptable root-mean-square deviation (RMSD) values. The data from the docking experiments of KA25 and KA39 are demonstrated below. The selected derivatives (KA25 and KA39) have high binding energies (<−8.5 kcal/mol) on the two Akt isoforms (Table 5, Table S1 and Table S2). Both compounds were found to occupy the ATP binding site of the tested kinases, as presented in Figure 4, and the most stable binding modes among the top docking poses are illustrated in Figure 5 and Figure 6. The docking analysis revealed that the Akt1–KA25 complex had the most promising results. Compound KA25 was stabilized by various van der Waals interactions and one hydrogen bond with a Lys158 (3.64 Å) residue of Akt1. It also displayed electrostatic interactions with Asp292 and Lys179 residues as well as hydrophobic interactions with Phe161, Leu156, Val164, and Ala177. The 2,5-dinitrophenyl group of KA39 is oriented towards the hinge region of Akt1, but does not interact with it, while the 4-fluorophenyl group of KA25 interacts with the hinge region of Akt1 at Ala230 and Tyr229. The interactions between KA39 and the binding site of Akt1 contain electrostatic interactions with Lys179, Glu191, and Asp292 and hydrophobic interactions with Val164. The opposite is observed for Akt2; KA39 displays hydrophobic interactions with Phe443, Ala179, Val166, Met282, Phe163, and Thr292; and KA25 stands outside the hinge and interacts with Phe443, Phe163, Val166, and Lys181. According to the selected poses, it is clear that the synthesized derivatives are oriented in the catalytic pocket of Akt kinases and may act as ATP pocket binders.

## 4. Discussion

Aberrations in the PI3K/Akt signal transduction pathway are a very common phenomenon in human carcinogenesis, with over half of tumors showing irregular Akt activation [33]. More precisely, the overexpression of certain oncogenes or a lack of tumor suppressor genes influences the biological activity of the PI3K/Akt network, stimulating cancer cell growth. For instance, mutations of Epidermal Growth Factor Receptor, EGFR/PI3K, loss of the tumor suppressor protein phosphatase and tensin homologue (PTEN), and mutations or amplifications of Akt itself enhance Akt’s signaling in cancer cells [34,35]. Amplified and mutated Akt isoforms play crucial roles in tumorigenesis, while Akt2 is particularly involved in EMT transition and metastasis. Regarding mutations, Akt1 is the most frequently mutated isoform compared with Akt2, in which mutations do not occur at high frequency and cannot be considered as activating [36,37]. The most prevalent mutation in Akt1, E17K, is located within the PH domain and is found in several solid tumors including breast (5.9%), colorectal (1.6%), lung (0.6%), and ovarian cancers (0.8%) as well as in melanoma (0.5%) [38,39]. Akt1E17K mutation, induced by the substitution of lysine to glutamic acid at amino acid 17, leads to a pathological translocation of Akt1 to the plasma membrane and to alterations in PIP specificity as binding to PIP2 seems to be highly increased. Given that Akt1 normally binds to PIP3, further utilization of PIP2 in the case of Akt1E17K enhances Akt1′s signaling [39,40,41].

Tumor aggressiveness as well as poor survival rates are particularly linked to Akt2′s amplification and overexpression [9]. According to earlier studies, mutations in the Akt2 isoform are quite rare whilst amplifications in the isoform have been detected in 16% of pancreatic cancers; 16% of uterine cancers; 13% of breast cancers; and 5–10% of ovarian, lung, and bladder cancers. The role of Akt2 in oncogenesis is reflected by a reduction in cell growth and invasiveness induced by siRNA depletion of Akt2 in cells that overexpress Akt. On the other hand, an amplified Akt1 is less frequent since it has been found only in 20% of neuroendocrine prostate cancers, 10% of pancreatic cancers, and 3–5% of breast and serous ovarian cancers [42]. Altogether, Akt is strongly implicated in many types of cancer and therefore has been validated as a therapeutic target nearly two decades ago [43].

The present study attempts to explore the inhibitory impact of KA25 and KA39 derivatives on Akt1 and Akt2 phosphorylation in three colorectal cancer cell lines (HT-29, LoVo, and SW403) with specific PIK3CA and KRAS statuses (Table 6). More specifically, two of the referred cell lines, HT-29 and SW403, harbor mutations in PIK3CA, p.P449T and p.Q546K, respectively, while LoVo cells retain a wild-type PIK3CA (https://cancer.sanger.ac.uk/cosmic, accessed on 4 September 2020). With regard to PIK3CA mutations, most of them are concentrated in hotspots within two domains: the helical (exon 9) and kinase (exon 20) [44]. Nonetheless, four additional missense mutations have been detected including p.P449T, which belongs to gain-of-function mutations and contributes to enhanced PIK3CA kinase activity (>2-fold) compared to the wild-type PIK3CA. In general terms, gain-of-function mutations in PIK3CA are known for triggering the PI3K kinase activity and consequently activating its downstream molecules such as Akt [45]. Further studies have indicated that rare PIK3CA mutants, including p.Q546K, possess a high oncogenic potency as well as hyperactivate PI3K/Akt downstream signaling cascade [46]. Due to the fact that HT-29 and SW403 cell lines contain gain-of-function mutations in PIK3CA, the levels of phosphorylated Akt may be elevated in both cell lines in comparison to the wild-type PIK3CA expressed by the LoVo cell line.

With respect to KRAS, two of the three cell lines, LoVo and SW403, are KRAS-mutant-bearing p.G13D and p.G12V mutations, respectively (Table 6). Experimental studies using these cell lines showed that neither p.G12V nor p.G13D KRAS mutations stimulate the phosphorylation of Akt to a greater extent than wild-type KRAS cells. Moreover, it has been also demonstrated that Akt phosphorylation was decreased in KRAS-G12V cells compared to KRAS wild-type cell lines [52,53,54]. Consequently, the LoVo and SW403 cell lines (KRAS-mutant) probably express similar or lower levels of phosphorylated Akt than HT-29 cells (KRAS wild-type). As far as cell sensitivity to Akt inhibitors is concerned, recent studies support that cell lines with PI3K and/or PTEN mutations display a higher susceptibility to this type of inhibitor than cells with KRAS and/or v-raf murine sarcoma viral oncogene homolog B (BRAF) mutations [55]. More specifically, Akt inhibitors show greater selectivity and potency in cells with increased Akt kinase activity resulting from mutations in PI3K or PTEN. Later studies implied that cell sensitivity to allosteric Akt1/2 inhibitors is significantly associated with Akt phosphorylation in human cancer cell lines, leading to the conclusion that cell lines with elevated levels of phosphorylated Akt are more susceptible to the pharmacologic inhibition of Akt1/2. However, the cell lineage may affect the pharmacologic sensitivity to Akt1/2 inhibition, for instance, PIK3CA-mutant breast cancer cell lines are more sensitive than PIK3CA-mutant colon cancer cell lines. Altogether, high levels of phosphorylated Akt signify AKT dependency in PIK3CA-mutant cancer cells [56,57].

On the other hand, Akt inhibitors, utilized in the inhibition of downstream effector pathways, have not shown an impressive antitumor efficacy on KRAS-mutant tumors [58]. As observed, cells with mutated KRAS and/or BRAF are less sensitive to Akt inhibitors since no alterations in the activation of MAPK pathway have arisen [55]. A relevant example that correlates PIK3CA and RAS mutations with sensitivity to Akt inhibitors concerns the AZD5363 compound; PIK3CA and PTEN mutations are strongly associated with sensitivity to this Akt inhibitor, regardless of RAS status [59]. Additionally, tumors bearing an overactivated PI3K/Akt pathway without any EGFR, RAS, or BRAF mutations depend more on this pathway and become more sensitive to selective Akt inhibitors [60]. It is noteworthy that both of the referred to KRAS mutations (p.G12V and p.G13D) are associated with resistance to anti-EGFR therapy via intracellular pathways, restricting the administration of anti-EGFR monoclonal antibodies (mAbs) to patients bearing KRAS wild-type tumors [61,62]. Based on PIK3CA and KRAS statuses, we could put forward the hypothesis that KA25- and KA39-induced inhibition of Akt1 and Akt2 phosphorylation might be more effective in HT-29 (PIK3CA-mutant; KRAS wild-type) than those of LoVo cells (PIK3CA wild-type; KRAS-mutant). Regarding SW403 cells, in which PIK3CA and KRAS mutations concur, Akt1 and Akt2 phosphorylation could be impeded similarly to either HT-29 or LoVo cells. Nevertheless, both methods of estimation (% inhibition and ratios correlated with control) indicate that both triazolo[3,4-*b*]thiadiazole derivatives exhibited significant inhibitory activity against Akt1and Akt2 phosphorylation in all three cancer cell lines. It is notable that the inhibition of Akt1 and Akt2 phosphorylation, induced by KA25 and KA39, was fairly similar in all three cell lines carrying PIK3CA and/or KRAS mutations. Nonetheless, it could be suggested that the reduction in phosphorylated Akt (Akt1/2) was slightly greater in LoVo (mutated KRAS) and SW403 (mutated KRAS and PIK3CA) cells compared to the HT-29 cell line (mutated PIK3CA).

Furthermore, pharmacokinetic studies on the tested compounds are currently under investigation. Premature experimental data showed that these compounds have poor water solubility (log mol/L = −4.4 to −3.5), high CaCO_2_ permeability (log Papp in 10^−6^ cm/s = −0.135 to 0.965), and high intestinal absorption (94–100%). These compounds present low distribution volumes with high binding to serum proteins (72–92%), are unable to penetrate the CNS, and are poorly distributed to the brain. They are not substrates of P-glycoprotein but they inhibit both glycoproteins I and II. They metabolize in liver, and they are substrates of CYP3A4 but inhibit CYP2C19 and CYP2C19. Their total clearance in rats was 2.2–5.7 mL/min/kg.

It is interesting to note that a large number of small molecules are proven to inhibit Akt in vitro and in vivo, though only a limited number of these have entered clinical trials [48]. Ongoing clinical studies indicate that the tested Akt inhibitors are not effective as single agents, displaying rather low therapeutic responses. Furthermore, even if there is increasing knowledge concerning Akt functions and activation, there are no Akt inhibitors approved for oncologic use. For example, miltefosine, the only Akt inhibitor approved, is intended for treating visceral and cutaneous leishmaniasis and not cancer disease [38,48]. Therefore, the discovery of drugs such as KA39 constitutes a valuable tool in the research area of Akt inhibitors due to its high anticancer potency and multitarget behavior.

## 5. Conclusions

In this work, preliminary mechanistic studies showed that KA25 and KA39 exhibit time- and concentration-dependent inhibition of Akt Ser-473 phosphorylation while in silico studies pointed out the potentials of KA25 and KA39 to act as ATP binders in the catalytic sites of Akt1 and Akt2. Among all the tested triazolo[3,4-*b*]thiadiazole derivatives, KA39 is the most potent antitumor agent since it induced the most significant cytostatic and cytotoxic activities against all cancer cell lines. According to the current findings and prior studies that displayed triazolo[3,4-*b*]thiadiazole derivatives as topoisomerase IIα inhibitors, it can be assumed that this particular class of chemical compounds behave as multitarget anticancer agents. More precisely, KA39 exhibited a significant inhibitory impact on the activities of other biological systems such as those of topoisomerase IIα (topIIα). Phosphorylation at the Ser1106 site, closely associated with the catalytic activity of topIIα, triggers the decatenation function of the enzyme. However, treatment with the KA39 compound induced a significant inhibition of the phosphorylation at Ser1106 and, as a consequence, attenuated topIIα’s enzymatic activity [23]. Furthermore, it has been shown that KA39 interferes with the catalytic cycle of topIIα, leading to the formation of supercoiled DNA, either by blocking the interaction of the enzyme with its plasmid substrate or by strangling its ATPase action. Further investigations are currently being carried out in order to explore new targets of triazolo[3,4-*b*]thiadiazole compounds.

## Figures and Tables

**Table 1:** Growth inhibition/cytostatic (GI50 and TGI) and cytocidal/cytotoxic (IC50) effects induced by triazolo[3,4-*b*]thiadiazole derivatives KA25, KA26, and KA39 on nine human cancer cell lines. GI_50_: 50% growth inhibition; TGI: Total growth inhibition; IC_50_: the concentration that causes 50% cell death.

Cell Line	KA25			KA26			KA39		
	GI_50_ (μΜ)	TGI (μΜ)	IC_50_ (μΜ)	GI_50_ (μΜ)	TGI (μΜ)	IC_50_ (μΜ)	GI_50_ (μΜ)	TGI (μΜ)	IC_50_ (μΜ)
**SKOV-3**	15 ± 0.5	50 ± 1.5	85 ± 2.1	20 ± 0.5	>100	>100	7 ± 0.2	12 ± 0.8	25 ± 1.4
**UWB 1.289**	2 ± 0.5	5 ± 0.7	62 ± 1.2	29 ± 0.7	53 ± 1	70 ± 1.4	6 ± 0.2	10 ± 0.8	22 ± 1.2
**UWB1.289+BRCA1**	4.0 ± 0.5	13 ± 0.8	38 ± 1.1	42 ± 0.5	65 ± 0.7	>100	6 ± 0.5	11 ± 0.7	94 ± 2.3
**HT-29**	1 ± 0.3	>100	>100	2 ± 0.76	>100	>100	11.5 ± 0.8	15.9 ± 0.55	19.5 ± 0.9
**LS174T**	9.3 ± 2.0	100 ± 0.1	>100	8 ± 1.5	>100	>100	12 ± 1.5	17 ± 1.3	21.5 ± 1.5
**SW403**	4.8 ± 0.8	>100	>100	11 ± 0.8	>100	>100	5.2 ± 0.2	7.9 ± 0.7	10 ± 0.76
**LoVo**	8 ± 0.76	13.8 ± 0.52	>100	15 ± 0.8	>100	>100	2.2 ± 0.2	5.5 ± 0.1	10.5 ± 0.15
**PC-3**	14 ± 1.0	22 ± 0.8	>100	42.5 ± 1.5	>100	>100	5 ± 0.15	8 ± 0.1	12 ± 0.1
**DU-145**	12.8 ± 1.3	13.2 ± 0.5	27 ± 0.3	7 ± 1.0	70 ± 1.3	>100	5.8 ± 0.2	8 ± 0.4	10.3 ± 1.8

**Table 2:** Growth inhibition/cytostatic (GI50 and TGI) and cytocidal/cytotoxic (IC50) induced by the thirteen newly synthesized triazolo[3,4-*b*]thiadiazoles on two human cancer cell lines.

Compound	PC-3			SKOV-3		
	GI_50_ (μΜ)	TGI (μΜ)	IC_50_ (μΜ)	GI_50_ (μΜ)	TGI (μΜ)	IC_50_ (μΜ)
**9b**	42 ± 1.0	>100	>100	>100	>100	>100
**10b**	31 ± 1.1	>100	>100	50 ± 2.0	>100	>100
**9a**	11 ± 1.0	>100	>100	84 ± 2.0	>100	>100
**10a**	2 ± 0.5	84 ± 2.0	>100	76 ± 6.0	>100	>100
**6b**	32 ± 0.4	56 ± 0.6	104 ± 0.8	36 ± 0.1	60 ± 0.8	>100
**6a**	25 ± 1.4	112 ± 2.5	>100	6 ± 0.3	14 ± 2.5	>100
**7a**	20 ± 0.8	>100	>100	16 ± 0.8	46 ± 1.5	>100
**6c**	90 ± 2.0	>100	>100	>100	>100	>100
**7b**	25 ± 0.5	80 ± 1	>100	80 ± 0.8	>100	>100
**6d**	50 ± 0.5	>100	>100	80 ± 0.5	>100	>100
**7c**	23 ± 0.8	102 ± 1.2	>100	15 ± 1.4	28 ± 2.2	66 ± 2.5
**9c**	38 ± 2.0	>100	>100	>100	>100	>100
**9d**	30 ± 0.5	>100	>100	>100	>100	>100

**Table 3:** Growth inhibition/cytostatic (GI50 and TGI) and cytocidal/cytotoxic (IC50) induced by tri-azolo[3,4-*b*]thiadiazole derivatives 6e and 7d on five human cancer cell lines.

Cell Lines	6e			7d		
	GI_50_ (μΜ)	TGI (μΜ)	IC_50_ (μΜ)	GI_50_ (μΜ)	TGI (μΜ)	IC_50_ (μΜ)
**SKOV-3**	18 ± 0.7	24 ± 1.2	38 ± 1.8	13 ± 0.5	17 ± 1.0	23 ± 1.4
**PC-3**	11 ± 1.0	17 ± 1.0	26.5 ± 0.9	8 ± 1.0	11 ± 1.04	22 ± 0.3
**DU-145**	13 ± 0.5	18.3 ± 0.2	28 ± 0.2	6.8 ± 0.8	14 ± 0.5	21.3 ± 0.9
**DLD-1**	25 ± 2.0	37 ± 2.0	53.6 ± 1.05	7.3 ± 0.75	12 ± 0.5	22 ± 1.0
**HT-29**	18 ± 1.0	29 ± 0.5	44 ± 1.0	17.9 ± 2.51	26 ± 1.52	40 ± 1.0

**Table 4:** Acute toxicity of compounds in CB17 severe Combined Immunodeficiency (SCID) mice.

Compound	LD_10_ (mg/kg)	LD_50_ (mg/kg)
**KA39**	70	100
**KA25**	200	360
**KA26**	100	160

**Table 5:** Molecular docking results showing the binding energy between Akt1 and Akt2 with the synthesized derivatives KA25 and KA39.

Compounds	Akt1	Akt2
	Docking Score (Kcal/mol)	Docking Score (Kcal/mol)
**KA25**	−9.5	−8.9
**KA39**	−8.7	−8.6

**Table 6:** Description of histotypes and special characteristics of the three human colorectal cancer cell lines in which the inhibition of Akt (Akt1/2) phosphorylation, induced by KA25 and KA39, was investigated.

Cancer Type	Human Cell Line Designation	KRAS Status	PIK3CA Status	References
Colorectal adenocarcinoma	HT-29	wild-type	p.P449T	[47,48]
Colorectal adenocarcinoma, Duke’s type C, grade IV	LoVo	p.G13D	wild-type	[47,48]
Colorectal adenocarcinoma Dukes’ type C, grade III	SW403	p.G12V	p.Q546K	[49,50,51]

## Data Availability

The data presented in this study are available in supplementary material.

## Supplementary Materials

The following are available online at https://www.mdpi.com/article/10.3390/pharmaceutics13040493/s1, Figure S1, Tables S1 and S2 Docking Studies; ^1^Η-ΝΜR, ^13^C-NMR and ESI-MS spectra for the synthesized compounds Figure S2–S46, Tables S3–S24 analytical methods for LC-MS; Figure S47, Figure S48 Inhibition of Akt1 and Akt2; Figure S49–S75 flow cytometric analysis data.
