# Supplementary material for: Anticancer Activity of Triazolo-Thiadiazole Derivatives and Inhibition of AKT1 and AKT2 Activation
Source: Pharmaceutics. 2021 Apr 5;13(4):493. doi: 10.3390/pharmaceutics13040493 (PMC8066331; doi:10.3390/pharmaceutics13040493)
Supplement: Supplementary file 1 [file pharmaceutics-13-00493-s001.pdf]

pharmaceutics

MDPI

Supplementary Materials:Anticancer Activity of Triazolo-Thiadiazole Derivatives and Inhibition of AKT1 and AKT2 Activation

Dimitrios T. Trafalis, Sofia Sagredou, Panayiotis Dalezis, Maria Voura, Stella Fountoulaki, Nikolaos Nikoleousakos, Konstantinos Almpanakis, Maria V. Deligiorgi and Vasiliki Sarli

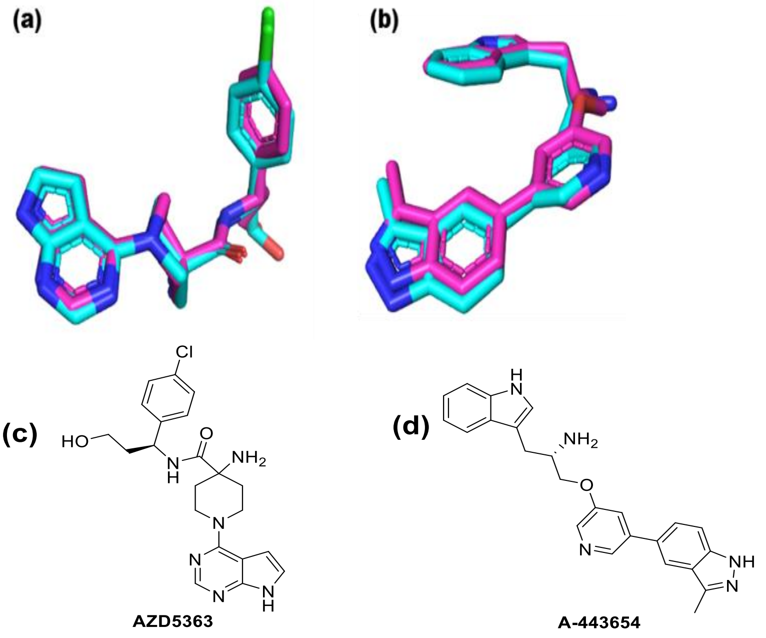

Figure S1. Comparison between crystal structure (cyan) with the predicted binding mode in Autodock Vina (magenta) for structures (**a**) 4GV1 in AKT1 and (**b**) 2JDR in AKT2. Structures of the co-crystallized inhibitors (**c**) AZD5363 of Akt1 in 4GV1 and (**d**) A-443654 of Akt2 in 2JDR.

Table S1. Interacting residues of Akt1 with KA25 and KA39.

Compound	Akt1 Interacting Residues
KA25	Asn279, Gly294, Leu295, Asp274, Glu198, Glu191, Thr195, Asp292, Phe161, Leu181, Lys179, Thr291, Ala177, Gly162, Val164, Leu156, Met281, Tyr229, Ala230, Phe438, Lys158, Gly157, Gly159
KA39	Asp274, Asn279, Lys276, Leu295, Asp292, Glu278, Glu234, Val164, Met281, Thr291, Met227, Ala177, Lys158, Gly159, Thr160, Lys179, Phe161, Leu181, Ile186, Thr195, Glu198, His194, Glu191, Gly294

Table S2. Interacting residues of Akt2 with KA25 and KA39.

Compound	Akt2 Interacting Residues
KA25	Glu236, Phe238, Glu279, Val166, Asp293, Lys181, Phe163, Met229, Thr292, Asn280, Lys277, Met282, Gly159, Lys160, Phe443, Leu158, Phe439, Asp440
KA39	Gly159, Asn280, Phe439, Met282, Ala179, Met229, Ala232, Thr213, Glu230, Tyr231, Val166, Thr292, Asp293, Phe163, Glu279, Lys277, Thr162, Phe443, Lys160, Leu158, Glu236

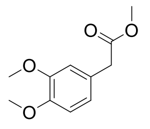

S1

(a)

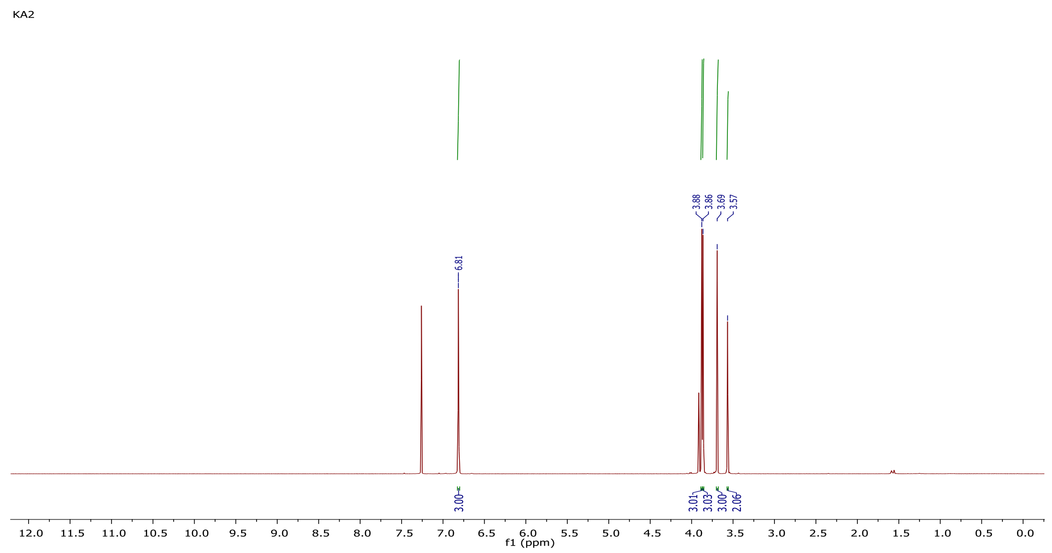

(b)

Figure S2. (a) S1**; (b)** ^{1}H-NMR for S1.

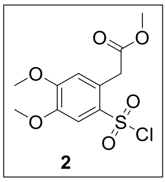

(a)

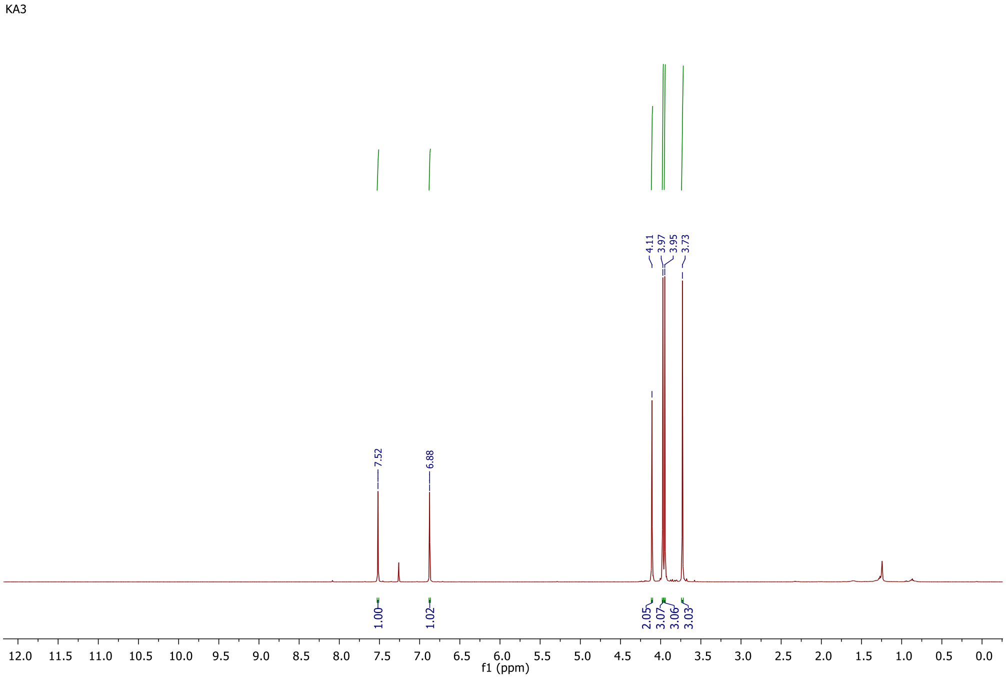

(b)

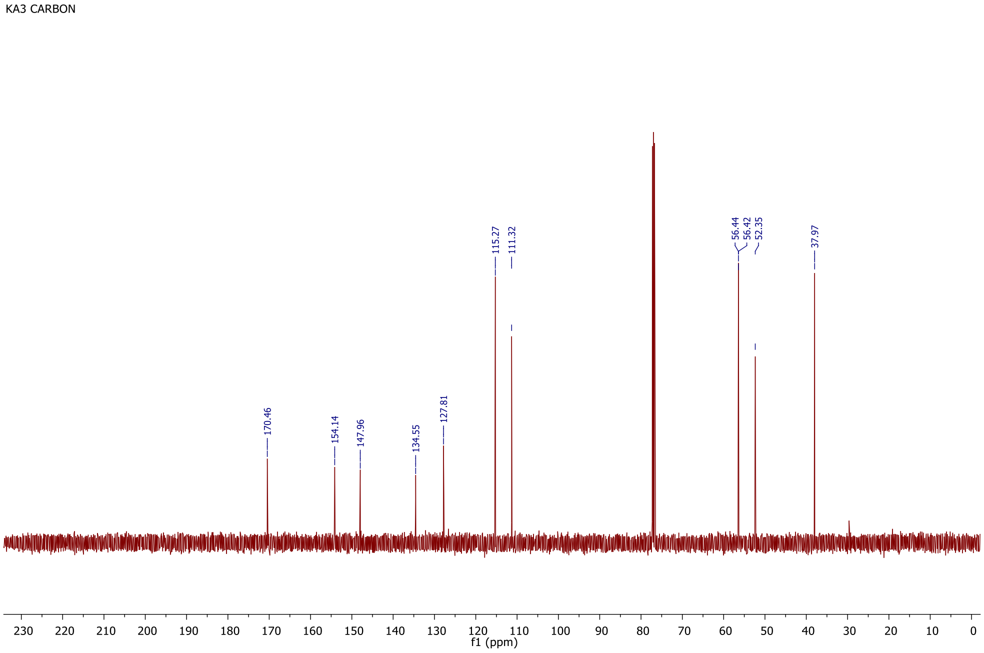

(c)

Figure S3. (a) 2; **(b)** 1H-NMR and **(c)** 13C-NMR spectra for 2.

Analytical method for LC-MS for 2

LC-20AD Shimadzu connected to Shimadzu LCMS-2010EV
Mobile Phase: methanol
LC isocratic
HPLC column: SUPELCO Discovery C18, 25cm × 4.6mm, 5µm
Flow rate: 0.4 mL/min
Column temperature: 26 °C
UV detector: 254 nm
MS detector: 1.65 K
Run time: 20 min
Retention time: 16.5 min

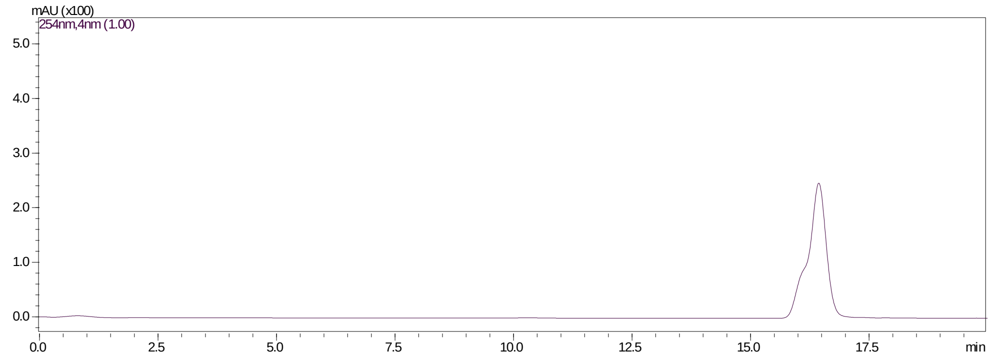

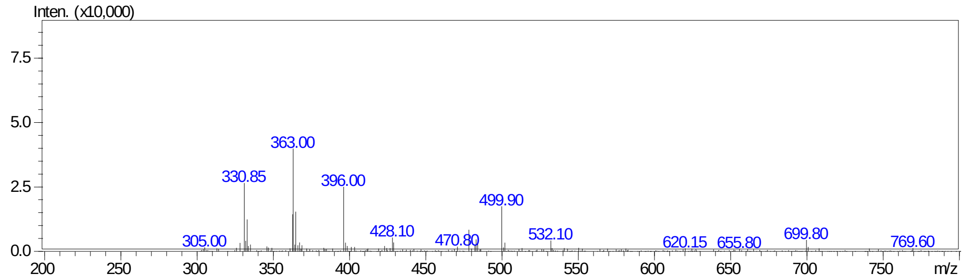

Figure S4. LC/ESI-MS analysis for 2; ESI-MS, positive mode: *m*/*z* calcd mass for $C_{11}H_{13}ClNaO_6S$ $[M+Na]^+$ = 331.0, was found 330.85.

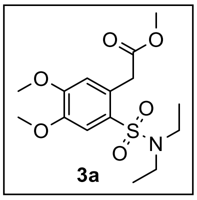

(a)

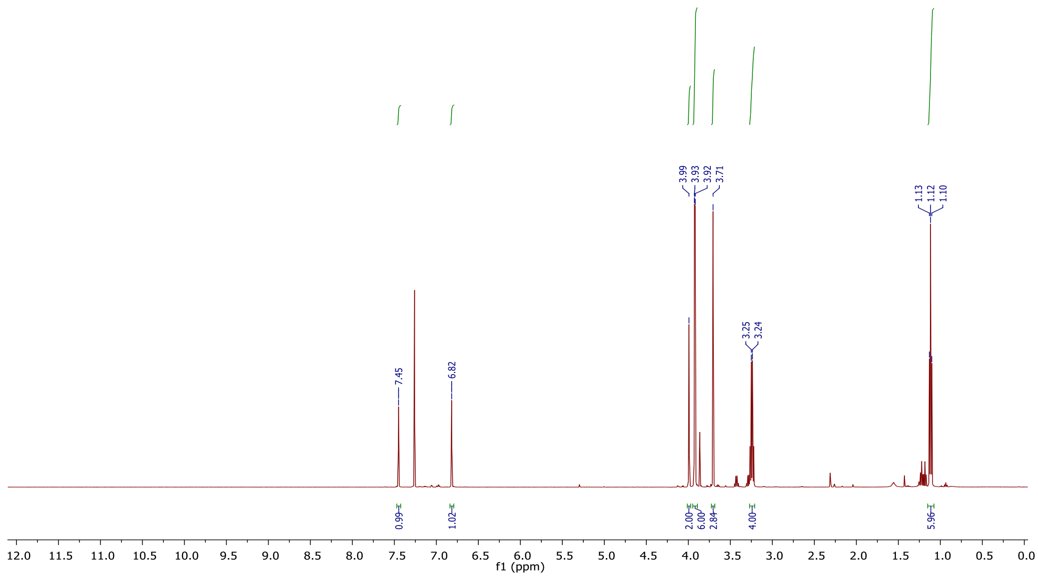

(b)

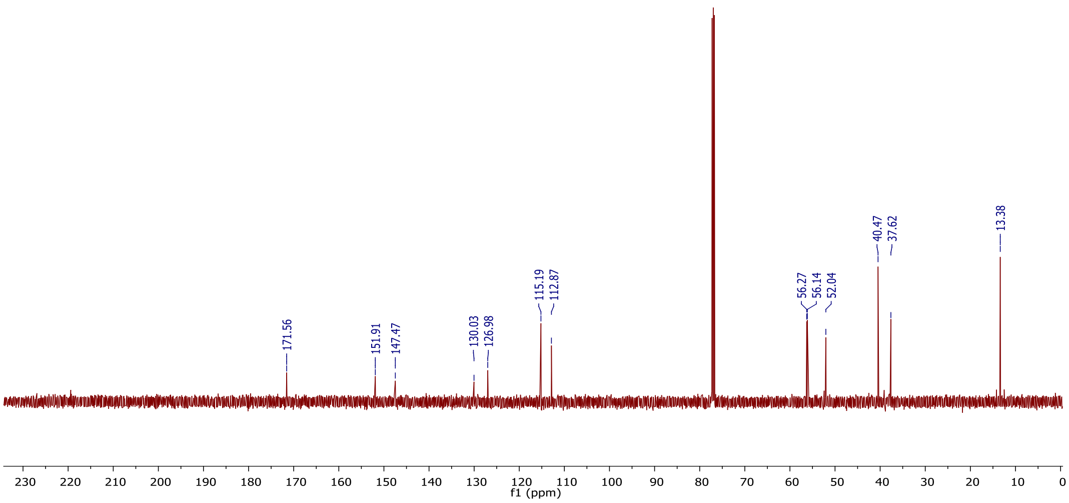

(c)

.**Figure S5.** (**a**) 3a; **(b)** ^{1}H-NMR and (**c**) ^{13}C-NMR spectra for 3a.

Analytical method for LC-MS for 3a

LC-20AD Shimadzu connected to Shimadzu LCMS-2010EV
Mobile Phase: methanol
LC isocratic
HPLC column: SUPELCO Discovery C18, 25cm × 4.6mm, 5µm
Flow rate: 0.4 mL/min
Column temperature: 26 °C
UV detector: 254 nm
MS detector: 1.65 K
Run time: 20 min
Retention time: 14.3 min

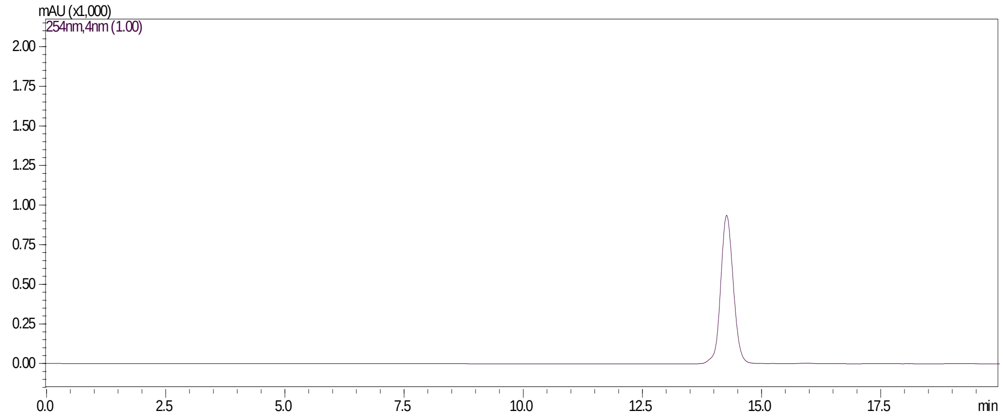

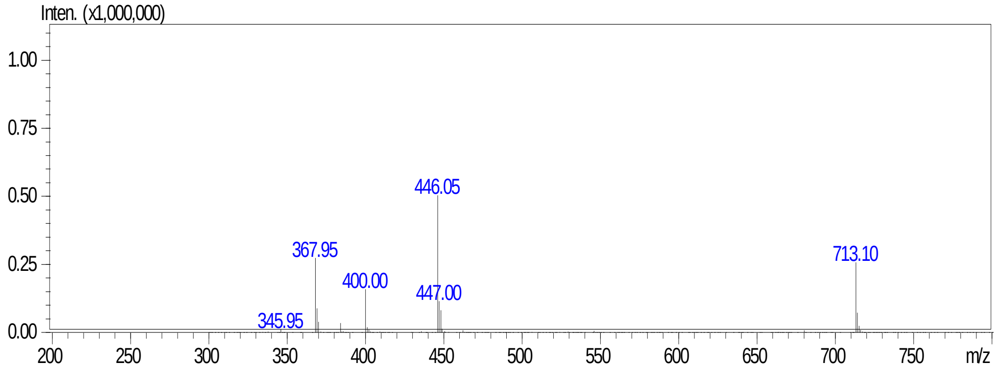

Figure S6. LC/ESI-MS analysis for 3a; ESI-MS, positive mode: *m*/*z* calcd mass for C15H23NNaO6S $[M+Na]^+$ = 368.11, was found 367.95.

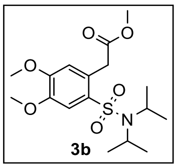

(a)

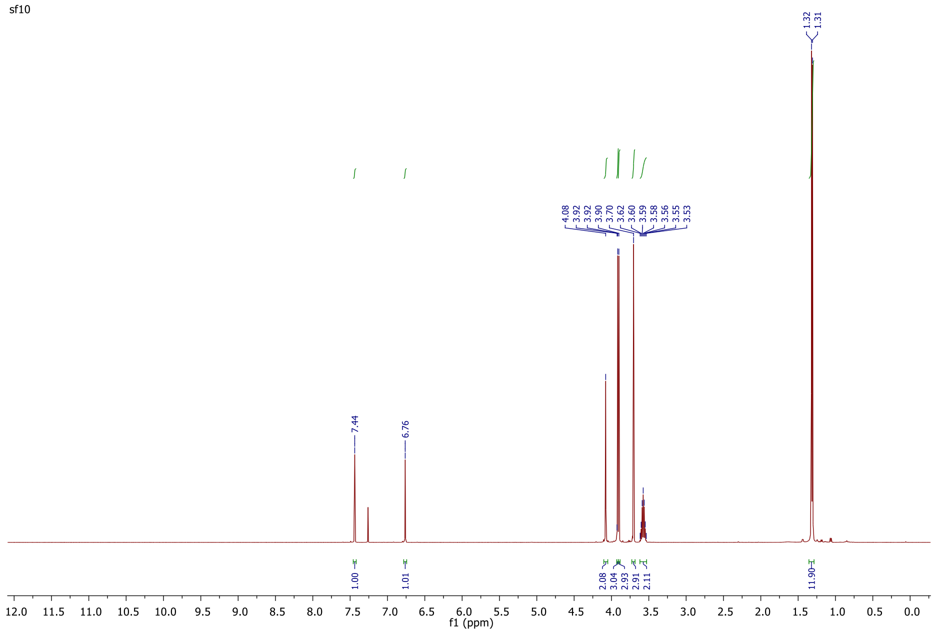

(b)

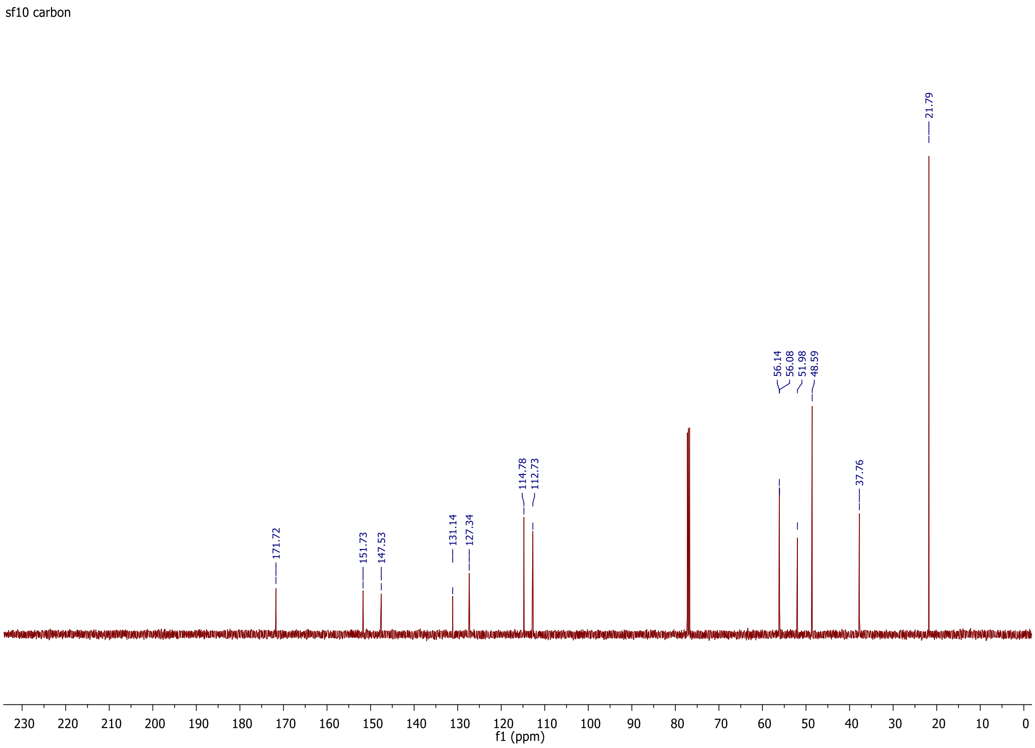

(c)

Figure S7. (a) 3b; (**b**) ^{1}H-NMR and (**c**) ^{13}C-NMR spectra for 3b.

Analytical method for LC-MS for 3b

LC-20AD Shimadzu connected to Shimadzu LCMS-2010EV
Mobile Phase: methanol
LC isocratic
HPLC column: SUPELCO Discovery C18, 25cm × 4.6mm, 5µm
Flow rate: 0.4 mL/min
Column temperature: 26 °C
UV detector: 254 nm
MS detector: 1.65 K
Run time: 20 min
Retention time: 16.2 min

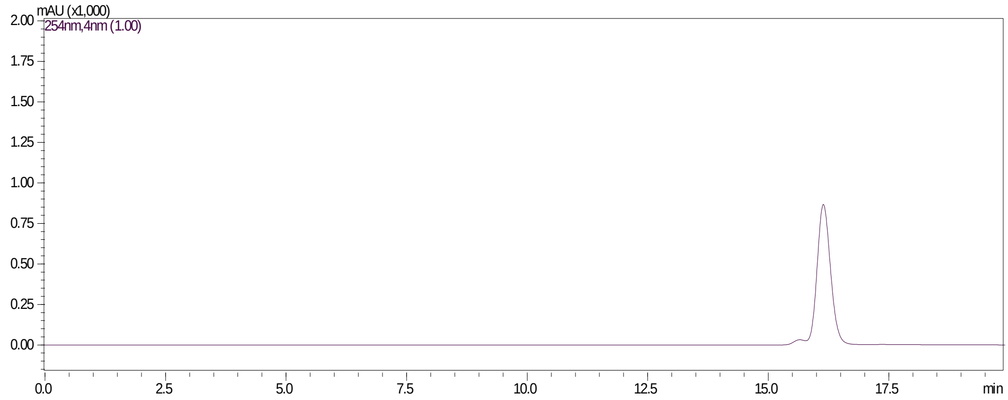

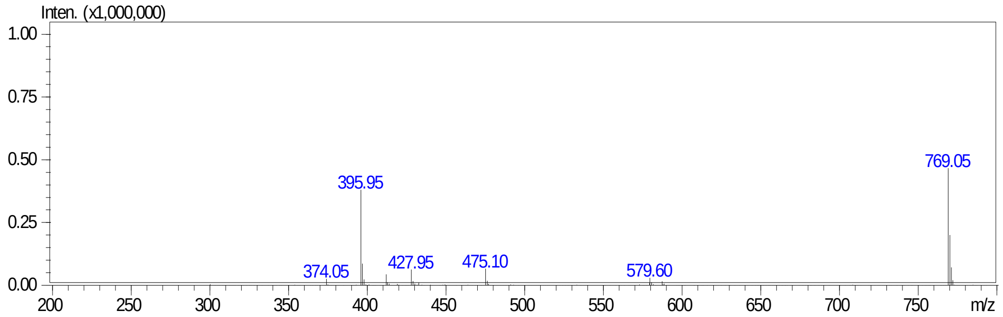

Figure S8. LC/ESI-MS analysis for 3b; ESI-MS, positive mode: *m*/*z* calcd mass for C17H27NNaO6S $[M+Na]^+$ = 396.15, was found 395.95.

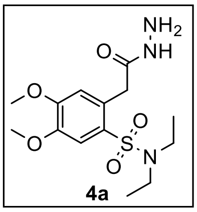

(a)

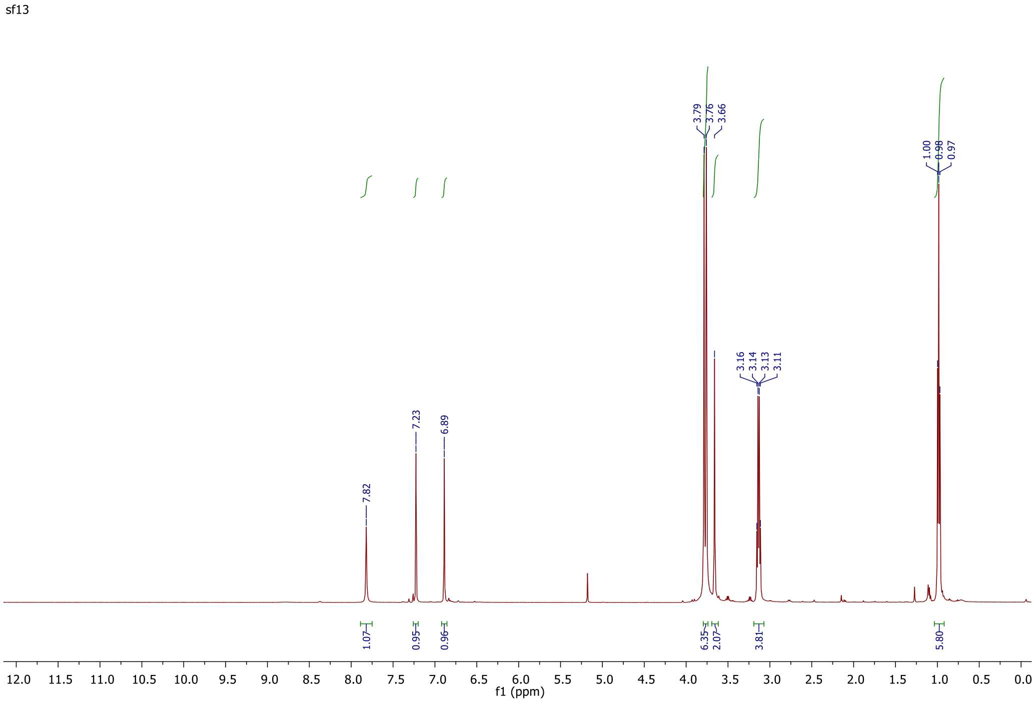

(b)

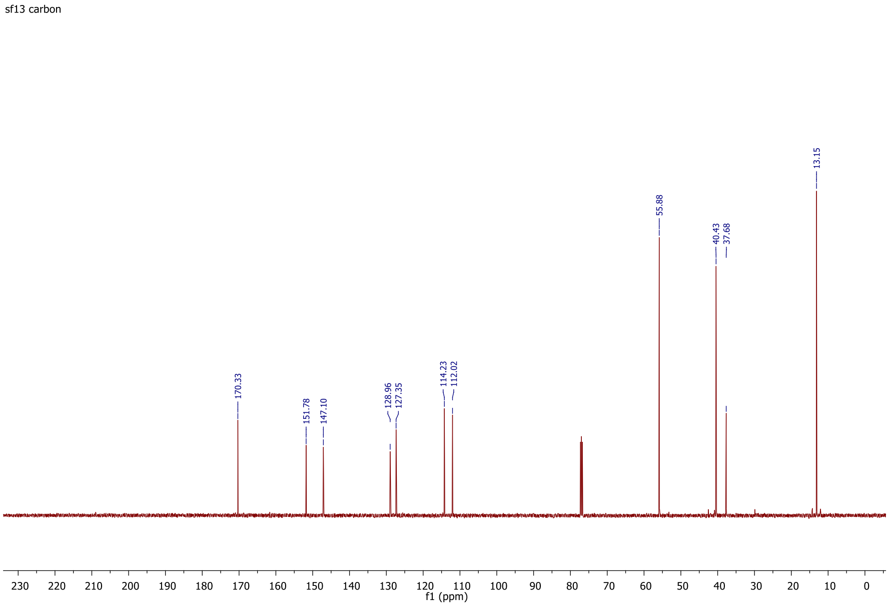

(c)

Figure S9. (**a**) 4a; **(b)** ^{1}H-NMR and (**c**) ^{13}C-NMR spectra for 4a.

Analytical method for LC-MS for 4a

LC-20AD Shimadzu connected to Shimadzu LCMS-2010EV
Mobile Phase: methanol
LC isocratic
HPLC column: SUPELCO Discovery C18, 25cm × 4.6mm, 5µm
Flow rate: 0.4 mL/min
Column temperature: 26 °C
UV detector: 254 nm
MS detector: 1.65 K
Run time: 20 min
Retention time: 16 min

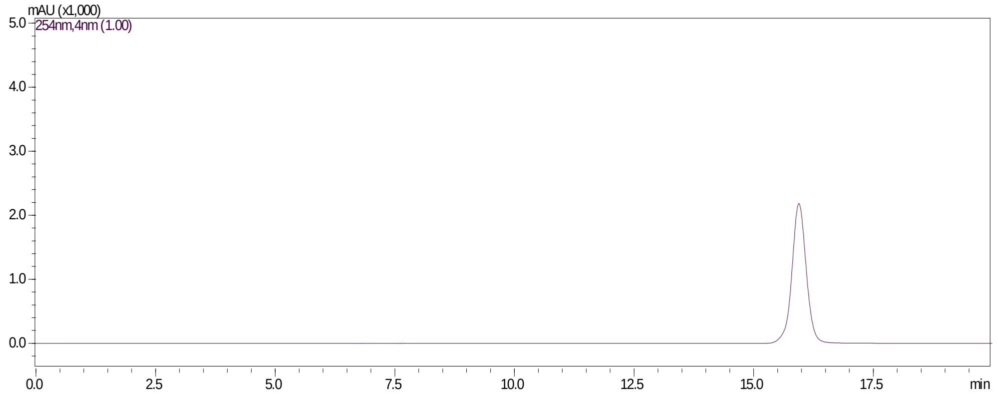

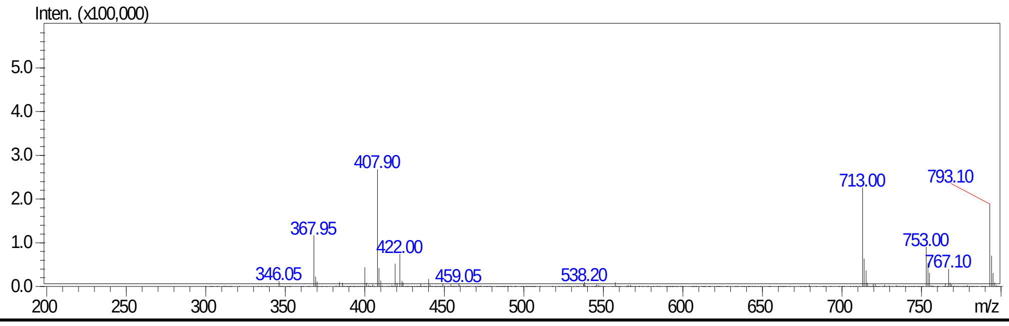

Figure S10. LC/ESI-MS analysis for 4a; ESI-MS, positive mode: *m*/*z* calcd mass for C14H23N3NaO5S $[M+Na]^+$ = 368.13, was found 367.95.

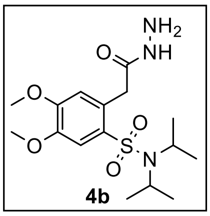

(a)

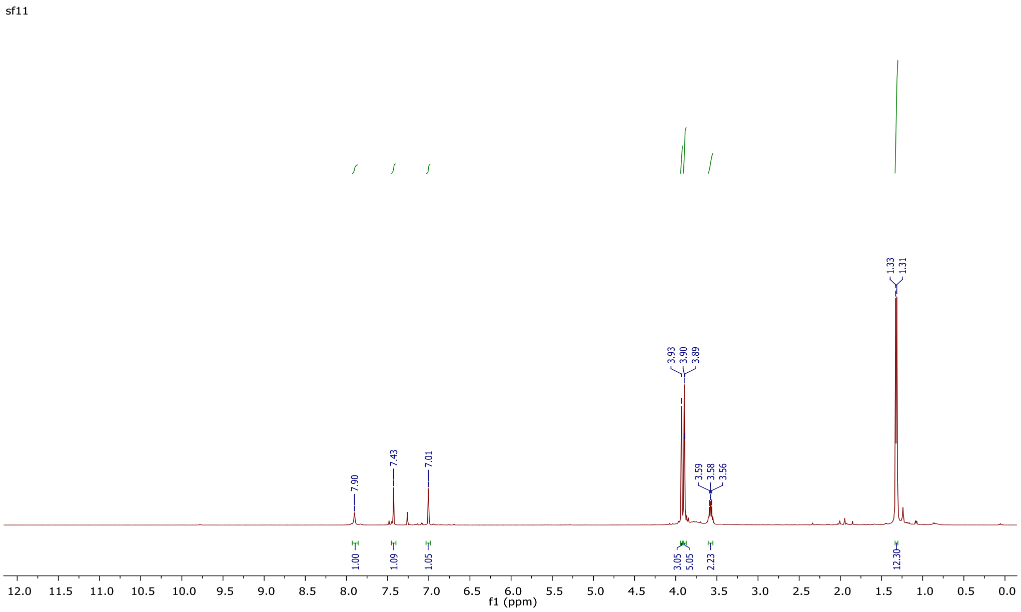

(b)

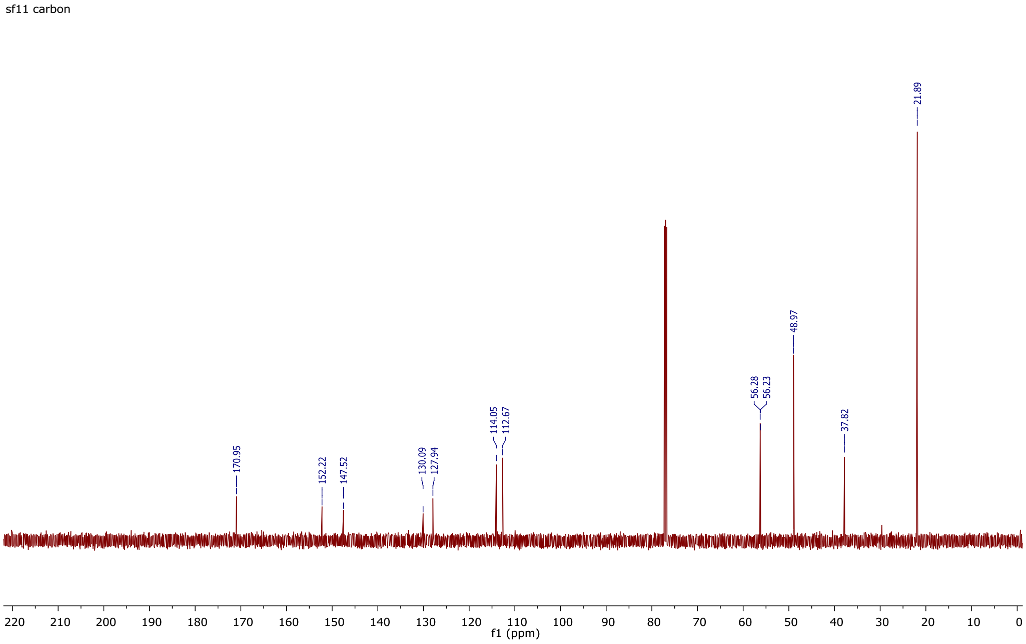

(c)

Figure S11. (**a**) 4b; **(b)** ^{1}H-NMR and (**c**) ^{13}C-NMR spectra for 4b.

Analytical method for LC-MS for 4b

LC-20AD Shimadzu connected to Shimadzu LCMS-2010EV
Mobile Phase: methanol
LC isocratic
HPLC column: SUPELCO Discovery C18, 25cm × 4.6mm, 5µm
Flow rate: 0.4 mL/min
Column temperature: 26 °C
UV detector: 254 nm
MS detector: 1.65 K
Run time: 20 min
Retention time: 16 min

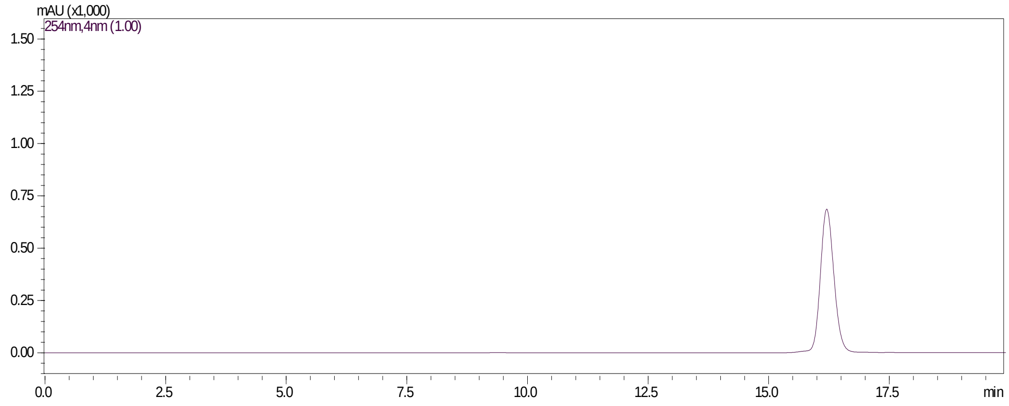

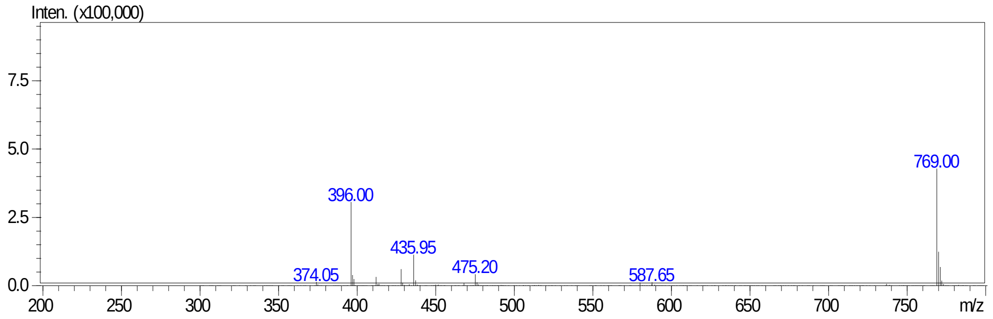

Figure S12. LC/ESI-MS analysis for 4b; ESI-MS, positive mode: *m*/*z* calcd mass for C16H27N3NaO5S $[M+Na]^+$ = 396.16, was found 396.00.

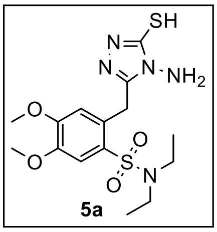

(a)

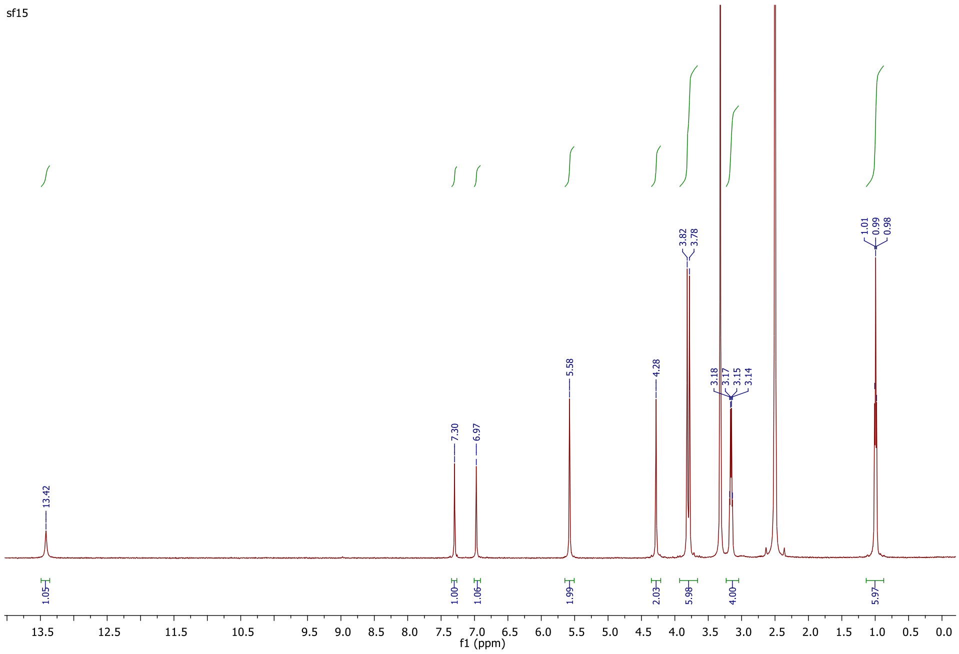

(b)

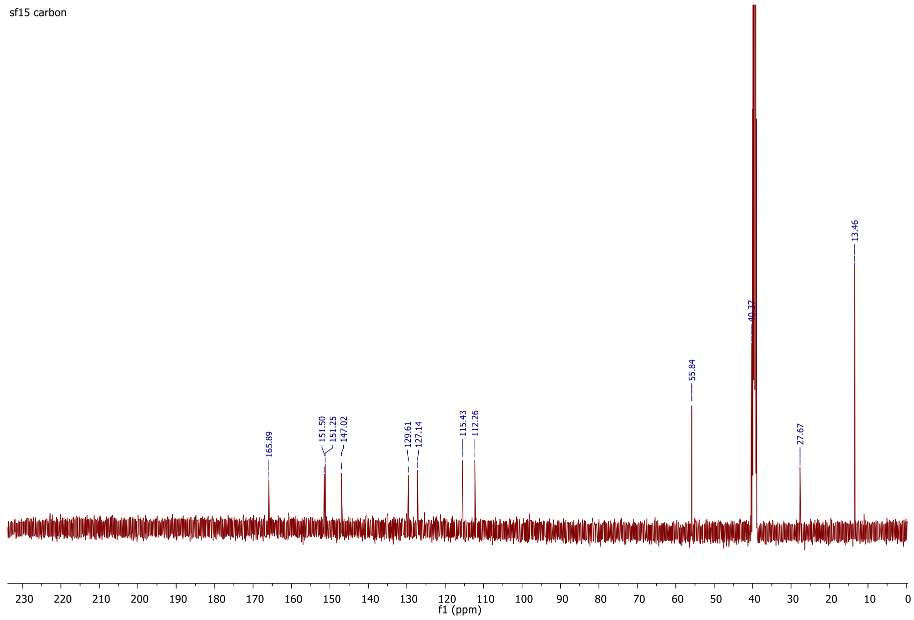

(c)

Figure S13. (**a**) 5a; **(b)** ^{1}H-NMR and (**c**) ^{13}C-NMR spectra for 5a.

Analytical method for LC-MS for 5a

LC-20AD Shimadzu connected to Shimadzu LCMS-2010EV
Mobile Phase: methanol
LC isocratic
HPLC column: SUPELCO Discovery C18, 25cm × 4.6mm, 5µm
Flow rate: 0.4 mL/min
Column temperature: 26 °C
UV detector: 254 nm
MS detector: 1.65 K
Run time: 11 min
Retention time: 8 min

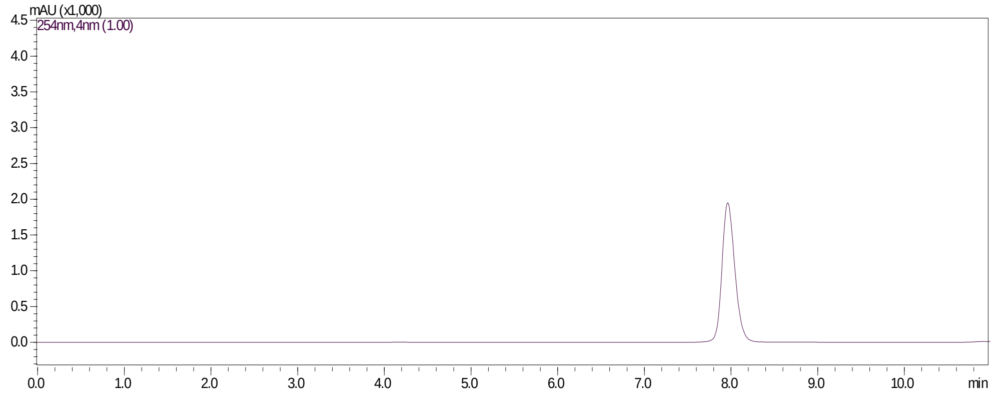

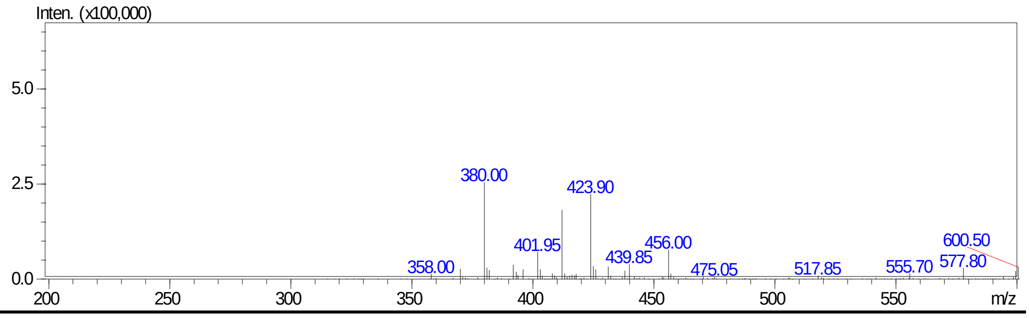

Figure S14. LC/ESI-MS analysis; ESI-MS, positive mode: *m*/*z* calcd mass for C15H23N5NaO4S2 $[M+Na]^+ = 424.11$, was found 423.90.

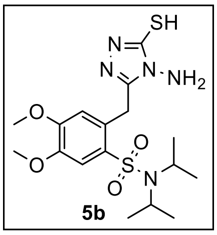

(a)

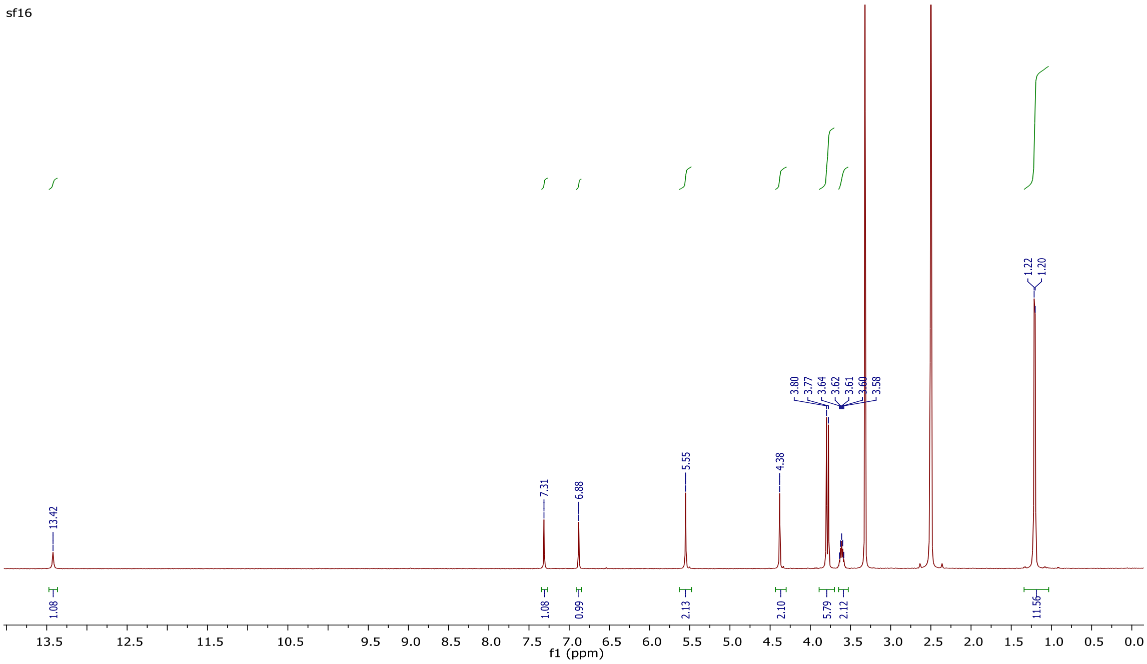

(b)

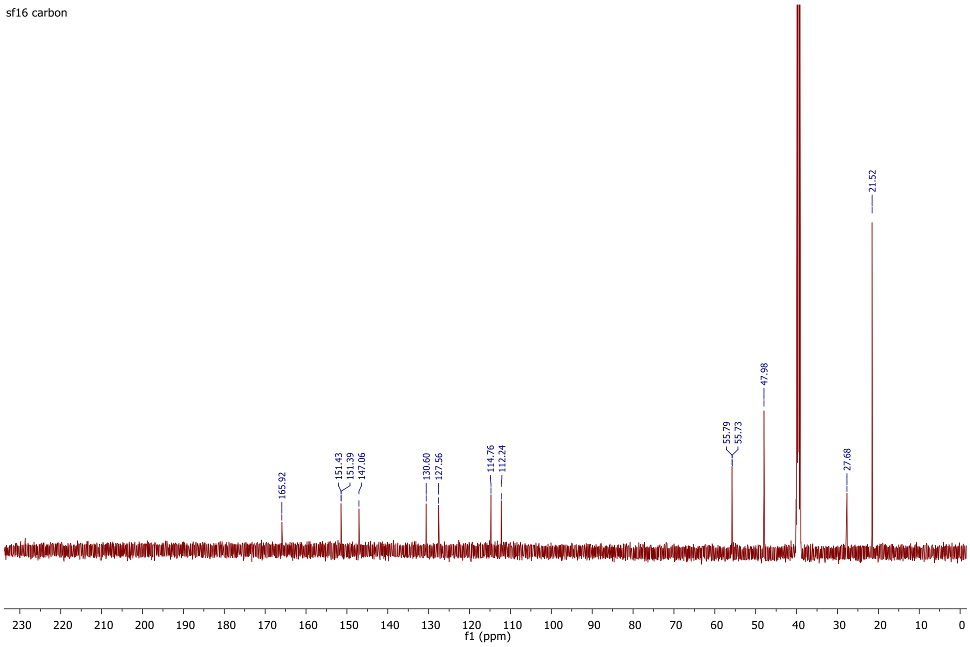

(c)

Figure S15. (**a**) 5b; **(b)** ^{1}H-NMR and (**c**) ^{13}C-NMR spectra for 5b.

Analytical method for LC-MS for 5b

LC-20AD Shimadzu connected to Shimadzu LCMS-2010EV
Mobile Phase: methanol
LC isocratic
HPLC column: SUPELCO Discovery C18, 25cm × 4.6mm, 5μm
Flow rate: 0.4 mL/min
Column temperature: 26 °C
UV detector: 254 nm
MS detector: 1.65 K
Run time: 20 min
Retention time: 11.8 min

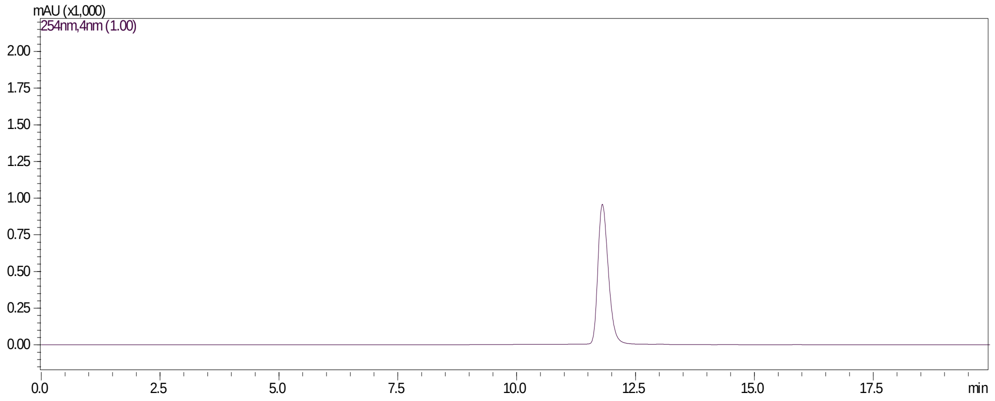

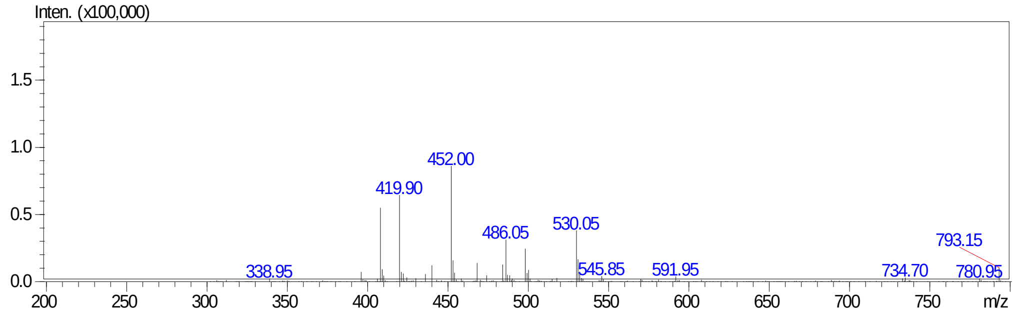

Figure S16. LC/ESI-MS analysis for 5b; ESI-MS, positive mode: *m*/*z* calcd mass for C17H27N5NaO4S2 $[M+Na]^+$ = 452.14, was found 452.00.

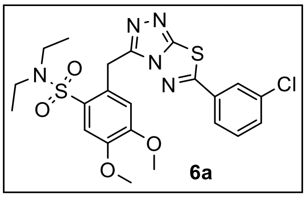

(a)

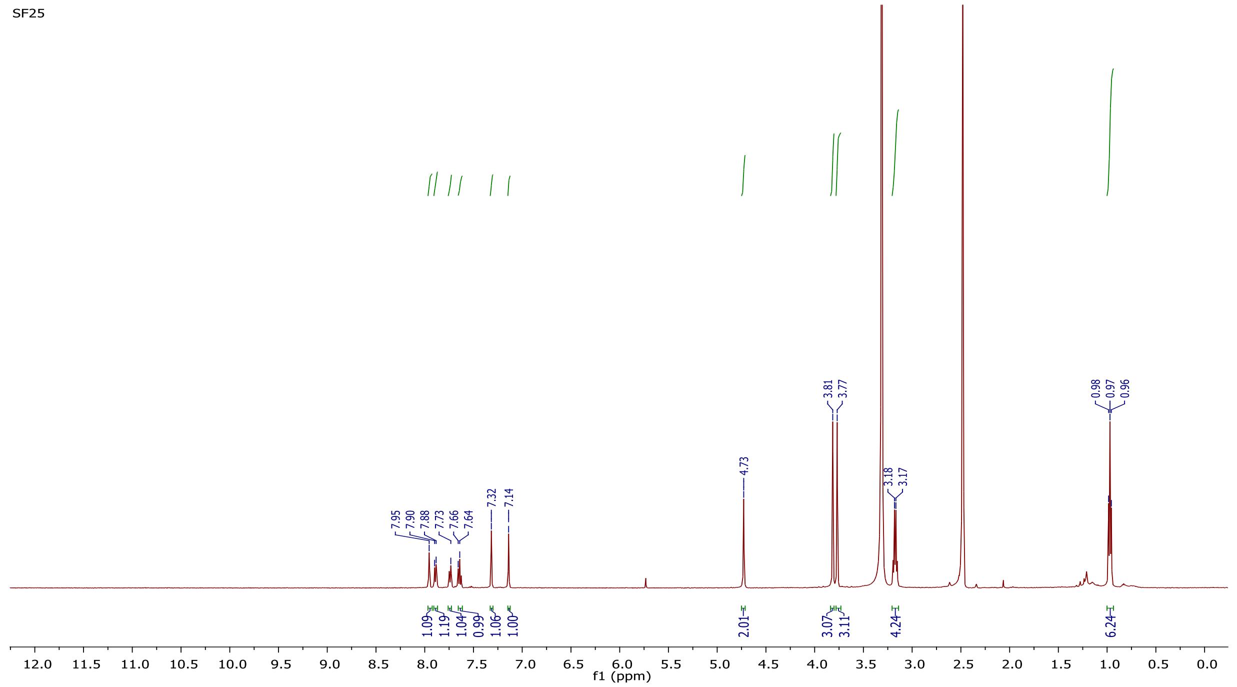

(b)

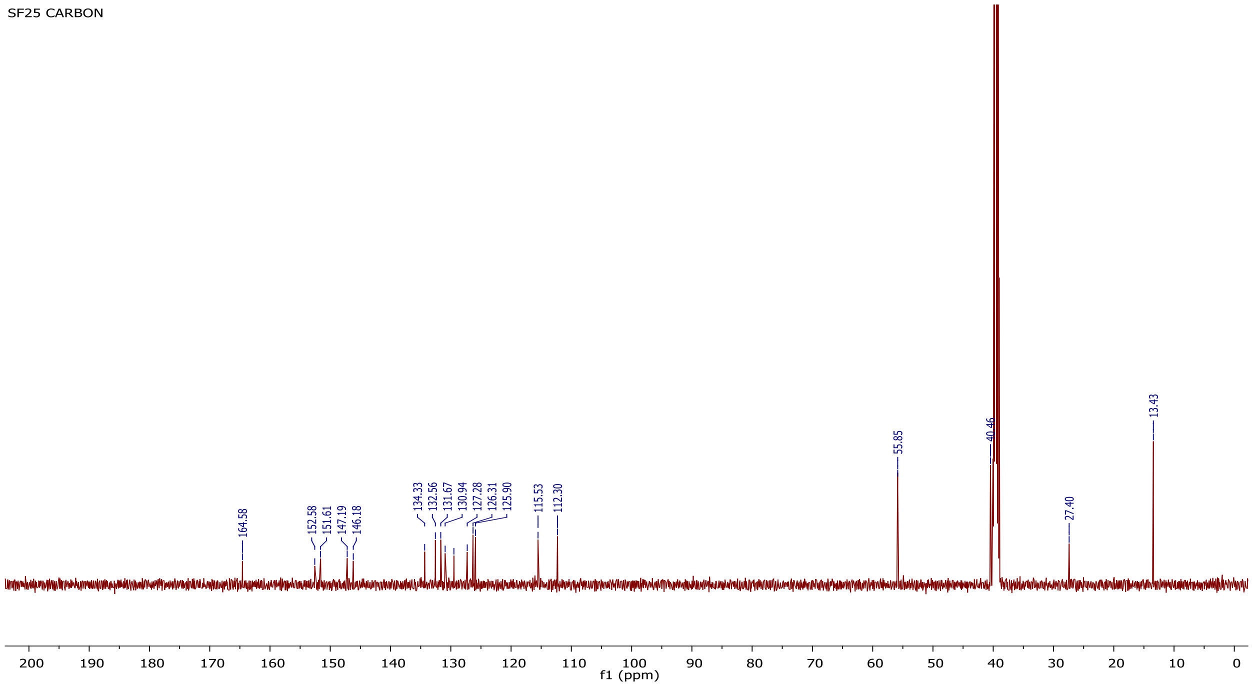

(c)

Figure S17. (**a**) 6a; **(b)** ^{1}H-NMR and (**c**) ^{13}C-NMR spectra for 6a.

Analytical method for LC-MS for 6a

LC-20AD Shimadzu connected to Shimadzu LCMS-2010EV
Mobile Phase: methanol
LC isocratic
HPLC column: SUPELCO Discovery C18, 25cm × 4.6mm, 5μm
Flow rate: 0.4 mL/min
Column temperature: 26 °C
UV detector: 254 nm
MS detector: 1.65 K
Run time: 20 min
Retention time: 11.3 min

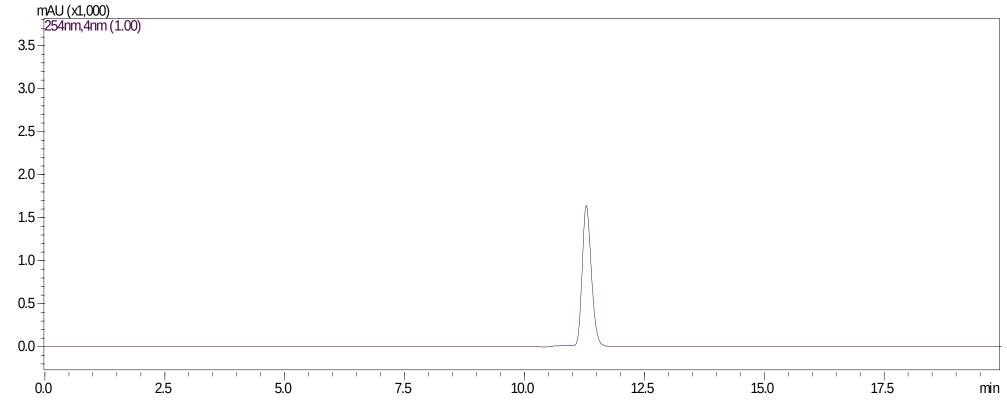

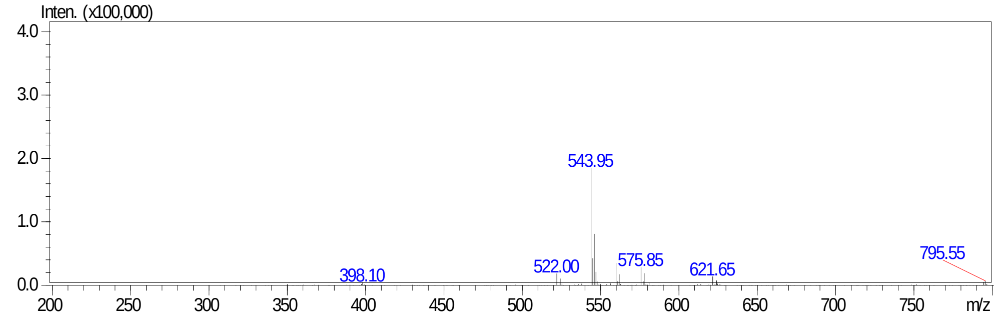

Figure S18. LC/ESI-MS analysis for 6a; ESI-MS, positive mode: *m*/*z* calcd mass for C22H24ClN5NaO4S2 $[M+Na]^+$ = 544.09, was found 543.95.

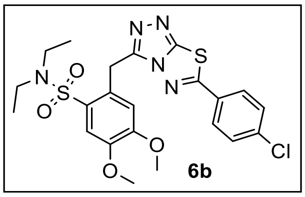

(a)

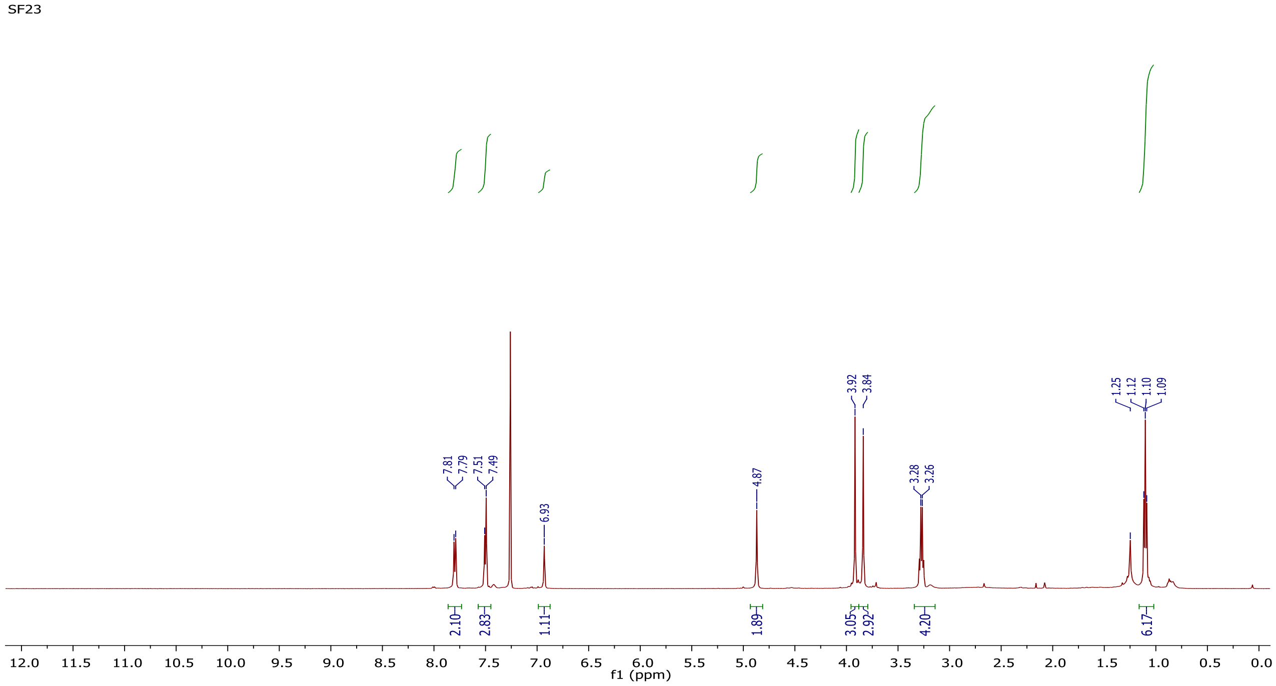

(b)

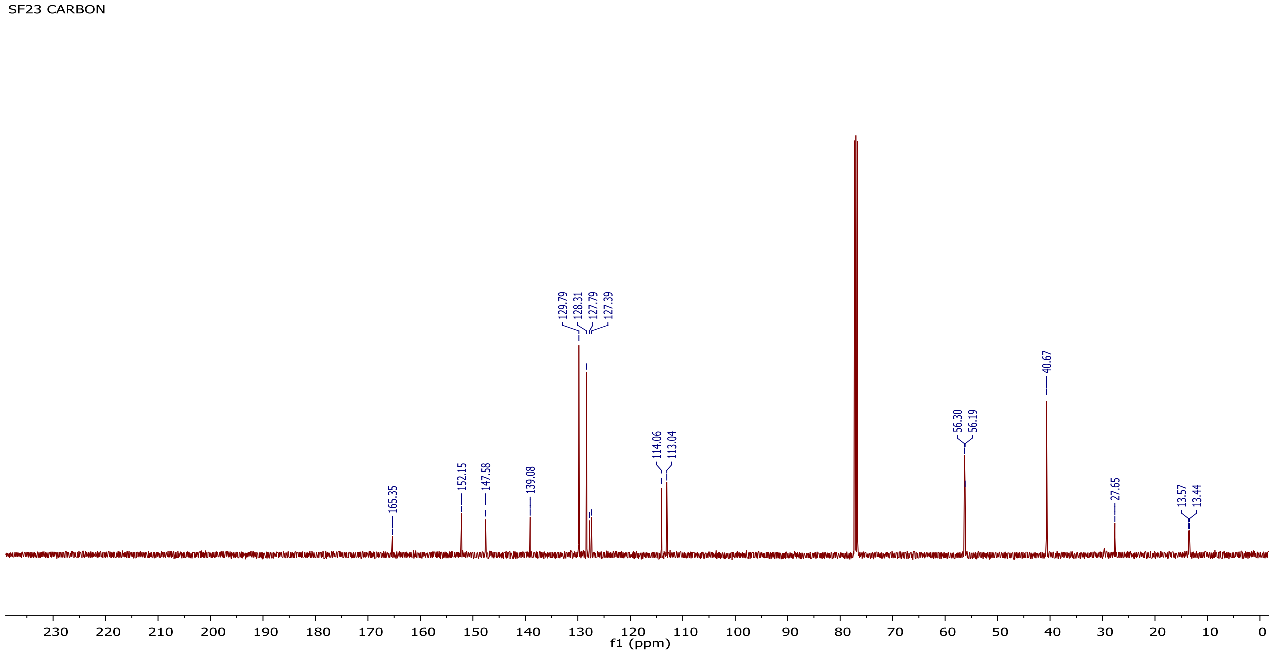

(c)

Figure S19. (**a**) 6b; **(b)** ^{1}H-NMR and (**c**) ^{13}C-NMR spectra for 6b.

Analytical method for LC-MS for 6b

LC-20AD Shimadzu connected to Shimadzu LCMS-2010EV
Mobile Phase: methanol
LC isocratic
HPLC column: SUPELCO Discovery C18, 25cm × 4.6mm, 5µm
Flow rate: 0.4 mL/min
Column temperature: 26 °C
UV detector: 254 nm
MS detector: 1.65 K
Run time: 20 min
Retention time: 10.8 min

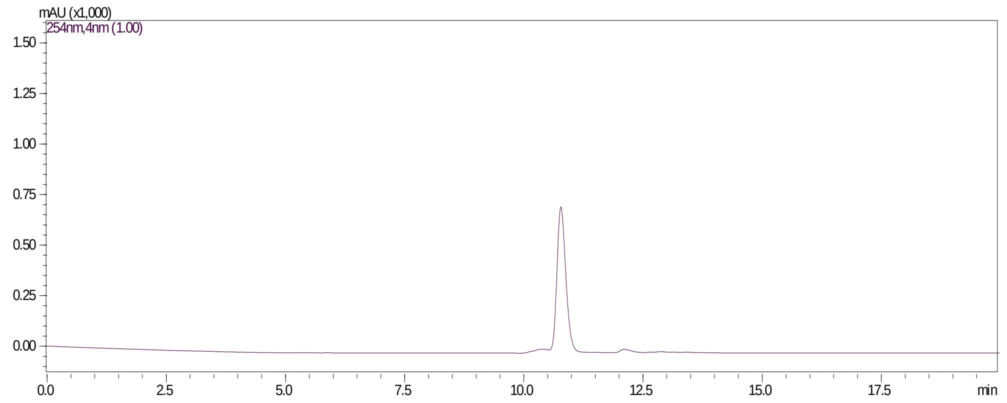

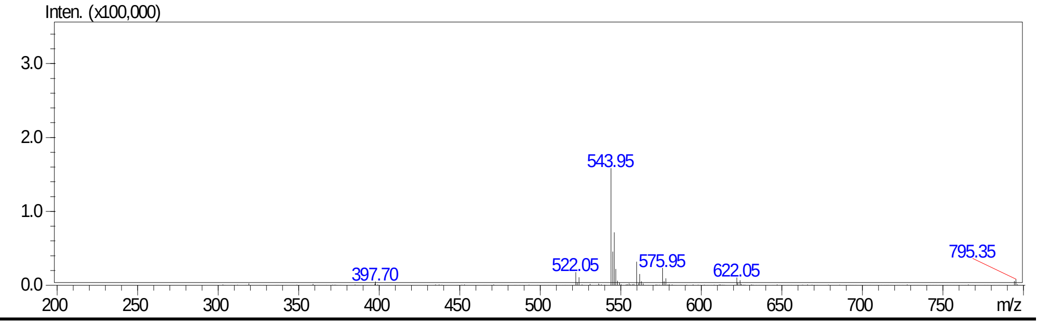

Figure S20. LC/ESI-MS analysis for 6b; ESI-MS, positive mode: *m*/*z* calcd mass for C22H24ClN5NaO4S2 [M+Na]$^+$ = 544.09, was found 543.

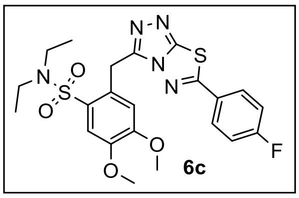

(a)

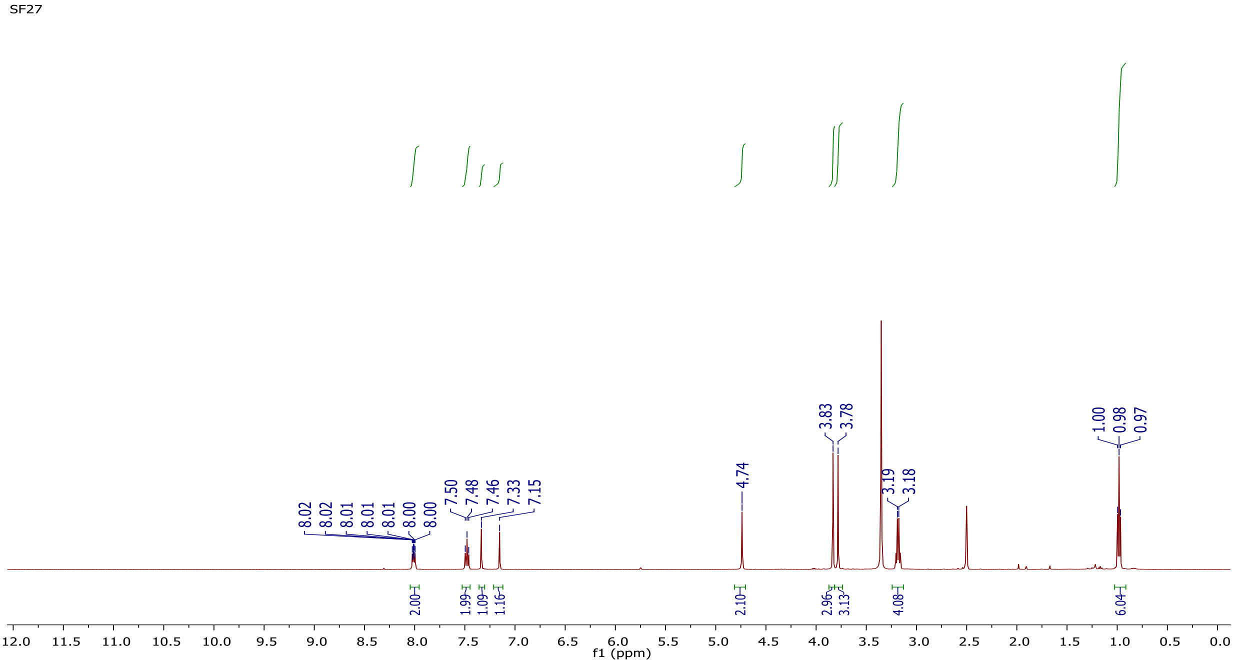

(b)

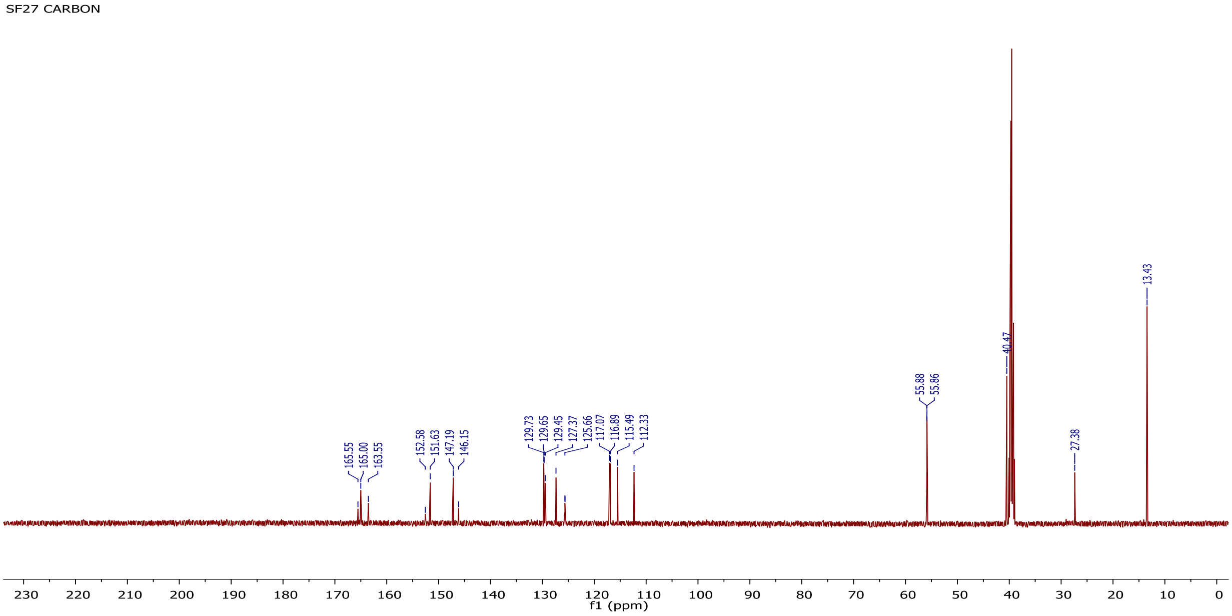

(c)

Figure S21. (**a**) 6c; **(b)** ^{1}H-NMR and (**c**) ^{13}C-NMR spectra for 6c.

Analytical method for LC-MS for 6c

LC-20AD Shimadzu connected to Shimadzu LCMS-2010EV
Mobile Phase: methanol
LC isocratic
HPLC column: SUPELCO Discovery C18, 25cm × 4.6mm, 5µm
Flow rate: 0.4 mL/min
Column temperature: 26 °C
UV detector: 254 nm
MS detector: 1.65 K
Run time: 20 min
Retention time: 11 min

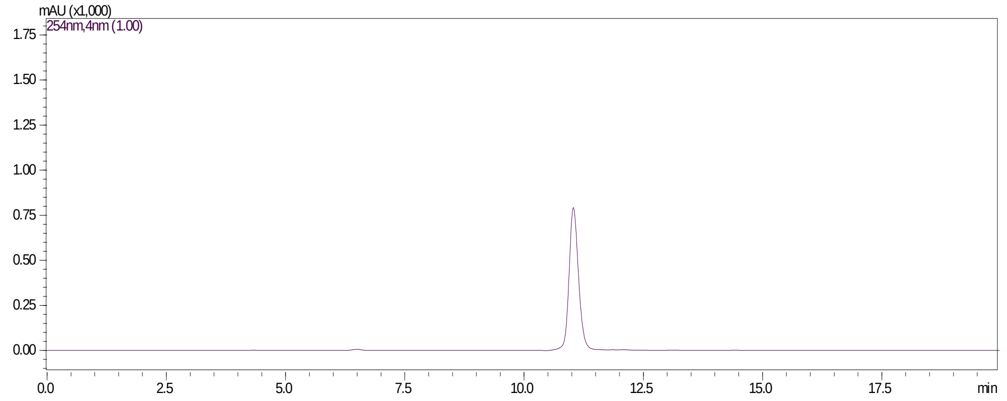

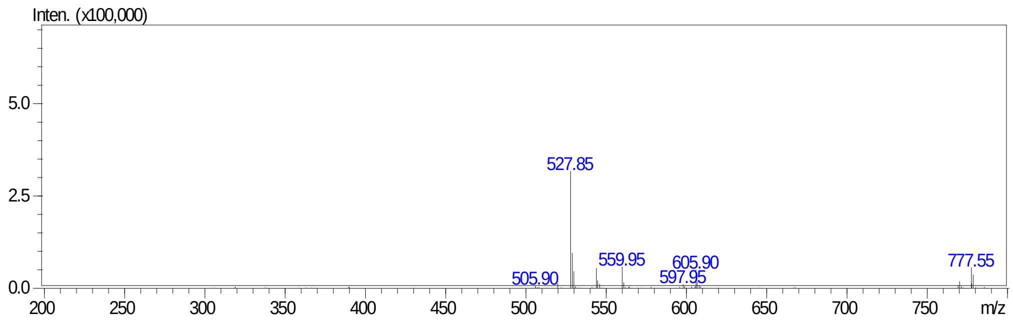

Figure S22. LC/ESI-MS analysis for 6c; ESI-MS, positive mode: *m*/*z* calcd mass for C22H24FN5NaO4S2 $[M+Na]^+$ = 528.12, was found 527.85.

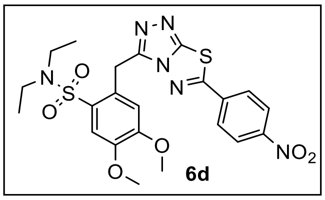

(a)

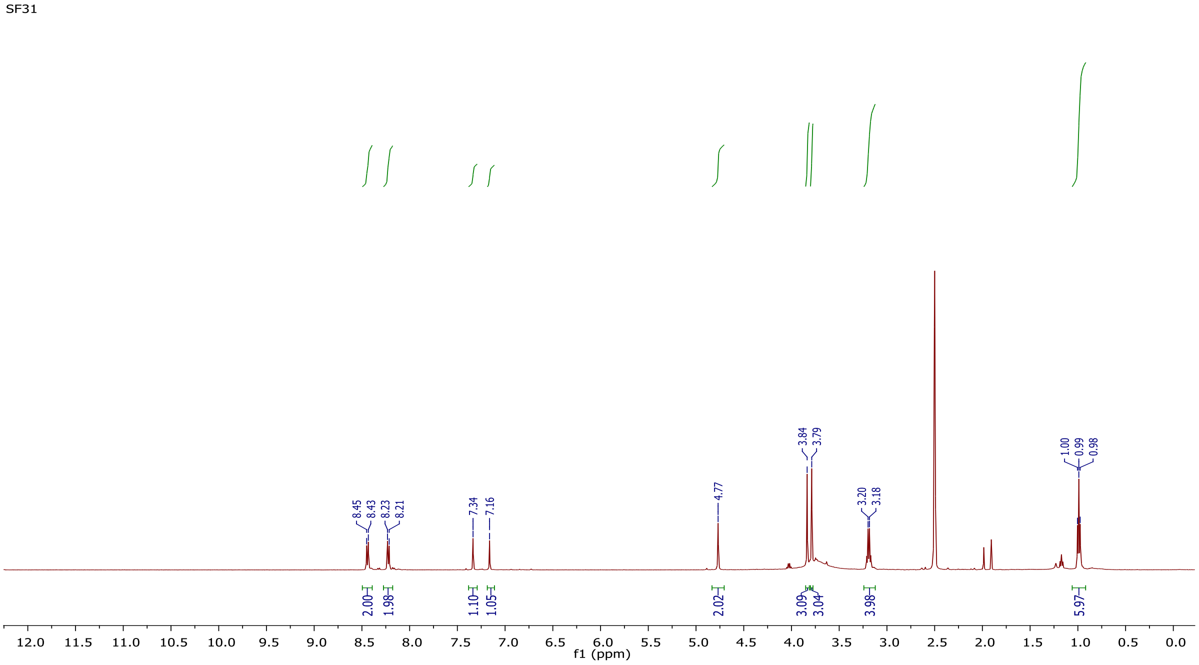

(b)

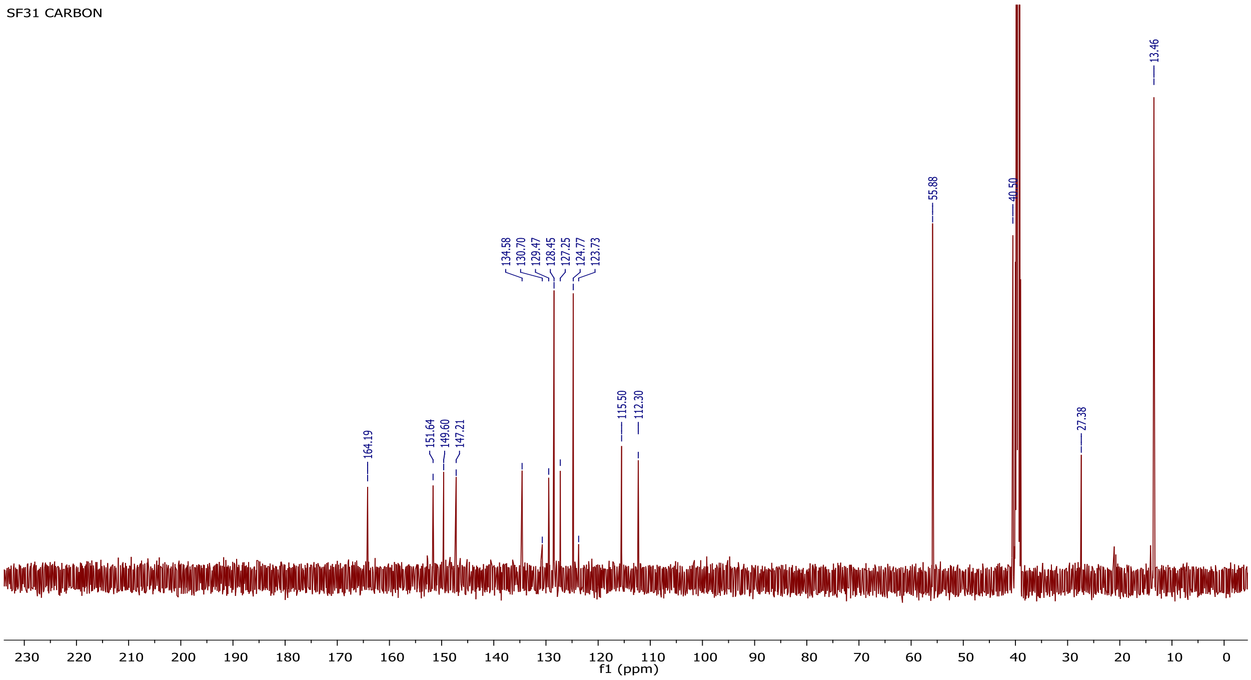

(c)

Figure S23. (**a**) 6b; **(b)** ^{1}H-NMR and (**c**) ^{13}C-NMR spectra for 6d.

Analytical method for LC-MS for 6d

LC-20AD Shimadzu connected to Shimadzu LCMS-2010EV
Mobile Phase: methanol
LC isocratic
HPLC column: SUPELCO Discovery C18, 25cm x 4.6mm, 5µm
Flow rate: 0.4 mL/min
Column temperature: 26 °C
UV detector: 254 nm
MS detector: 1.65 K
Run time: 20 min
Retention time: 12 min

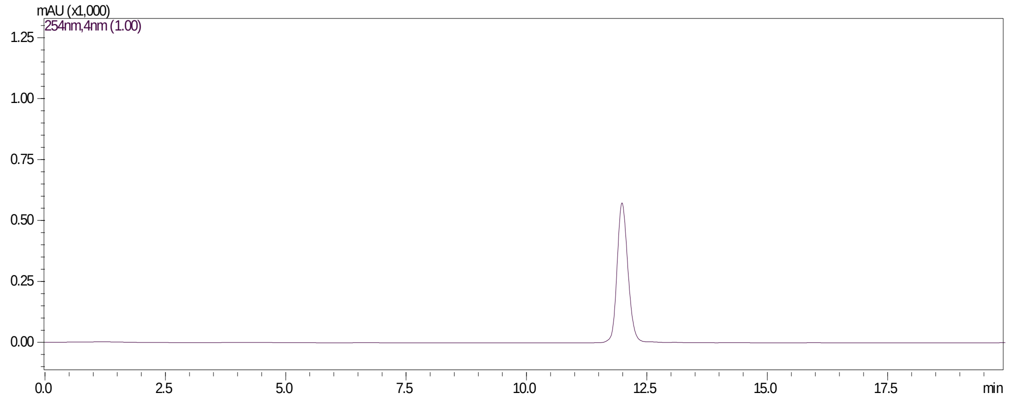

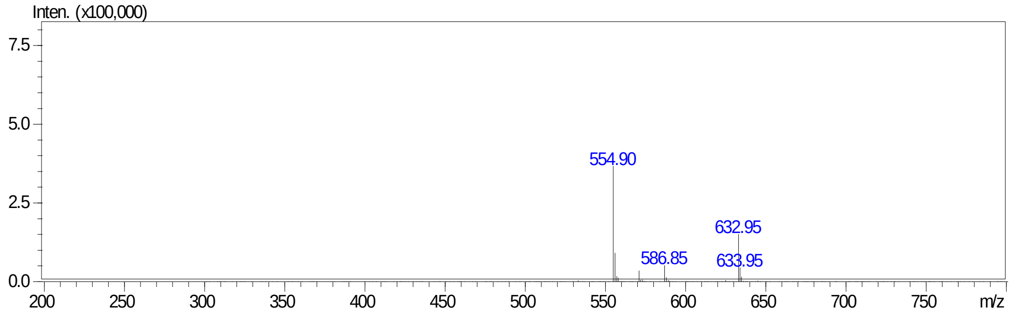

Figure S24. LC/ESI-MS analysis for 6d; ESI-MS, positive mode: *m*/*z* calcd mass for C22H24N6NaO6S2 $[M+Na]^+$ = 555.11, was found 554.90.

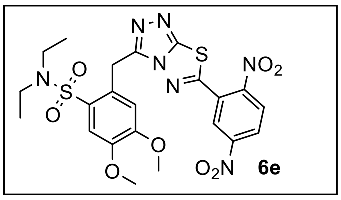

(a)

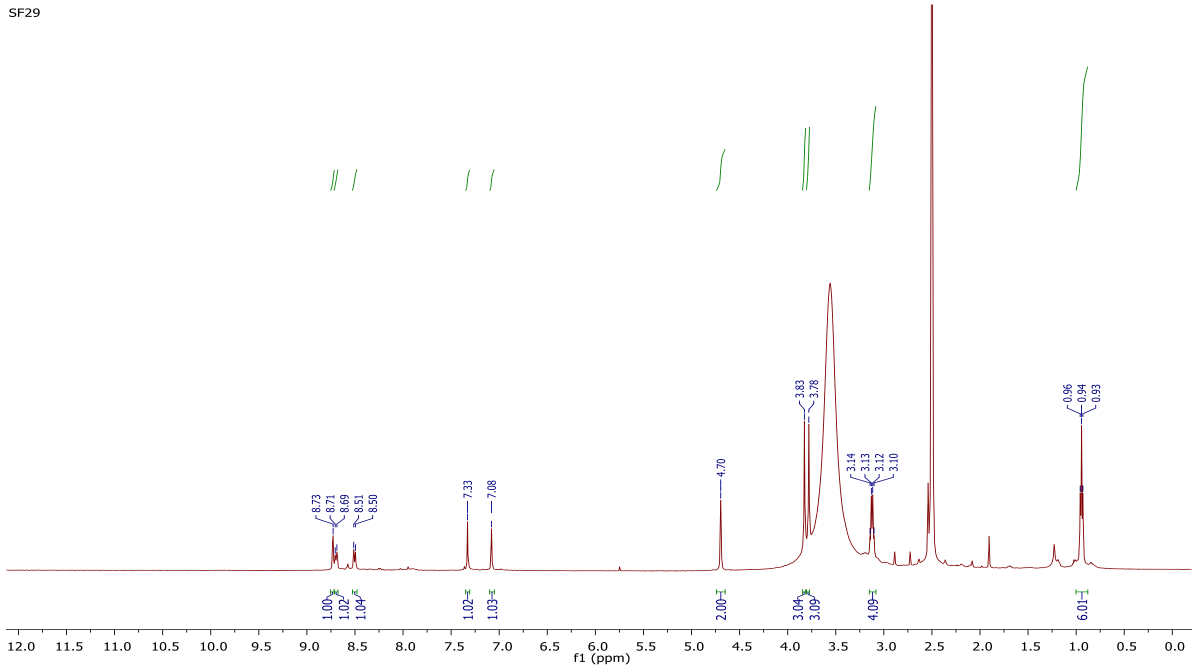

(b)

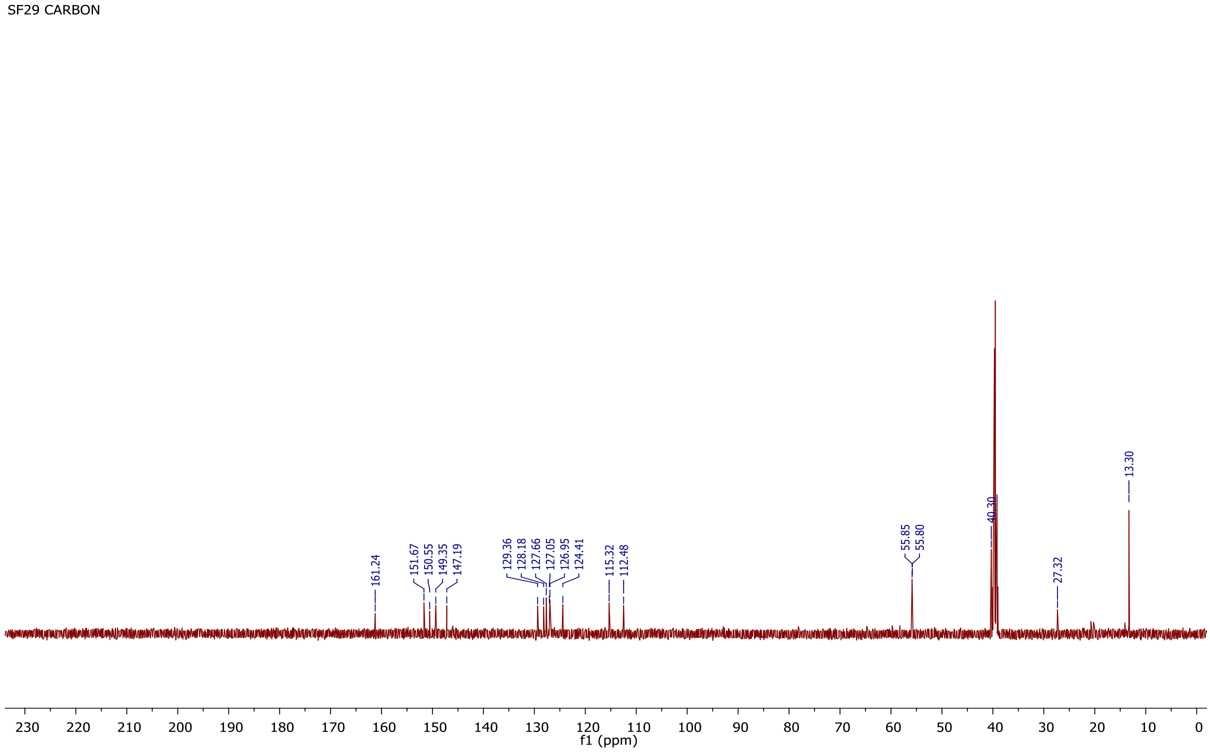

(c)

Figure S25. (**a**) 6e; **(b)** ^{1}H-NMR and (**c**) ^{13}C-NMR spectra for 6e.

Analytical method for LC-MS for 6e

LC-20AD Shimadzu connected to Shimadzu LCMS-2010EV
Mobile Phase: methanol
LC isocratic
HPLC column: SUPELCO Discovery C18, 25cm × 4.6mm, 5µm
Flow rate: 0.4 mL/min
Column temperature: 26 °C
UV detector: 254 nm
MS detector: 1.65 K
Run time: 20 min
Retention time: 9.2 min

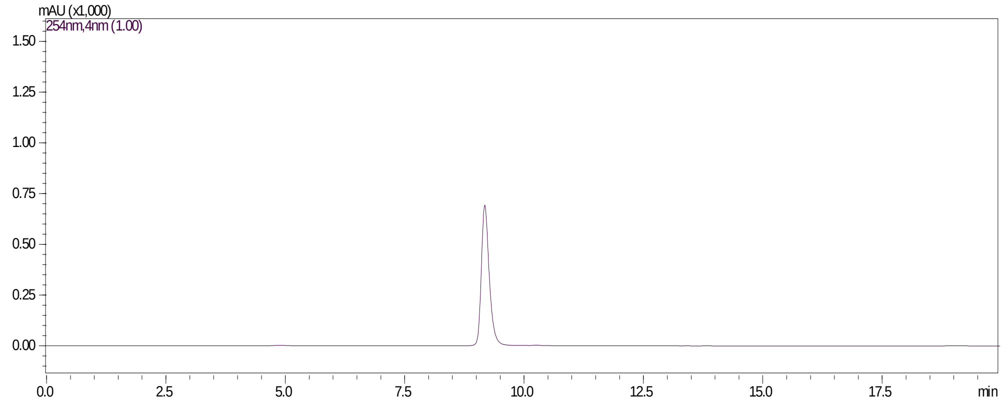

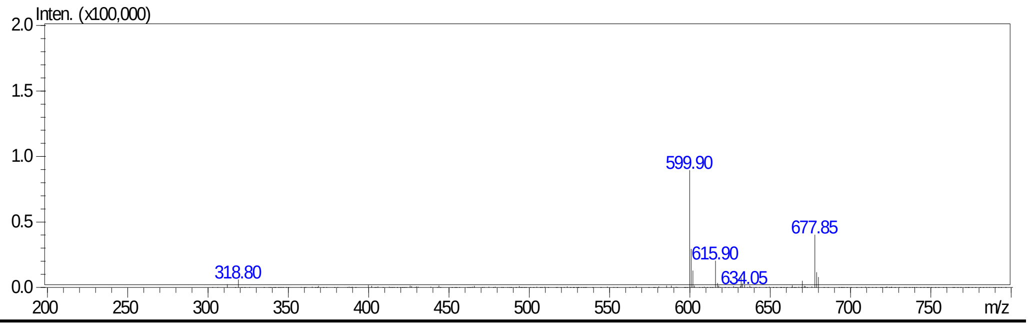

Figure S26. LC/ESI-MS analysis for 6e; ESI-MS, positive mode: *m*/*z* calcd mass for C22H23N7NaO8S2 $[M+Na]^+$ = 600.09, was found 599.90.

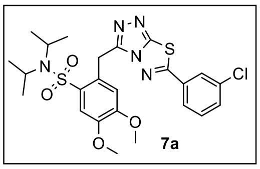

(a)

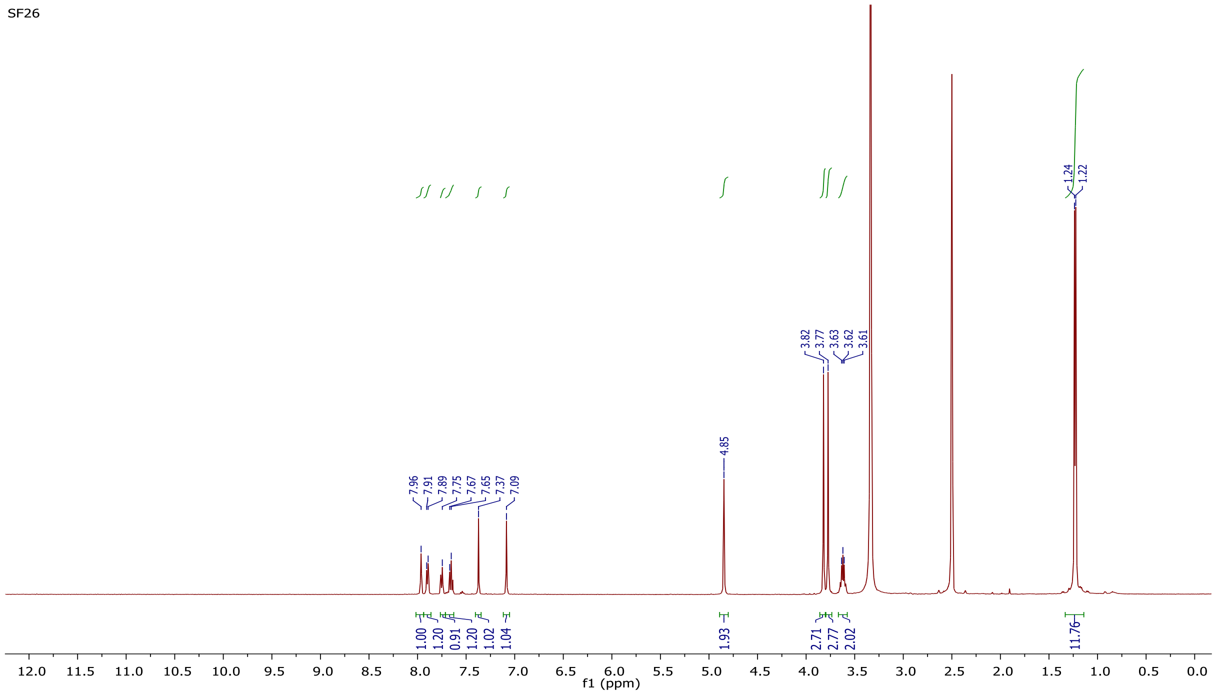

(b)

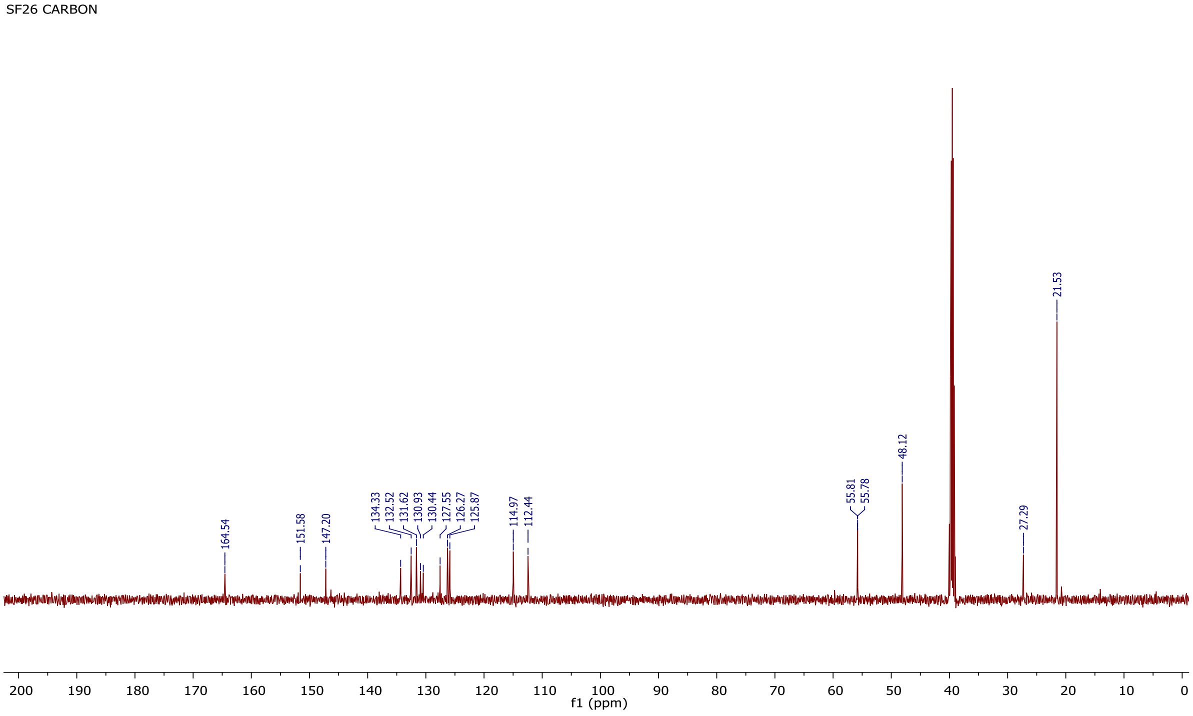

(c)

Figure S27. (**a**) 7a; **(b)** ^{1}H-NMR and (**c**) ^{13}C-NMR spectra for 7a.

Analytical method for LC-MS for 7a

LC-20AD Shimadzu connected to Shimadzu LCMS-2010EV
Mobile Phase: methanol
LC isocratic
HPLC column: SUPELCO Discovery C18, 25cm × 4.6mm, 5µm
Flow rate: 0.4 mL/min
Column temperature: 26 °C
UV detector: 254 nm
MS detector: 1.65 K
Run time: 20 min
Retention time: 11.7 min

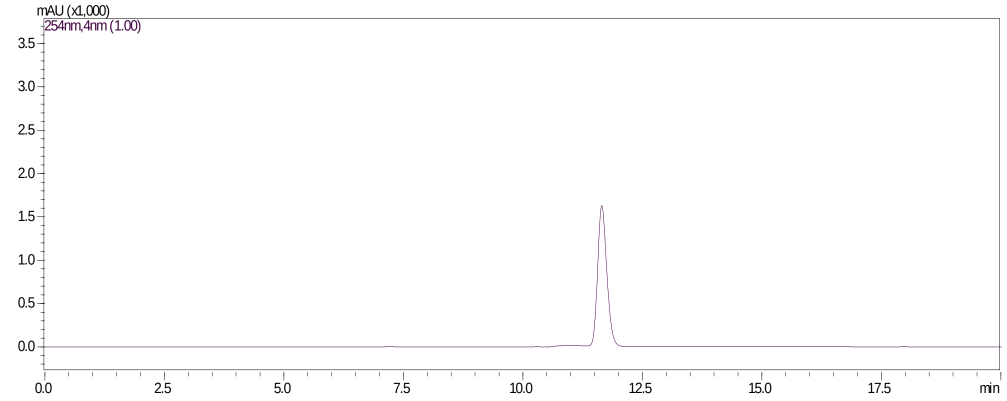

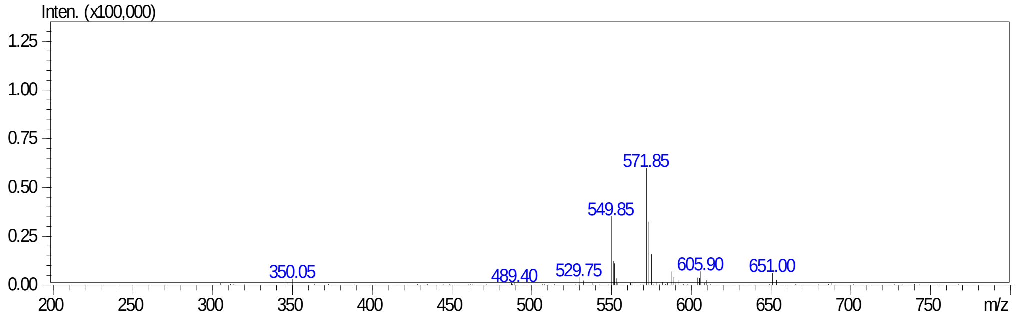

Figure S28. LC/ESI-MS analysis for 7a; ESI-MS, positive mode: *m*/*z* calcd mass for C24H29ClN5O4S2 $[M+H]^+$ = 550.13, was found 549.85.

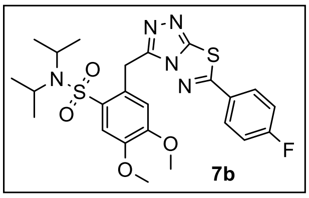

(a)

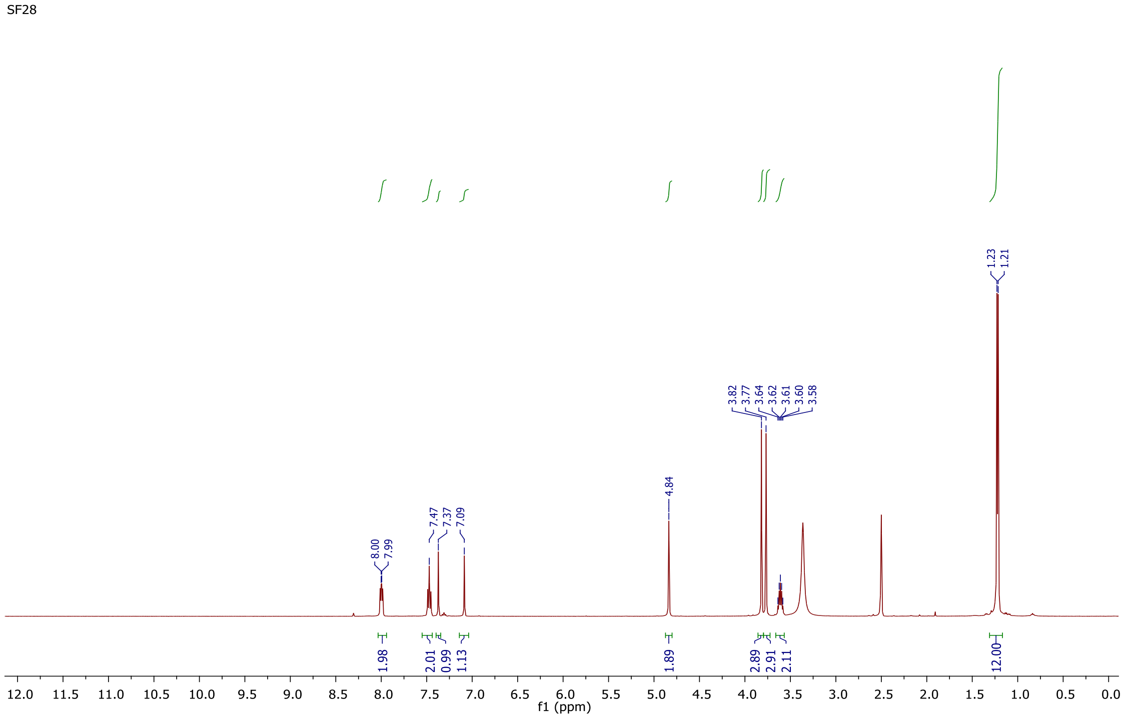

(b)

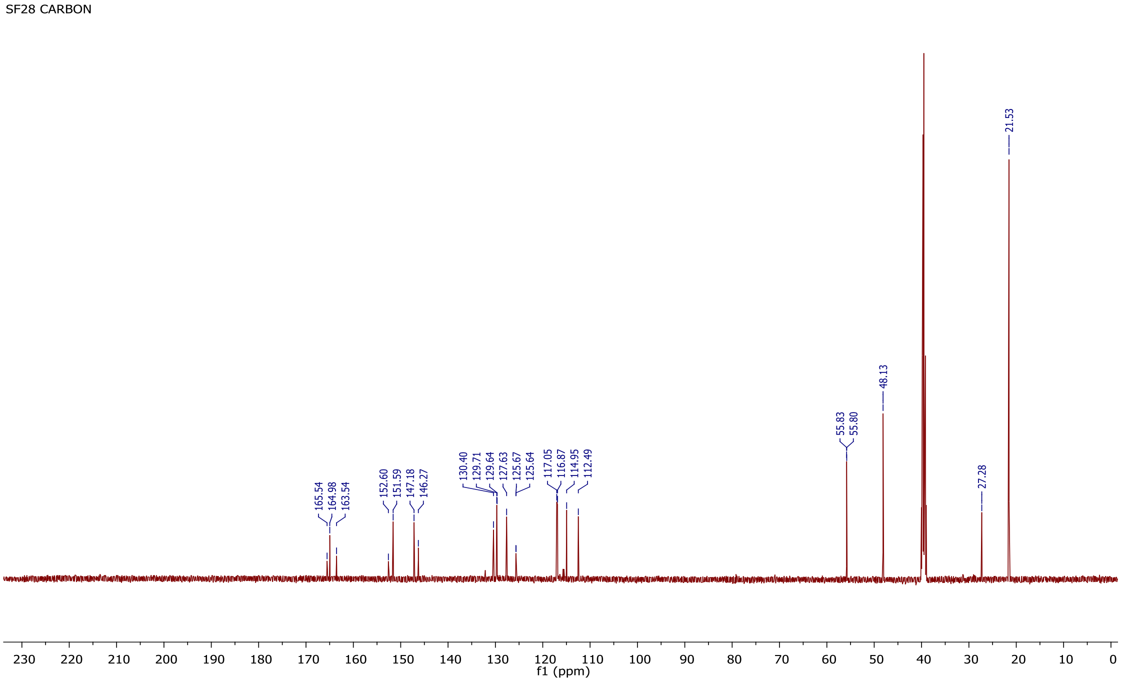

(c)

Figure S29. (**a**) 7b; **(b)** ^{1}H-NMR and (**c**) ^{13}C-NMR spectra for 7b.

Analytical method for LC-MS for 7b

LC-20AD Shimadzu connected to Shimadzu LCMS-2010EV
Mobile Phase: methanol
LC isocratic
HPLC column: SUPELCO Discovery C18, 25cm × 4.6mm, 5μm
Flow rate: 0.4 mL/min
Column temperature: 26 °C
UV detector: 254 nm
MS detector: 1.65 K
Run time: 20 min
Retention time: 9.2 min

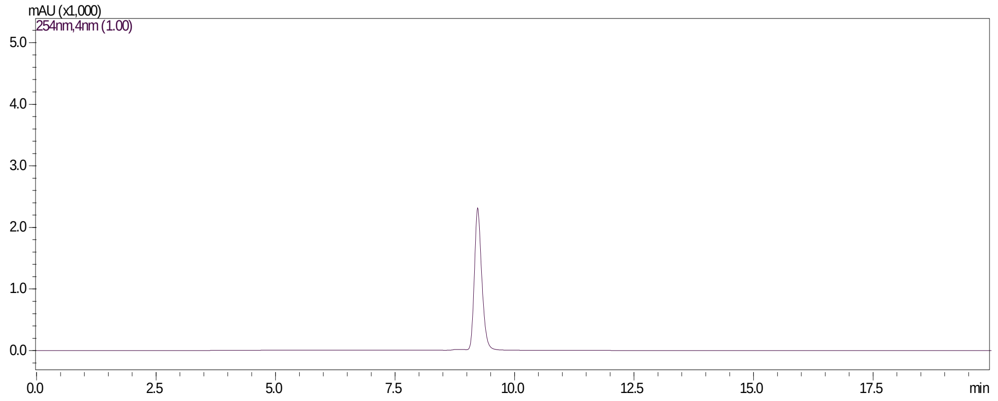

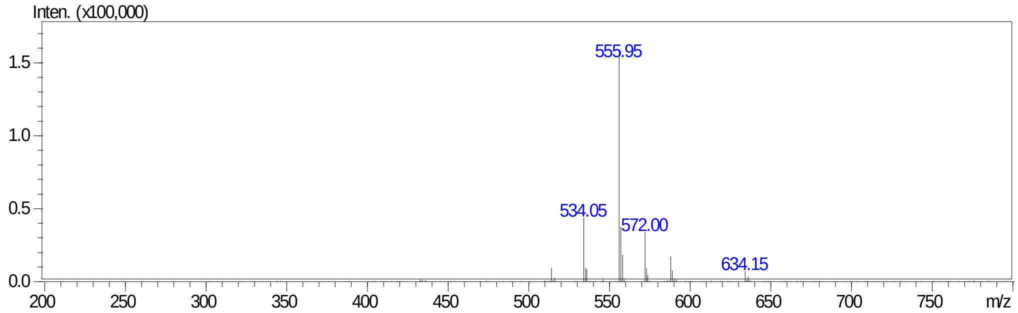

Figure S30. LC/ESI-MS analysis for 7b; ESI-MS, positive mode: *m*/*z* calcd mass for C24H28FN5NaO4S2 $[M+Na]^+$ = 556.15, was found 555.95.

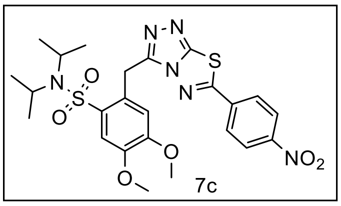

(a)

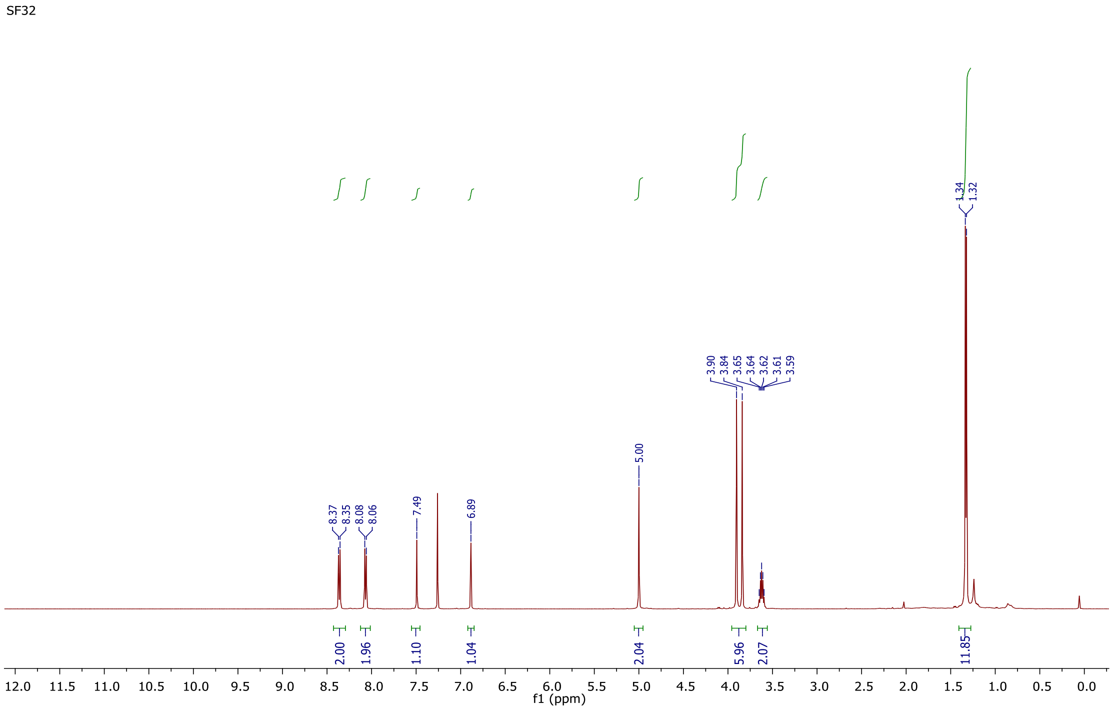

(b)

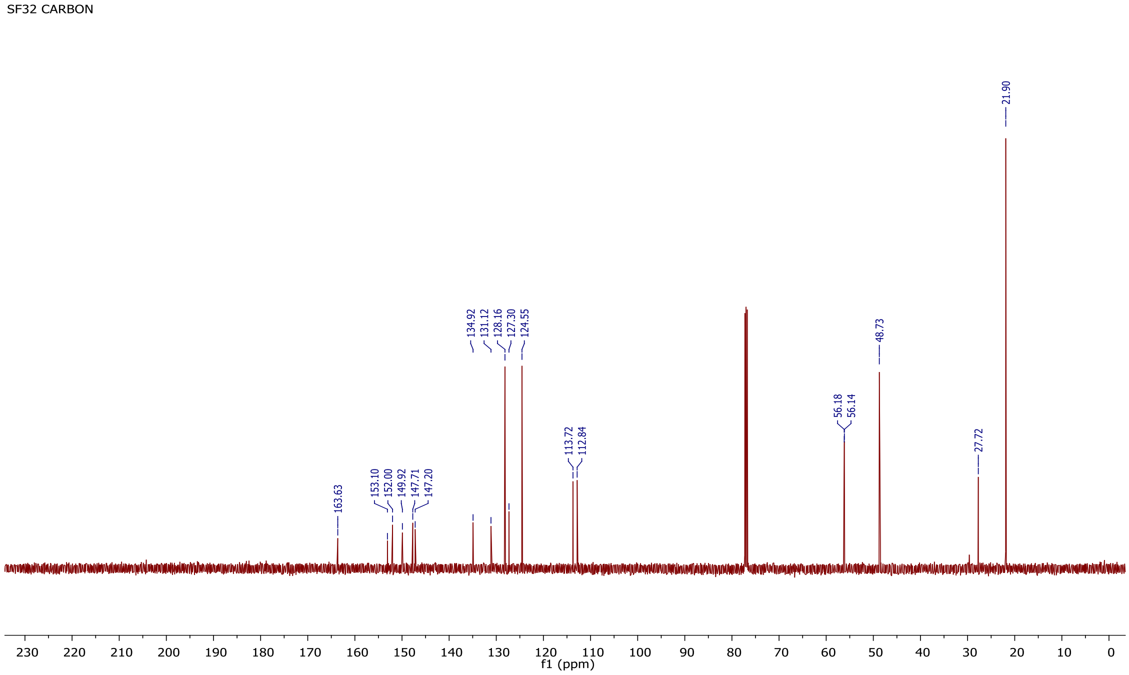

(c)

Figure S31. (a) 7c; **(b)** ^{1}H-NMR and **(c)** ^{13}C-NMR spectra for 7c.

Analytical method for LC-MS foe 7c

LC-20AD Shimadzu connected to Shimadzu LCMS-2010EV
Mobile Phase: methanol
LC isocratic
HPLC column: SUPELCO Discovery C18, 25cm × 4.6mm, 5μm
Flow rate: 0.4 mL/min
Column temperature: 26 °C
UV detector: 254 nm
MS detector: 1.65 K
Run time: 20 min
Retention time: 9.8 min

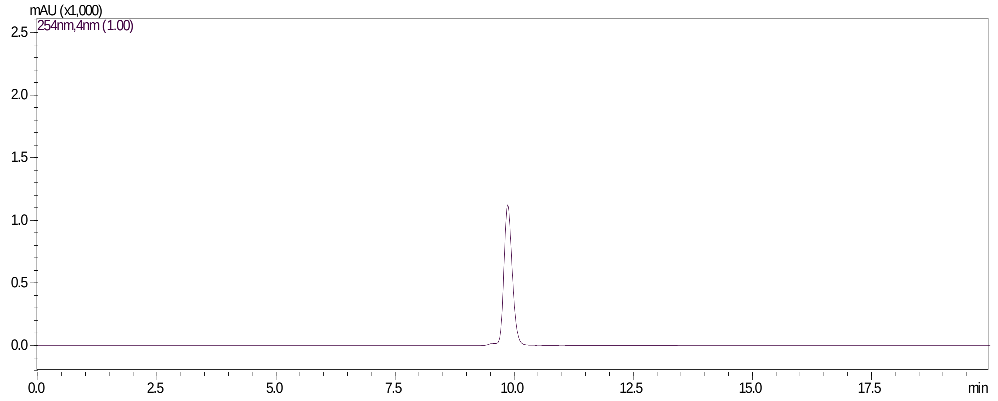

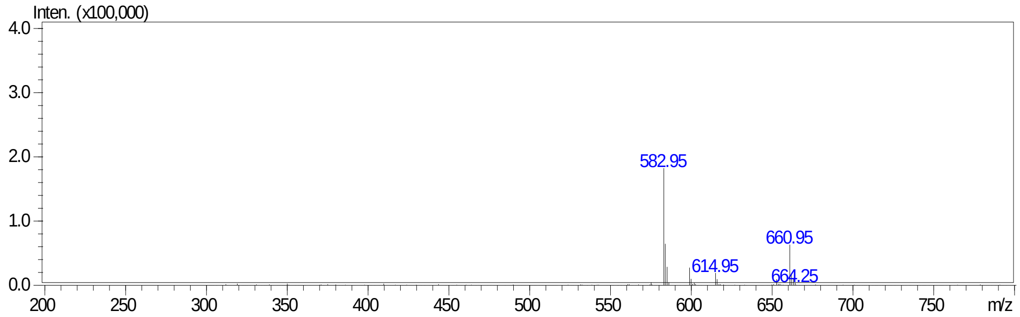

Figure S32. LC/ESI-MS analysis for 7c; ESI-MS, positive mode: *m*/*z* calcd mass for C24H28N6NaO6S2 $[M+Na]^+$ = 583.14, was found 582.95.

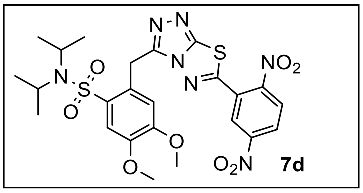

(a)

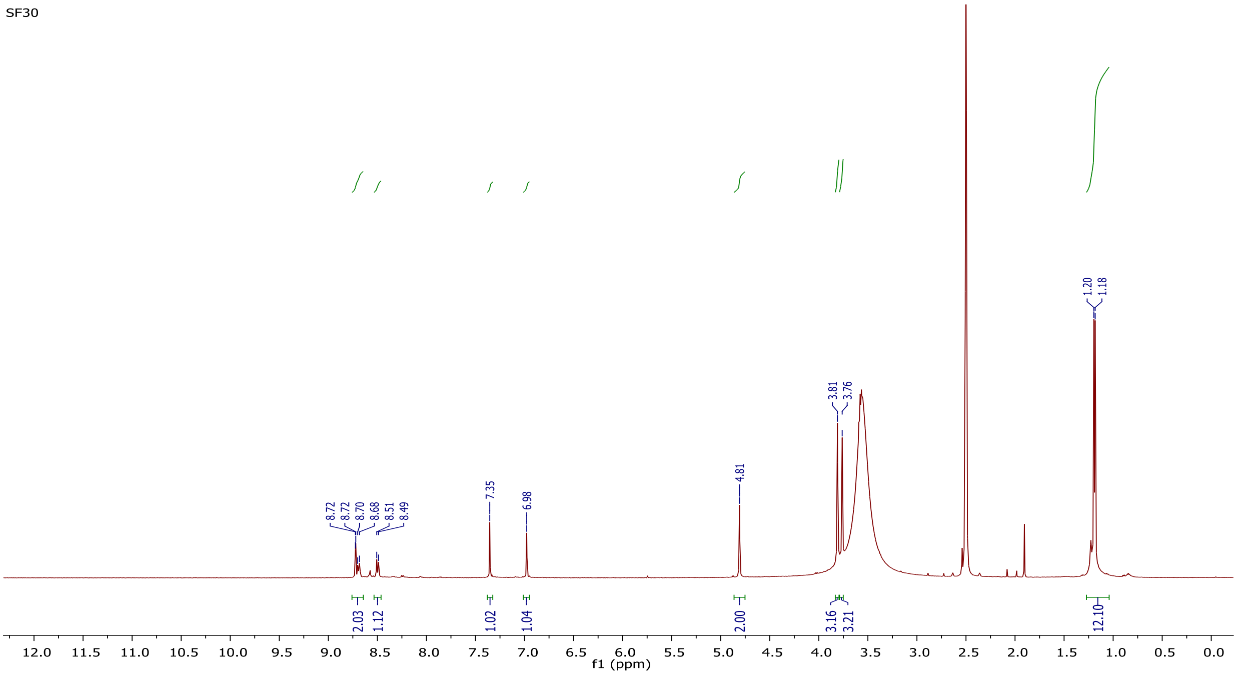

(b)

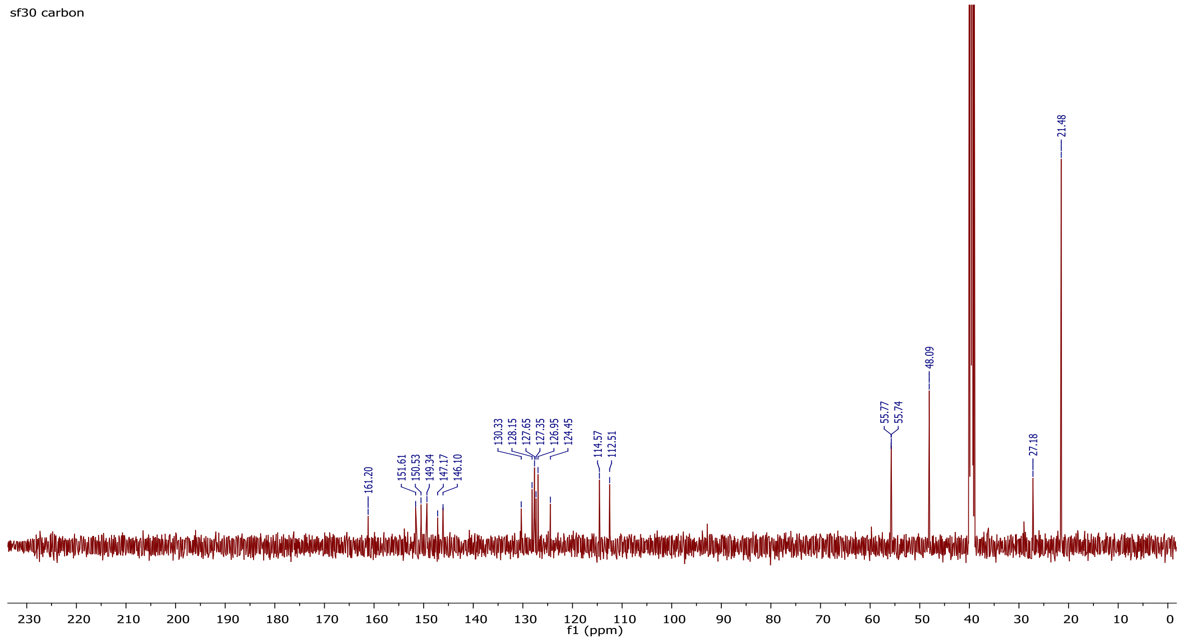

(c)

Figure S33. (**a**) 7d; **(b)** ^{1}H-NMR and (**c**) ^{13}C-NMR spectra for 7d.

Analytical method for LC-MS for 7d

LC-20AD Shimadzu connected to Shimadzu LCMS-2010EV
Mobile Phase: methanol
LC isocratic
HPLC column: SUPELCO Discovery C18, 25cm × 4.6mm, 5μm
Flow rate: 0.4 mL/min
Column temperature: 26 °C
UV detector: 254 nm
MS detector: 1.65 K
Run time: 11 min
Retention time: 7.9 min

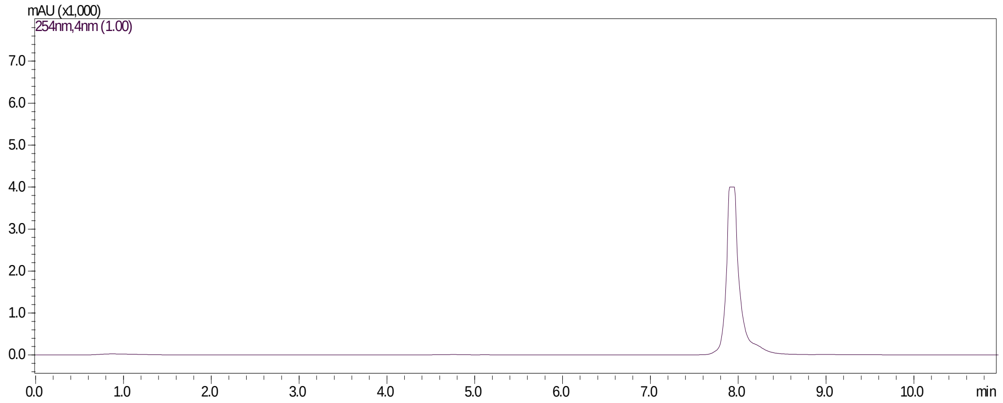

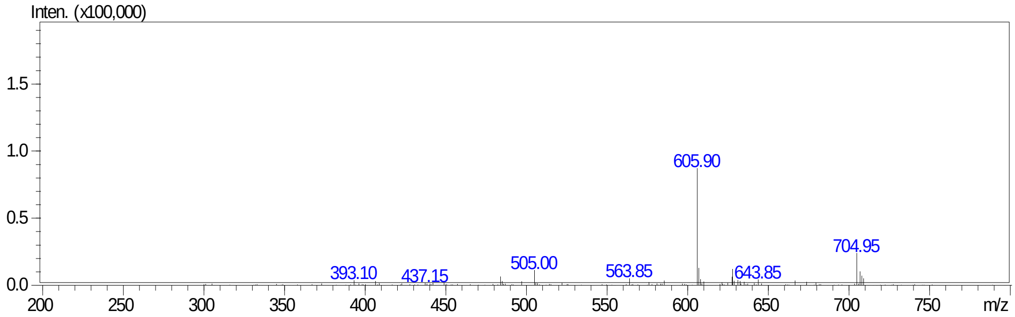

Figure S34. LC/ESI-MS analysis for 7d; ESI-MS, positive mode: *m*/*z* calcd mass for C24H27KN7O8S2 $[M+H]^+$ = 606.14, was found 605.90.

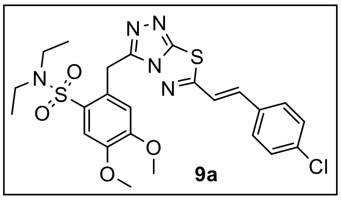

(a)

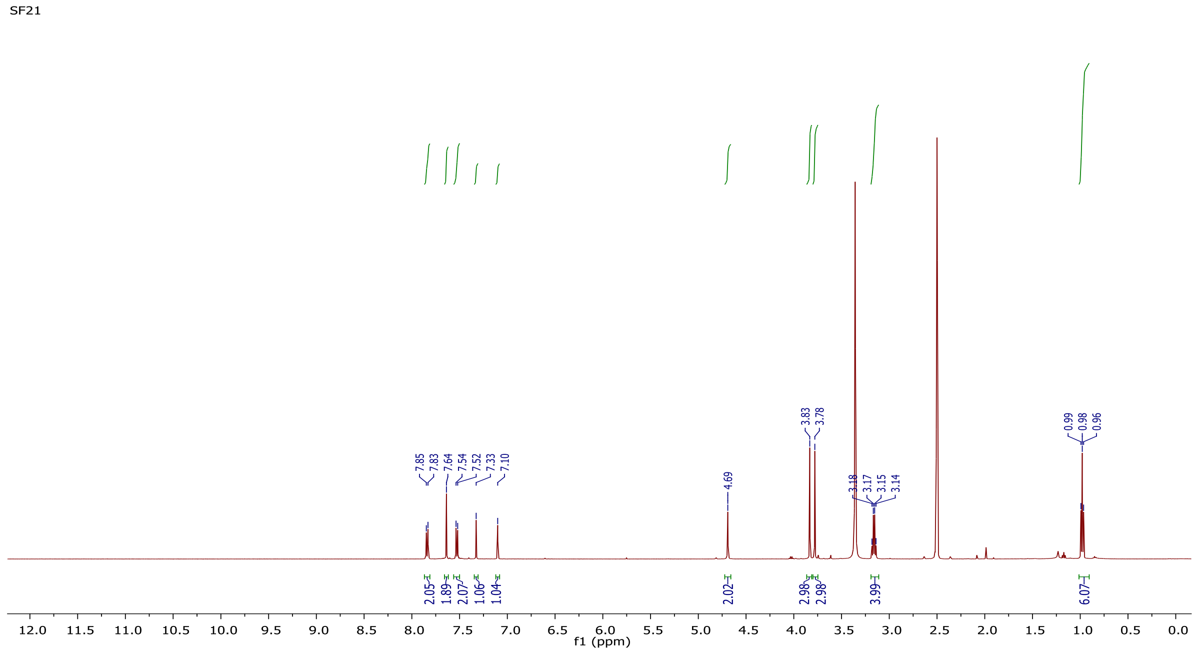

(b)

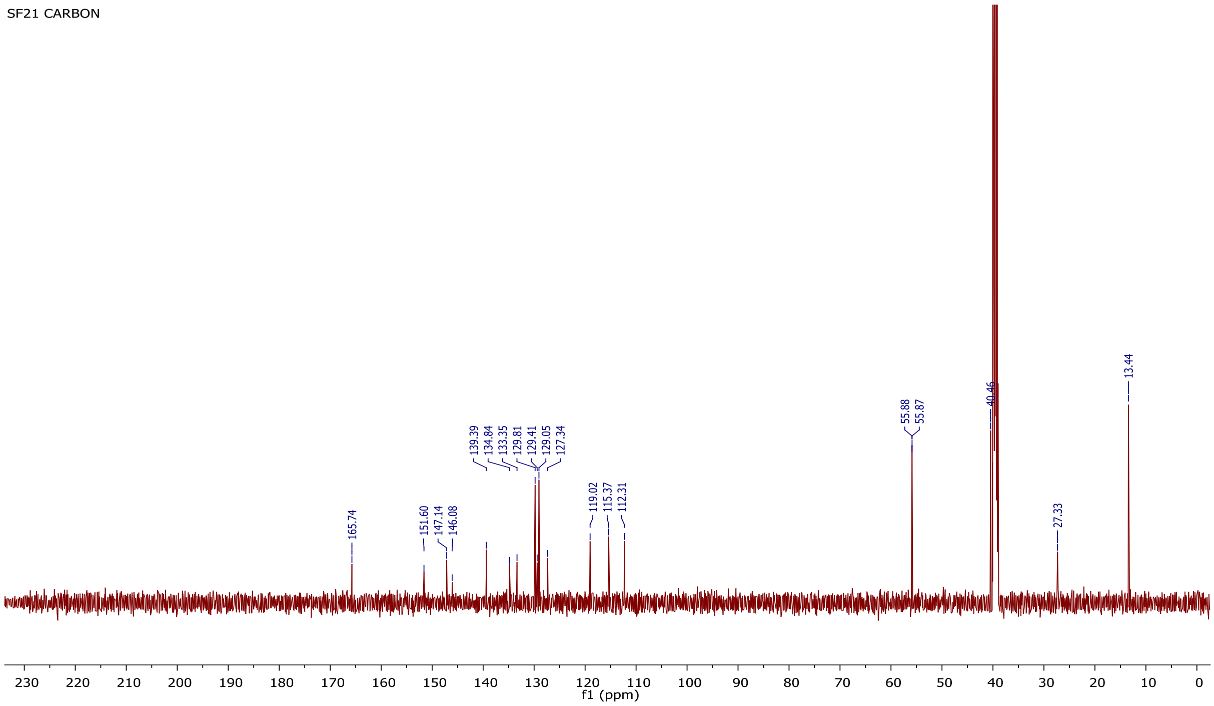

(c)

Figure S35. (**a**) 9a; **(b)** ^{1}H-NMR and (**c**) ^{13}C-NMR spectra for 9a.

Analytical method for LC-MS for 9a

LC-20AD Shimadzu connected to Shimadzu LCMS-2010EV
Mobile Phase: methanol
LC isocratic
HPLC column: SUPELCO Discovery C18, 25cm × 4.6mm, 5µm
Flow rate: 0.4 mL/min
Column temperature: 26 °C
UV detector: 254 nm
MS detector: 1.65 K
Run time: 20 min
Retention time: 10.8 min

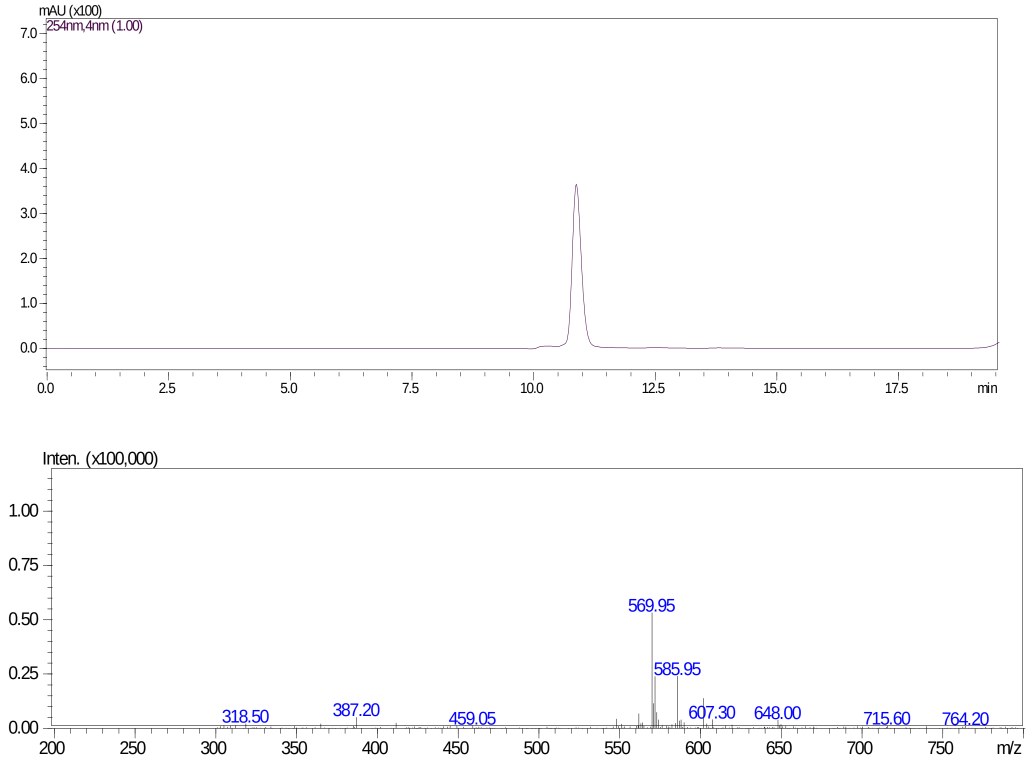

Figure S36. LC/ESI-MS analysis for 9a; ESI-MS, positive mode: *m*/*z* calcd mass for C24H26ClN5NaO4S2 $[M+Na]^+$ = 570.10, was found 569.95.

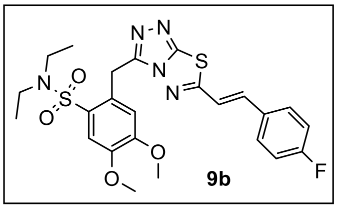

(a)

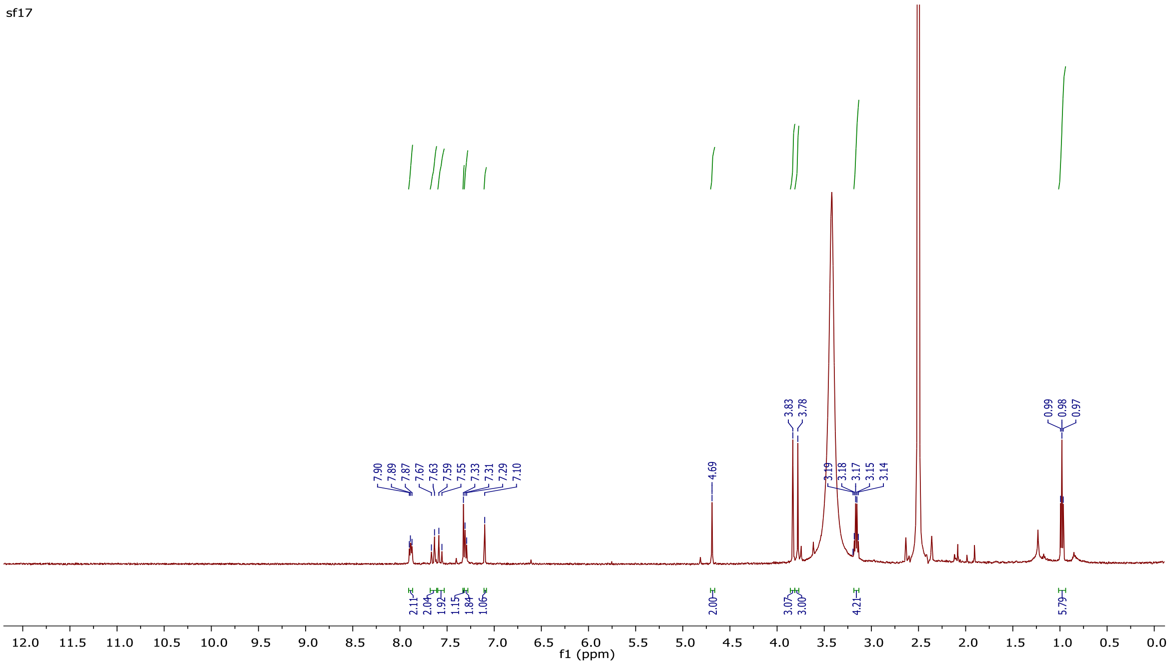

(b)

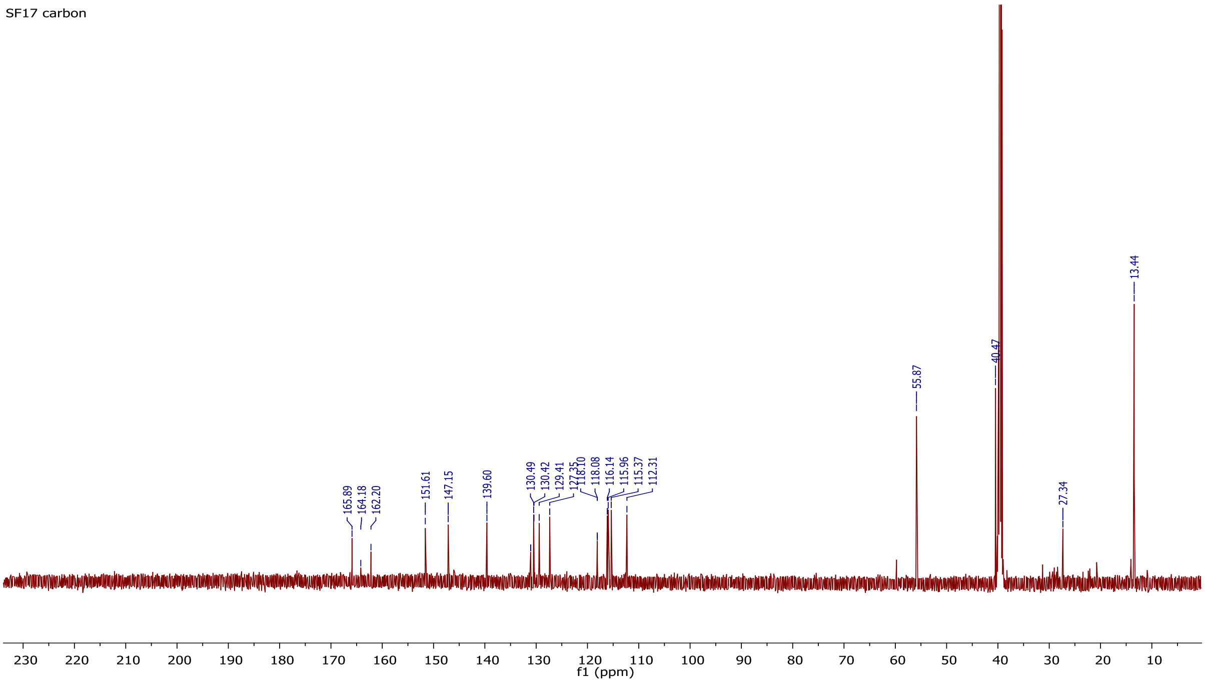

(c)

Figure S37. (**a**) 9b; **(b)** ^{1}H-NMR and (**c**) ^{13}C-NMR spectra for 9b.

Analytical method for LC-MS for 9b

LC-20AD Shimadzu connected to Shimadzu LCMS-2010EV
Mobile Phase: methanol
LC isocratic
HPLC column: SUPELCO Discovery C18, 25cm × 4.6mm, 5μm
Flow rate: 0.4 mL/min
Column temperature: 26 °C
UV detector: 254 nm
MS detector: 1.65 K
Run time: 11 min
Retention time: 8.4 min

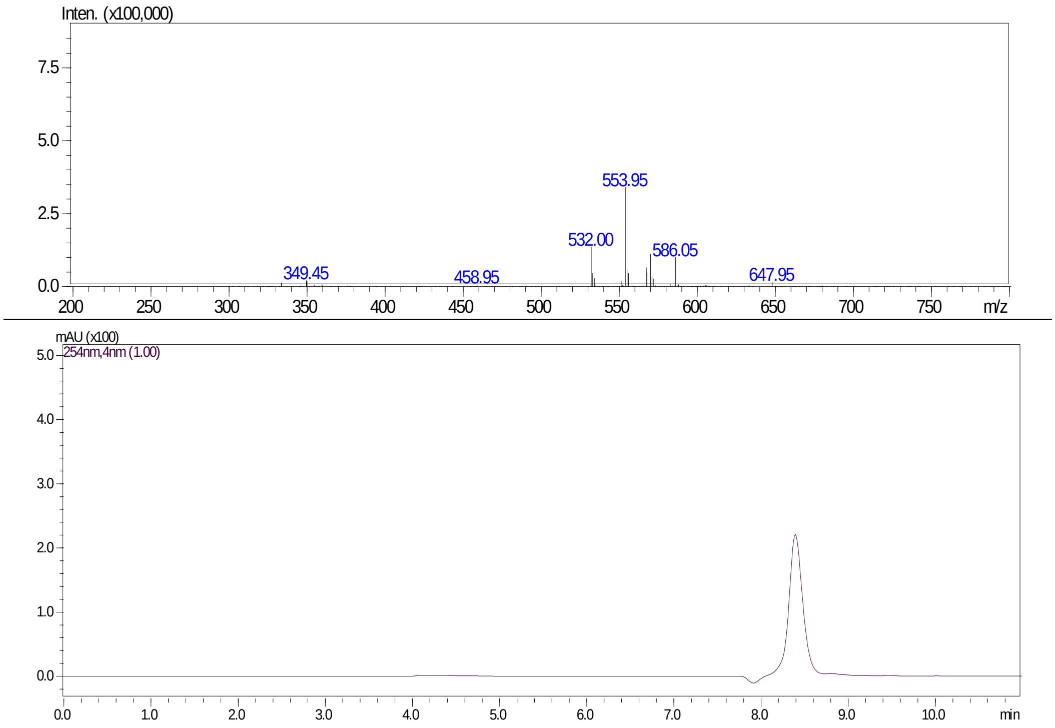

Figure S38. LC/ESI-MS analysis for 9b; ESI-MS, positive mode: *m*/*z* calcd mass for C24H27FN5O4S2 $[M+H]^+$ = 532.15, was found 532.00.

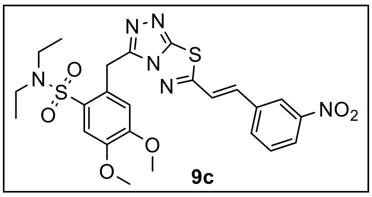

(a)

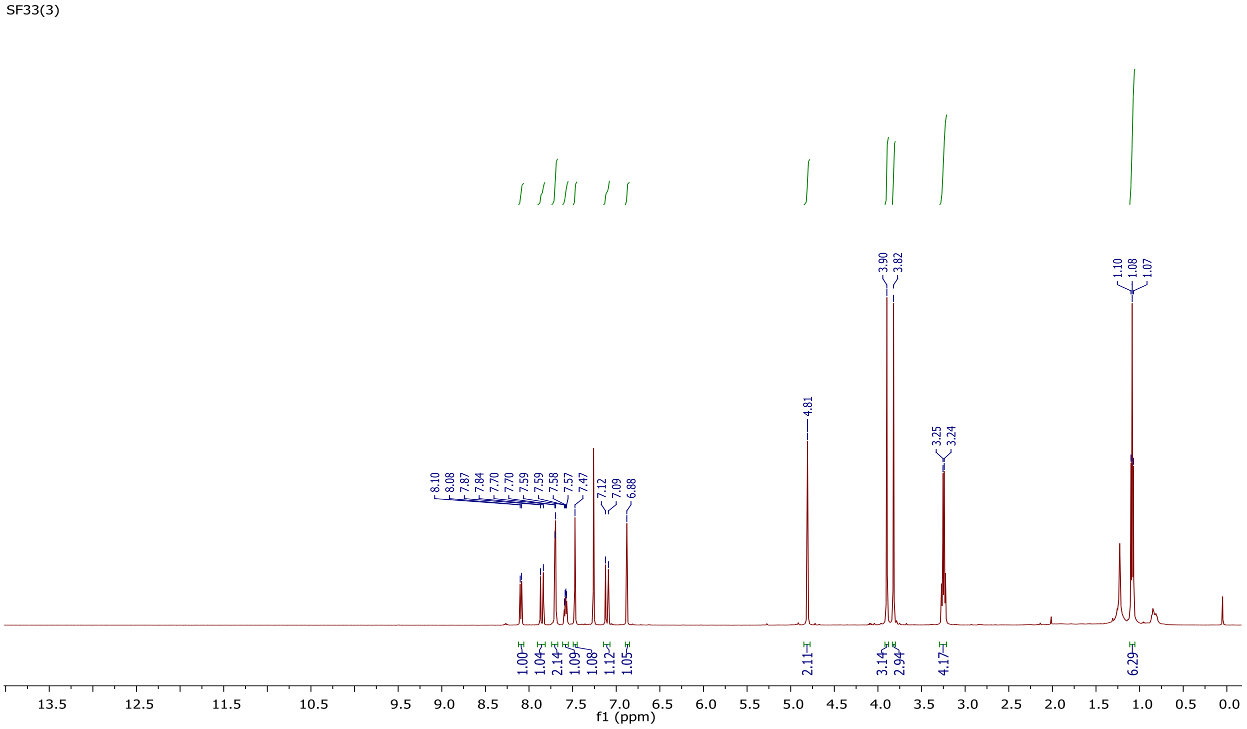

(b)

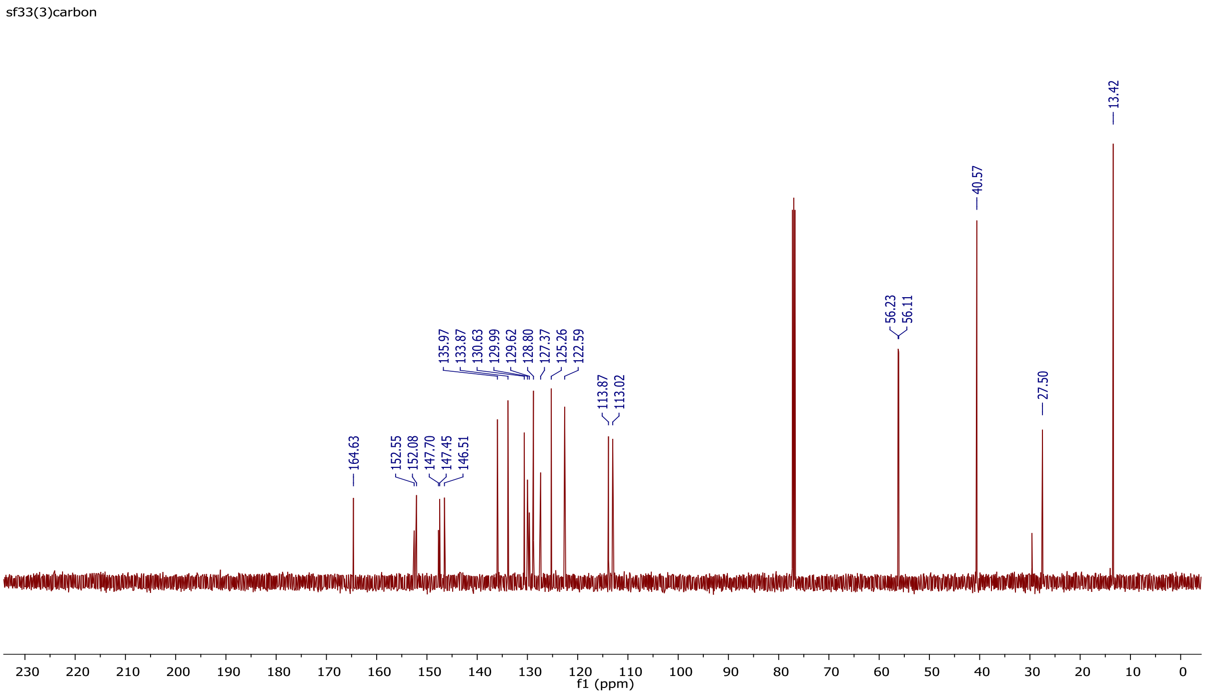

(c)

Figure S39. (**a**) 9c; **(b)** ^{1}H-NMR and (**c**) ^{13}C-NMR spectra for 9c.

Analytical method for LC-MS for 9c

LC-20AD Shimadzu connected to Shimadzu LCMS-2010EV
Mobile Phase: methanol
LC isocratic
HPLC column: SUPELCO Discovery C18, 25cm × 4.6mm, 5µm
Flow rate: 0.4 mL/min
Column temperature: 26 °C
UV detector: 254 nm
MS detector: 1.65 K
Run time: 15 min
Retention time: 9.1 min

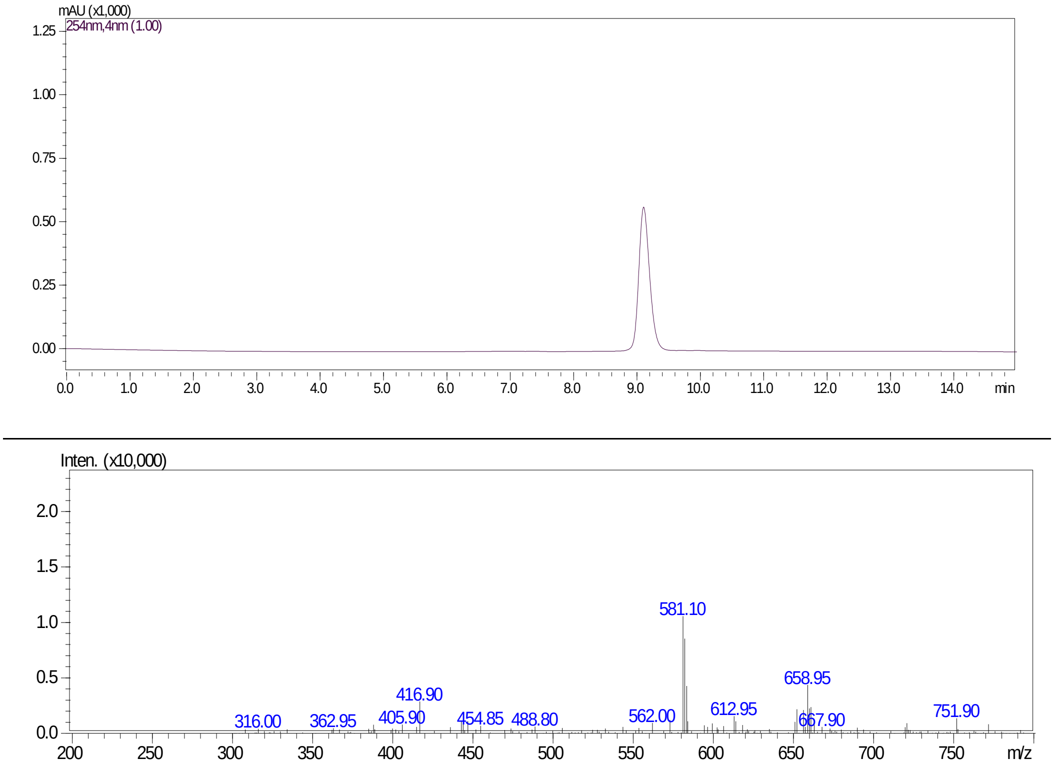

Figure S40. LC/ESI-MS analysis for 9c; ESI-MS, positive mode: *m*/*z* calcd mass for C24H26N6NaO6S2 $[M+Na]^+$ = 581.13, was found 581.10.

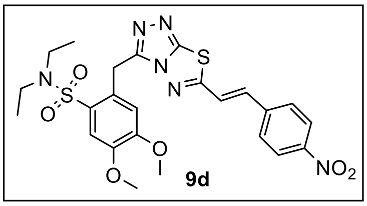

(a)

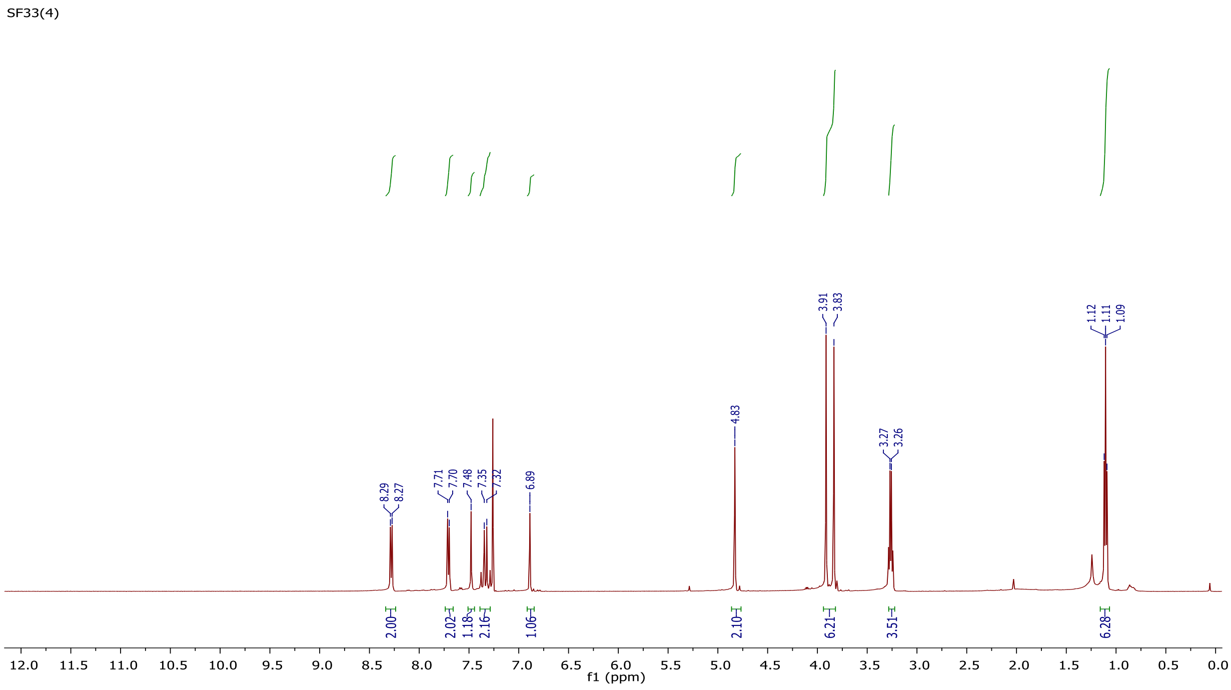

(b)

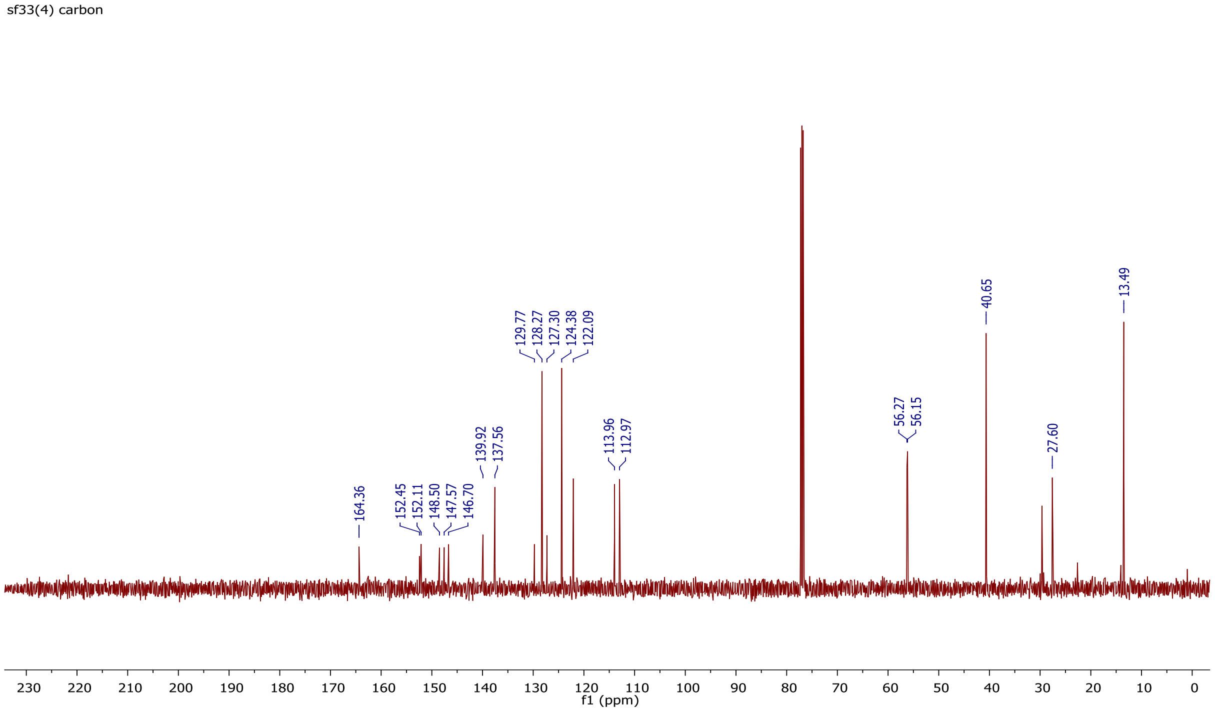

(c)

Figure S41. (**a**) 9d; **(b)** ^{1}H-NMR and (**c**) ^{13}C-NMR spectra for 9d.

Analytical method for LC-MS for 9d

LC-20AD Shimadzu connected to Shimadzu LCMS-2010EV
Mobile Phase: methanol
LC isocratic
HPLC column: SUPELCO Discovery C18, 25cm × 4.6mm, 5µm
Flow rate: 0.4 mL/min
Column temperature: 26 °C
UV detector: 254 nm
MS detector: 1.65 K
Run time: 20 min
Retention time: 11.7 min

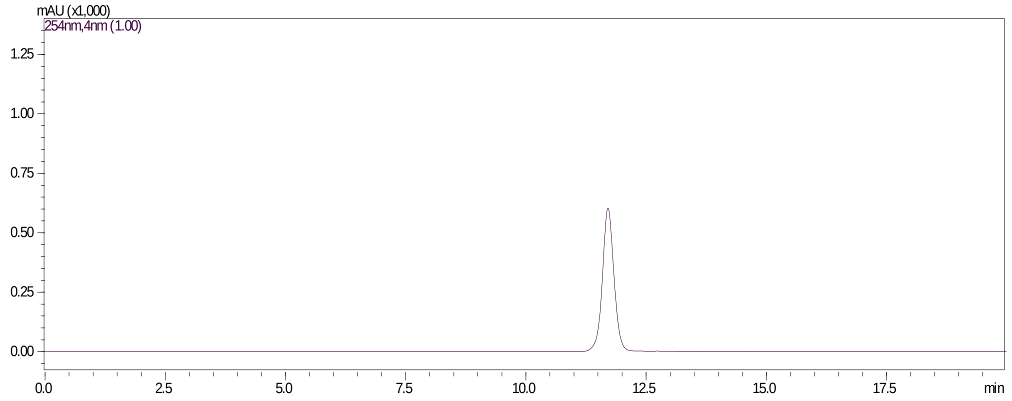

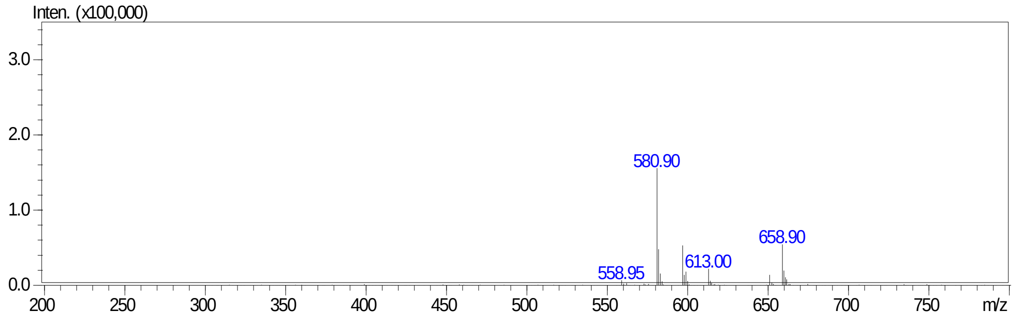

Figure S42. LC/ESI-MS analysis for 9d; ESI-MS, positive mode: *m/z* calcd mass for C24H26N6NaO6S2 $[M+Na]^+$ = 581.13, was found 580.90.

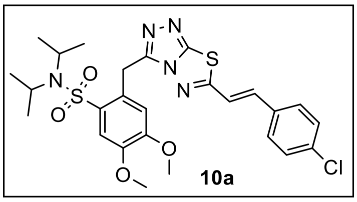

(a)

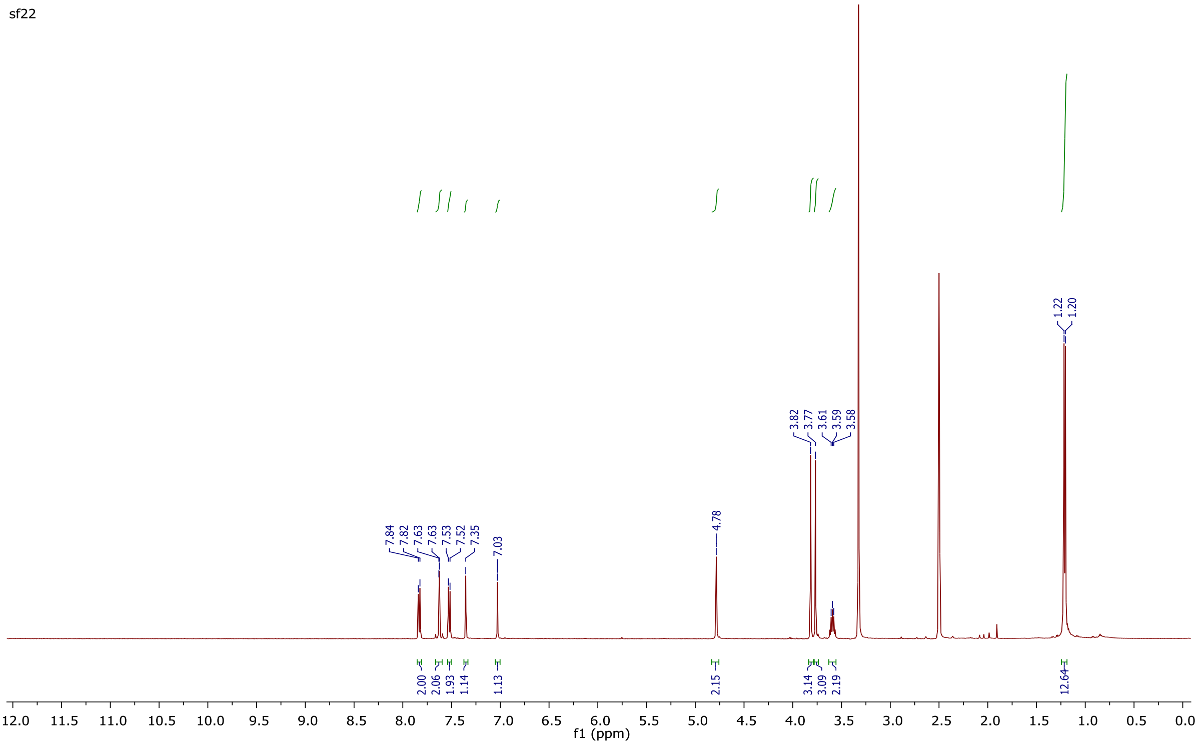

(b)

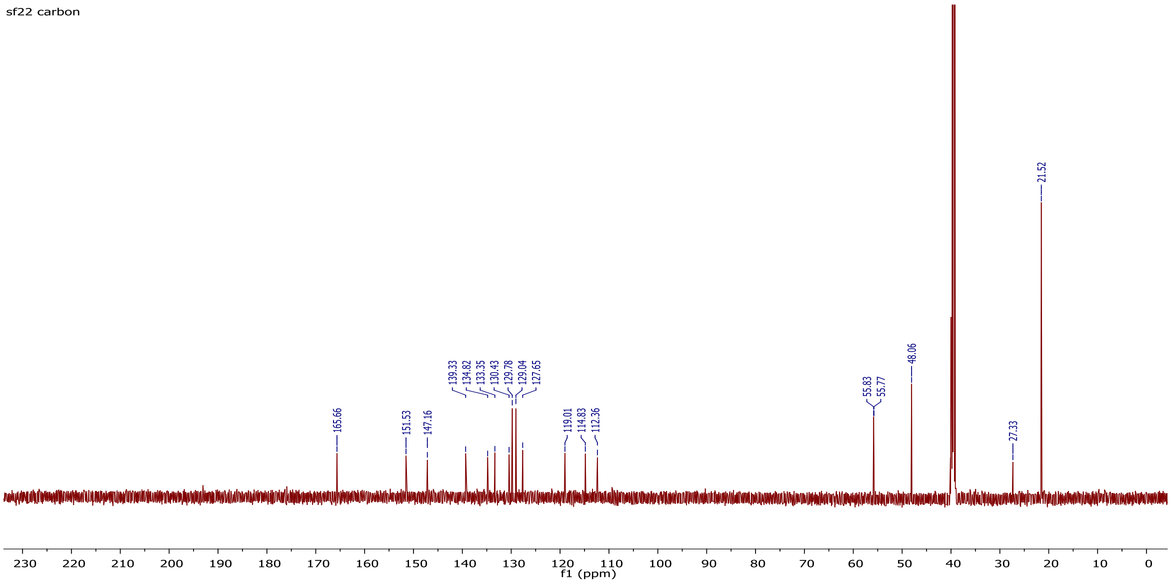

(c)

Figure S43. (**a**) 10a; **(b)** ^{1}H-NMR and (**c**) ^{13}C-NMR spectra for 10a.

Analytical method for LC-MS for 10a

LC-20AD Shimadzu connected to Shimadzu LCMS-2010EV
Mobile Phase: methanol
LC isocratic
HPLC column: SUPELCO Discovery C18, 25cm × 4.6mm, 5μm
Flow rate: 0.4 mL/min
Column temperature: 26 °C
UV detector: 254 nm
MS detector: 1.65 K
Run time: 10 min
Retention time: 8.7 min

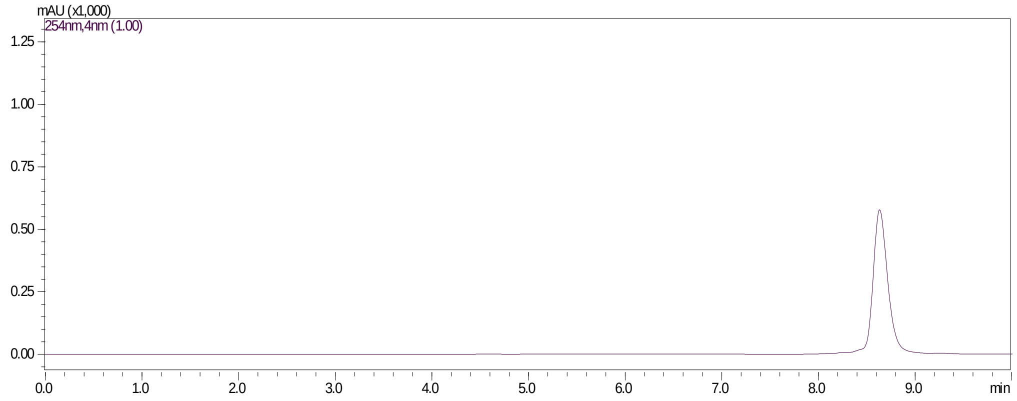

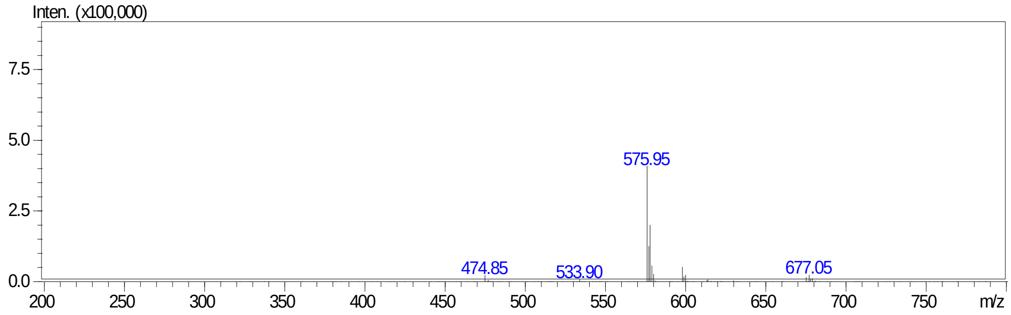

Figure S44. LC/ESI-MS analysis for 10a; ESI-MS, positive mode: *m*/*z* calcd mass for C26H31ClN5O4S2 $[M+H]^+$ = 576.15, was found 575.95.

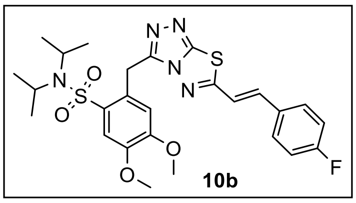

(a)

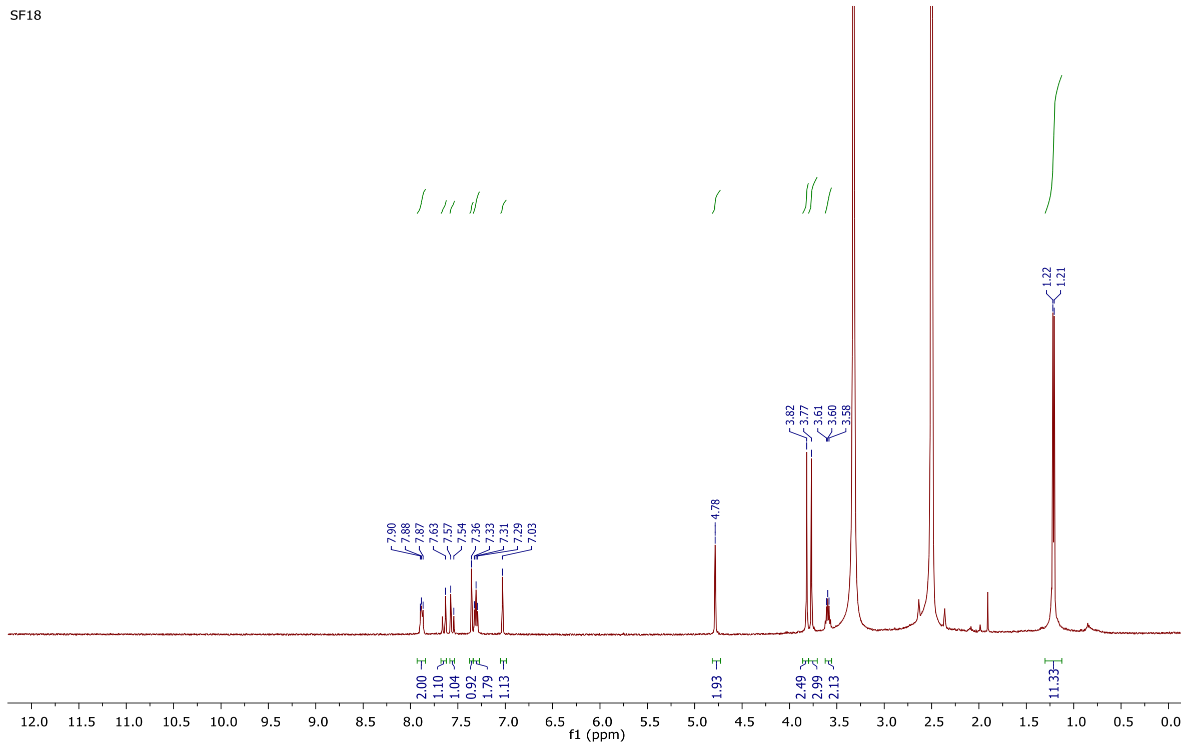

(b)

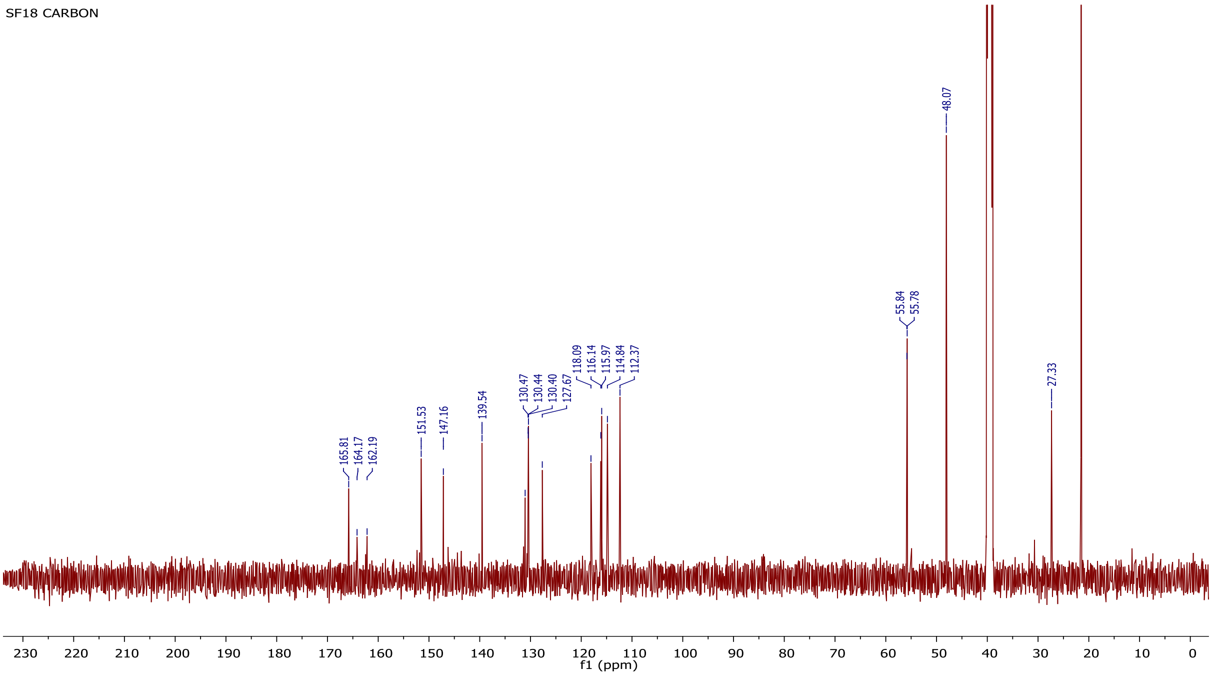

(c)

Figure S45. (**a**) 10b; **(b)** ^{1}H-NMR and (**c**) ^{13}C-NMR spectra for 10b.

Analytical method for LC-MS for 10b

LC-20AD Shimadzu connected to Shimadzu LCMS-2010EV
Mobile Phase: methanol
LC isocratic
HPLC column: SUPELCO Discovery C18, 25cm × 4.6mm, 5µm
Flow rate: 0.4 mL/min
Column temperature: 26 °C
UV detector: 254 nm
MS detector: 1.65 K
Run time: 20 min
Retention time: 11.4 min

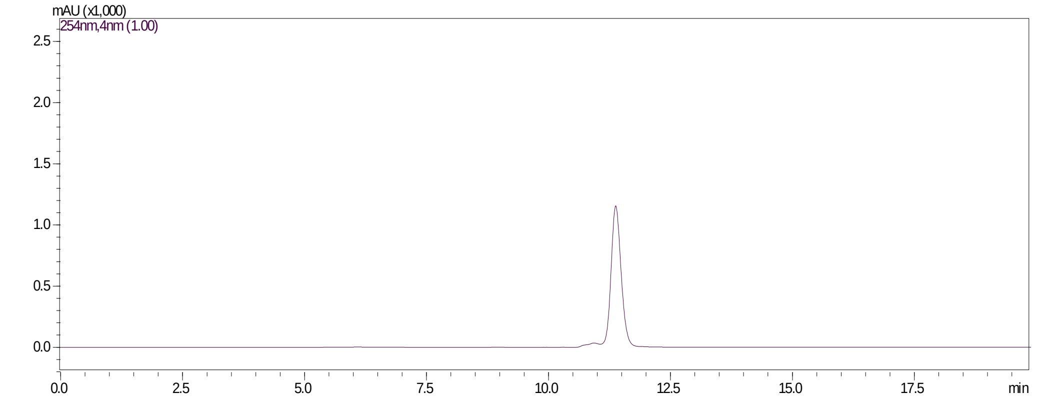

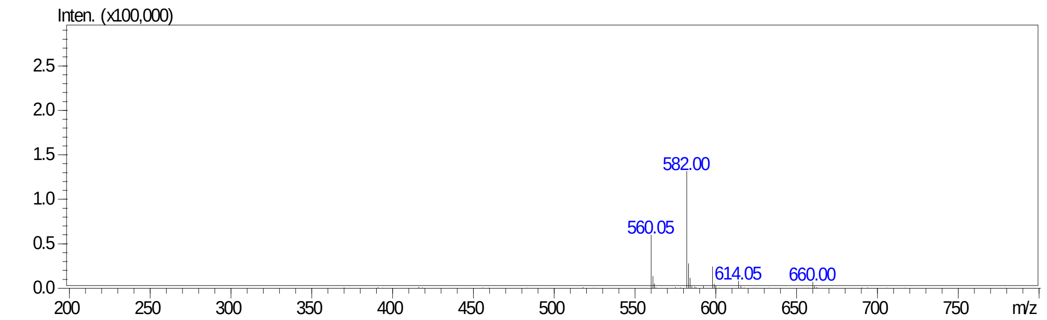

Figure S46. LC/ESI-MS analysis for 10b; ESI-MS, positive mode: *m/z* calcd mass for C26H31FN5O4S2 $[M+H]^+$ = 560.18, was found 560.05.

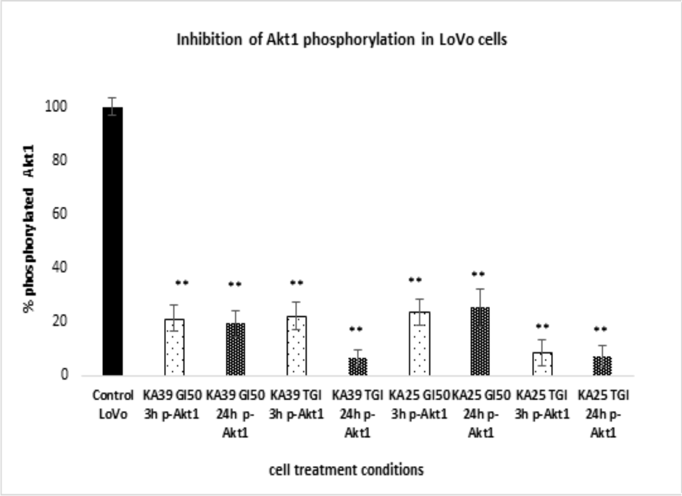

(a)

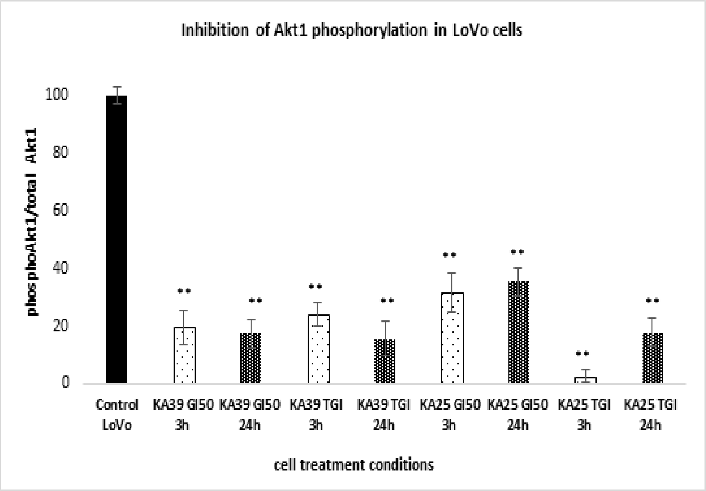

(b)

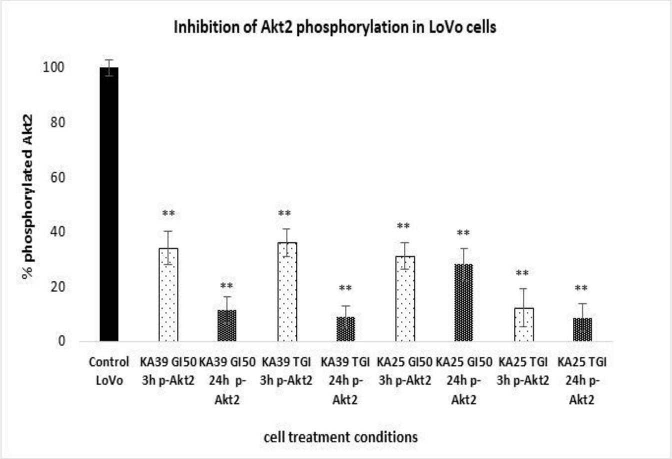

(c)

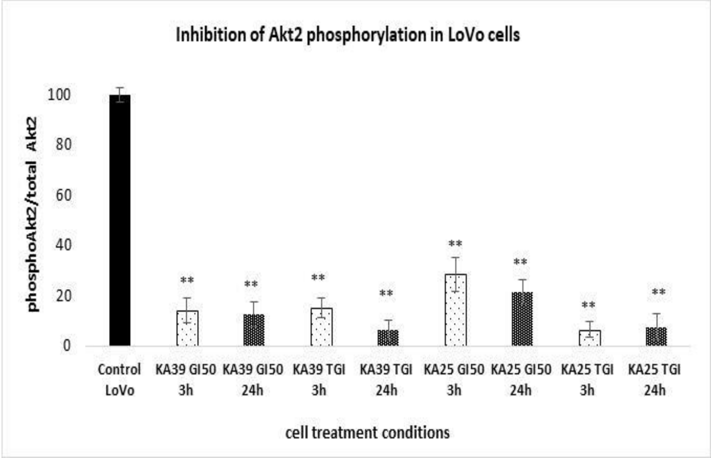

(d)

Figure S47 Inhibition of Akt1 and Akt2 phosphorylation in LoVo cells treated with the triazolo[3,4-*b*]thiadiazole derivatives KA25 and KA39 at GI_{50} and TGI concentrations for 3 and 24 h. **(a)** The diagram demonstrates the KA25- and KA39-induced inhibition of Akt1 phosphorylation expressed as absolute values (%). **(b)** The diagram shows the induced inhibition of Akt1 phosphorylation by KA25 and KA39 according to the ratios phosphoAkt1/total Akt1 correlated with control (untreated cells). **(c)** The diagram exhibits the KA25- and KA39-induced inhibition of Akt2 phosphorylation expressed as absolute values (%). **(d)** The diagram illustrates the induced inhibition of Akt2 phosphorylation by KA25 and KA39 according to the ratios phosphoAkt2/total Akt2 correlated with control (untreated cells). * $p < 0.05$, ** $p < 0.01$, *** $p < 0.001$.

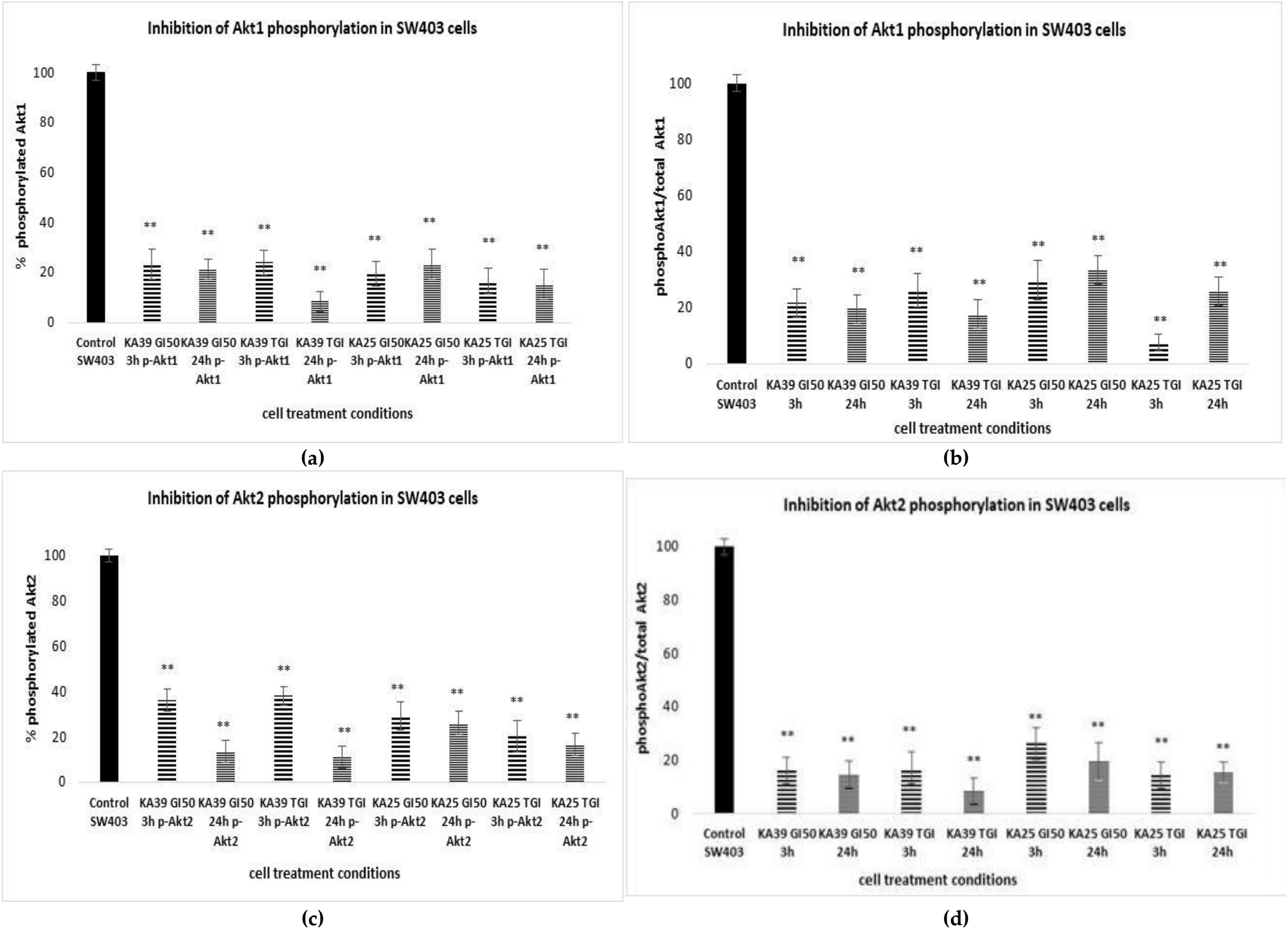

Figure S48. Inhibition of Akt1 and Akt2 phosphorylation in SW403 cells treated with the triazolo[3,4-*b*]thiadiazole derivatives KA25 and KA39 at GI_{50} and TGI concentrations for 3 and 24 h. **(a)** The diagram demonstrates the KA25- and KA39-induced inhibition of Akt1 phosphorylation expressed as absolute values (%). **(b)** The diagram displays the induced inhibition of Akt1 phosphorylation by KA25 and KA39 according to the ratios phosphoAkt1/total Akt1 correlated with control (untreated cells). **(c)** The diagram illustrates the KA25- and KA39-induced inhibition of Akt2 phosphorylation expressed as absolute values (%). **(d)** The diagram shows the induced inhibition of Akt2 phosphorylation by KA25 and KA39 according to the ratios phosphoAkt2/total Akt2 correlated with control (untreated cells). * $p < 0.05$, ** $p < 0.01$, *** $p < 0.001$.

Flow Cytometric Analysis Data

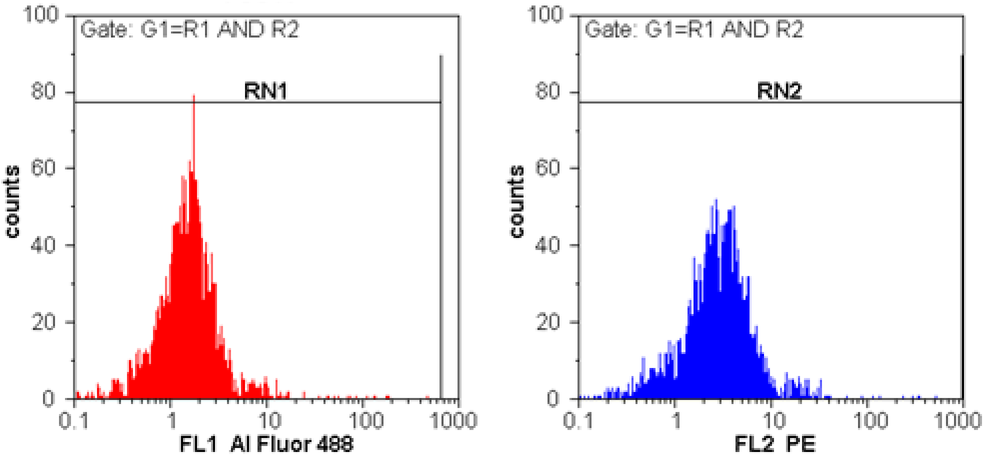

Figure S49. Flow cytometric analysis of Akt1 expression on untreated HT-29 cells. (**A**) RN1 (red peaks) and RN2 (blue peaks) regions display the expression of total Akt2 and phosphoAkt2, respectively.

Table S24. Region Analysis–calculations as resulted from the CyFlow® Partec Report software.

Region	Gate	Ungated	Count	Count/mL	%Gated	Mean-x	CV-x%	Mean-y	CV-y%
R1	<None>	2695	2695	-	51.39	106.54	52.27	78.99	66.85
R2	<None>	2226	2226	-	42.45	82.57	72.68	2.18	12.30
RN1	G1	5244	2155	-	100.00	2.72	488.06	-	-
RN2	G1	5244	2155	-	100.00	4.87	382.07	-	-

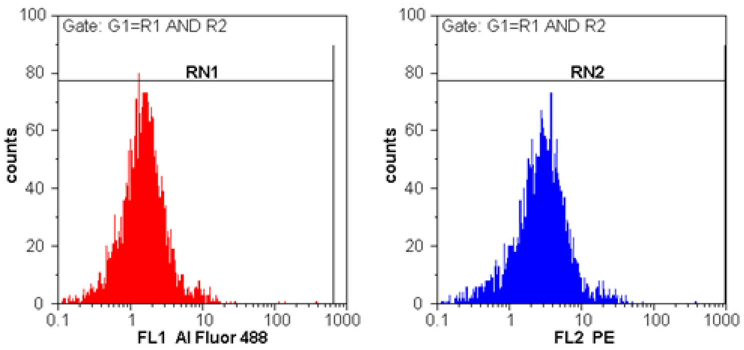

Figure S50. Flow cytometric analysis of Akt2 expression on untreated HT-29 cells. (**A**) RN1 region (red peaks) corresponds to total Akt1 expression and RN2 region (blue peaks) represents phosphoAkt1 expression.

Table S25. Region Analysis–calculations as resulted from the CyFlow® Partec Report software.

Region	Gate	Ungated	Count	Count/mL	%Gated	Mean-x	CV-x%	Mean-y	CV-y%
R1	<None>	3482	3482	-	54.47	106.64	51.79	78.64	66.90
R2	<None>	3004	3004	-	46.99	82.89	74.26	2.19	13.02
RN1	G1	6392	2871	-	99.97	2.20	367.85	-	-
RN2	G1	6393	2872	-	100.00	4.12	206.16	-	-

(A)

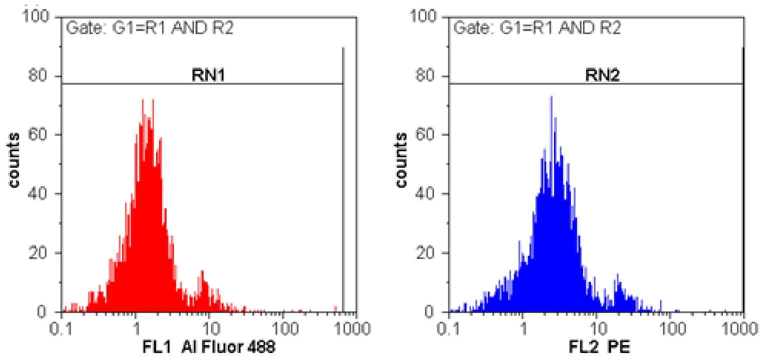

(B) Region Analysis.

Region	Gate	Ungated	Count	Count/mL	%Gated	Mean-x	CV-x%	Mean-y	CV-y%
R1	<None>	3255	3255	-	59.34	116.70	49.76	90.04	63.36
R2	<None>	2758	2758	-	50.28	94.06	70.72	2.22	12.94
RN1	G1	5485	2674	-	100.00	3.06	545.07	-	-
RN2	G1	5485	2674	-	100.00	5.08	297.99	-	-

Figure S51. Flow cytometric analysis of Akt1 expression on HT-29 cells treated with KA39 at GI_{50} concentration (μM) for 3h. (**A**) RN1 (red peaks) and RN2 (blue peaks) regions display the expression of total Akt2 and phosphoAkt2, respectively. (**B**) The values included in the table are the calculations as resulted from the CyFlow® Partec Report software.

(A)

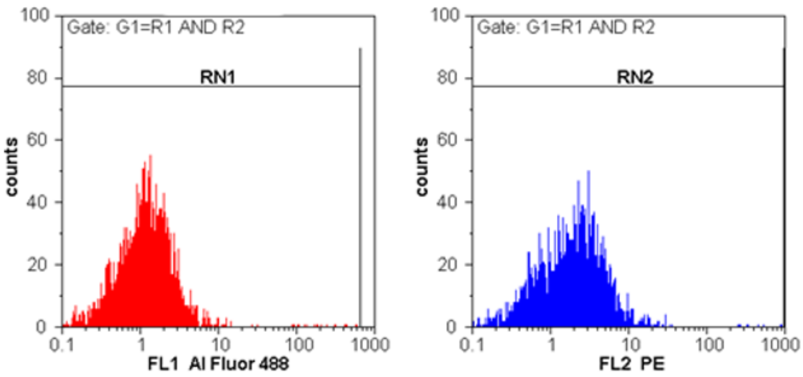

(B) Region Analysis.

Region	Gate	Ungated	Count	Count/mL	%Gated	Mean-x	CV-x%	Mean-y	CV-y%
R1	<None>	2783	2783	-	53.23	105.75	53.36	88.66	64.92
R2	<None>	2379	2379	-	45.50	81.45	77.18	2.17	12.63
RN1	G1	5227	2270	-	100.00	3.12	724.29	-	-
RN2	G1	5228	2270	-	100.00	4.61	738.30	-	-

Figure S52. Flow cytometric analysis of Akt1 expression on HT-29 cells treated with KA39 at GI_{50} concentration (μM) for 24h. (**A**) RN1 region (red peaks) corresponds to total Akt1 expression and RN2 region (blue peaks) represents phosphoAkt1 expression. (**B**) The values included in the table are the calculations as resulted from the CyFlow® Partec Report software.

(A)

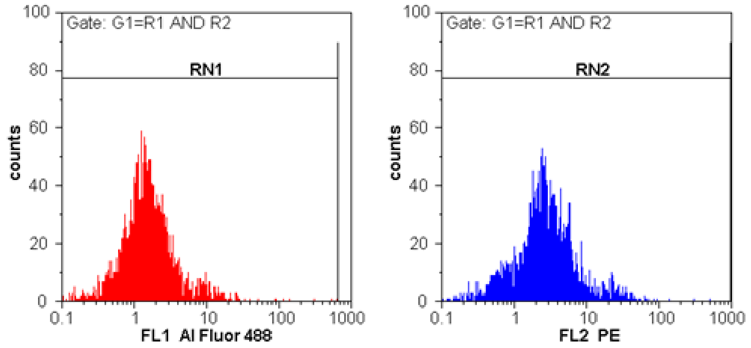

(B) Region Analysis.

Region	Gate	Ungated	Count	Count/mL	%Gated	Mean-x	CV-x%	Mean-y	CV-y%
R1	<None>	2807	2807	-	52.46	122.90	50.60	92.18	65.23
R2	<None>	2293	2293	-	42.85	100.06	72.07	2.24	13.32
RN1	G1	5350	2195	-	100.00	3.23	609.86	-	-
RN2	G1	5351	2195	-	100.00	5.22	287.72	-	-

Figure S53. Flow cytometric analysis of Akt1 expression on HT-29 cells treated with KA39 at TGI concentration (μM) for 3h. (**A**) RN1 (red peaks) and RN2 (blue peaks) regions display the expression of total Akt2 and phosphoAkt2, respectively. (**B**) The values included in the table are the calculations as resulted from the CyFlow® Partec Report software.

(A)

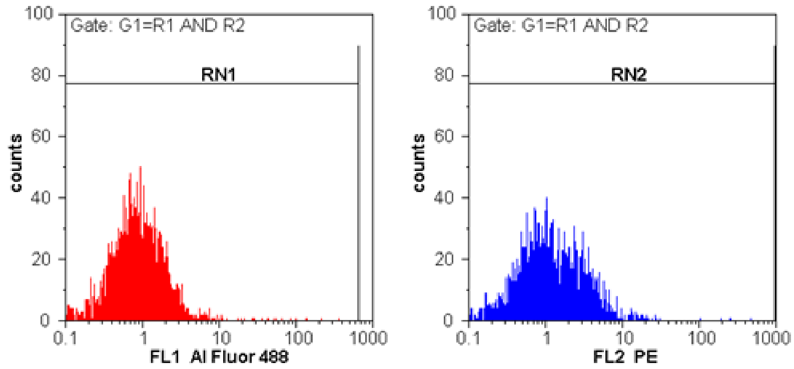

(B) Region Analysis.

Region	Gate	Ungated	Count	Count/mL	%Gated	Mean-x	CV-x%	Mean-y	CV-y%
R1	<None>	2494	2494	-	48.28	91.60	56.22	89.70	62.48
R2	<None>	2150	2150	-	41.62	64.67	86.91	2.11	11.88
RN1	G1	5166	2042	-	100.00	1.87	596.79	-	-
RN2	G1	5166	2042	-	100.00	2.50	529.38	-	-

Figure S54. Flow cytometric analysis of Akt1 expression on HT-29 cells treated with KA39 at TGI concentration (μM) for 24h. (**A**) RN1 region (red peaks) corresponds to total Akt1 expression and RN2 region (blue peaks) represents phosphoAkt1 expression. (**B**) The values included in the table are the calculations as resulted from the CyFlow® Partec Report software.

(A)

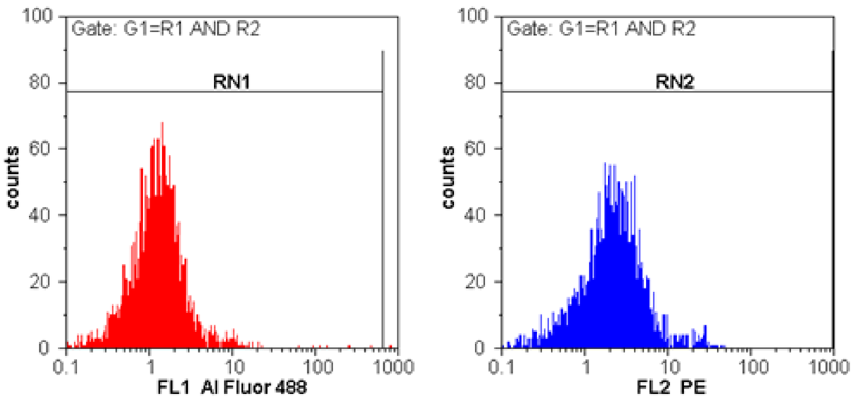

(B) Region Analysis.

Region	Gate	Ungated	Count	Count/mL	%Gated	Mean-x	CV-x%	Mean-y	CV-y%
R1	<None>	3059	3059	-	57.90	121.07	51.95	91.49	63.35
R2	<None>	2535	2535	-	47.98	97.09	75.36	2.22	13.08
RN1	G1	5281	2449	-	99.92	2.16	549.38	-	-
RN2	G1	5281	2449	-	99.92	3.21	133.54	-	-

Figure S55. Flow cytometric analysis of Akt1 expression on HT-29 cells treated with KA25 at GI_{50} concentration (µM) for 3h. (**A**) RN1 (red peaks) and RN2 (blue peaks) regions display the expression of total Akt1 and phosphoAkt1, respectively. (**B**) The values included in the table are the calculations as resulted from the CyFlow® Partec Report software.

(A)

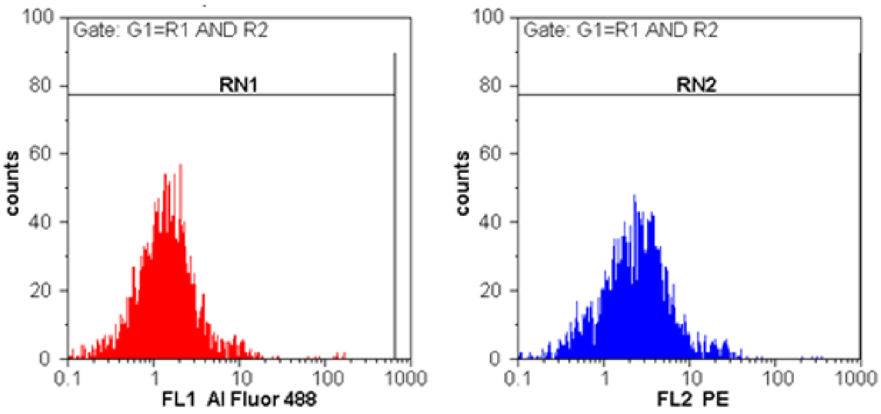

(B) Region Analysis.

Region	Gate	Ungated	Count	Count/mL	%Gated	Mean-x	CV-x%	Mean-y	CV-y%
R1	<None>	2914	2914	-	55.53	121.45	54.36	90.66	67.90
R2	<None>	2342	2342	-	44.63	98.80	76.75	2.24	13.72
RN1	G1	5247	2261	-	100.00	2.50	359.31	-	-
RN2	G1	5248	2261	-	100.00	4.27	309.75	-	-

Figure S56. Flow cytometric analysis of Akt1 expression on HT-29 cells treated with KA25 at GI_{50} concentration (µM) for 24h. (**A**) RN1 region (red peaks) corresponds to total Akt1 expression and RN2 region (blue peaks) represents phosphoAkt1 expression. (**B**) The values included in the table are the calculations as resulted from the CyFlow® Partec Report software.

(A)

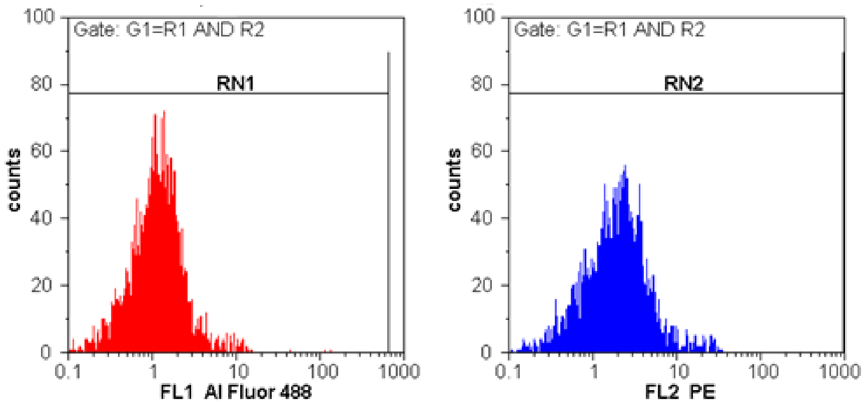

(B) Region Analysis.

Region	Gate	Ungated	Count	Count/mL	%Gated	Mean-x	CV-x%	Mean-y	CV-y%
R1	<None>	3119	3119	-	60.87	110.90	51.10	87.76	65.49
R2	<None>	2682	2682	-	52.34	84.76	74.95	2.18	12.26
RN1	G1	5124	2589	-	100.00	1.62	243.24	-	-
RN2	G1	5124	2589	-	100.00	2.84	130.26	-	-

Figure S57. Flow cytometric analysis of Akt1 expression on HT-29 cells treated with KA25 at TGI concentration (µM) for 3h. (**A**) RN1 (red peaks) and RN2 (blue peaks) regions display the expression of total Akt1 and phosphoAkt1, respectively. (**B**) The values included in the table are the calculations as resulted from the CyFlow® Partec Report software.

(A)

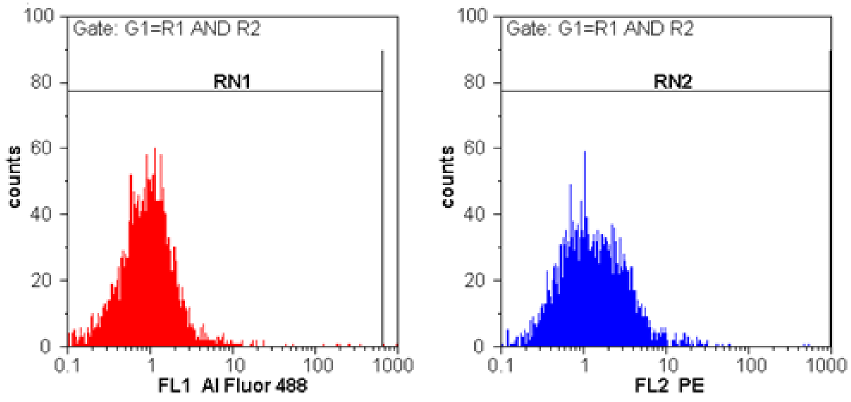

(B) Region Analysis.

Region	Gate	Ungated	Count	Count/mL	%Gated	Mean-x	CV-x%	Mean-y	CV-y%
R1	<None>	3030	3030	-	54.16	98.23	56.66	83.50	64.36
R2	<None>	2530	2530	-	45.22	72.94	81.92	2.16	12.89
RN1	G1	5593	2416	-	99.92	1.77	619.59	-	-
RN2	G1	5594	2418	-	100.00	2.53	597.45	-	-

Figure S58. Flow cytometric analysis of Akt1 expression on HT-29 cells treated with KA25 at TGI concentration (µM) for 24h. (**A**) RN1 region (red peaks) corresponds to total Akt1 expression and RN2 region (blue peaks) represents phosphoAkt1 expression. (**B**) The values included in the table are the calculations as resulted from the CyFlow® Partec Report software.

(A)

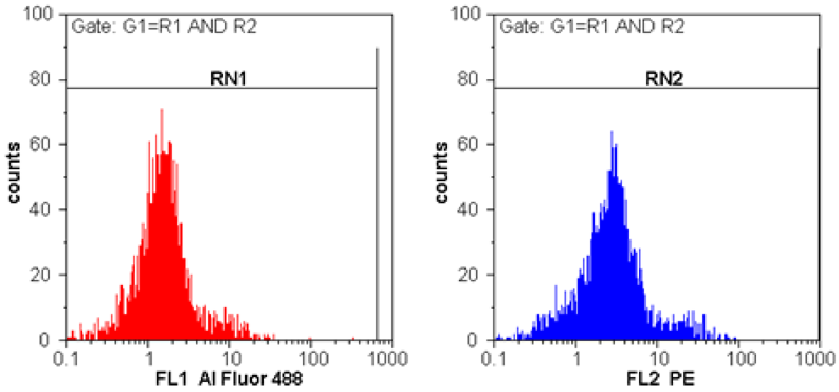

(B) Region Analysis.

Region	Gate	Ungated	Count	Count/mL	%Gated	Mean-x	CV-x%	Mean-y	CV-y%
R1	<None>	2996	2996	-	56.88	117.54	49.29	90.95	62.47
R2	<None>	2558	2558	-	48.57	92.78	72.29	2.22	12.80
RN1	G1	5267	2467	-	100.00	2.57	296.00	-	-
RN2	G1	5267	2467	-	100.00	4.95	166.04	-	-

Figure S59. Flow cytometric analysis of Akt2 expression on HT-29 cells treated with KA39 at GI$_{50}$ concentration (μM) for 3h. (**A**) RN1 (red peaks) and RN2 (blue peaks) regions display the expression of total Akt2 and phosphoAkt2, respectively. (**B**) The values included in the table are the calculations as resulted from the CyFlow® Partec Report software.

(A)

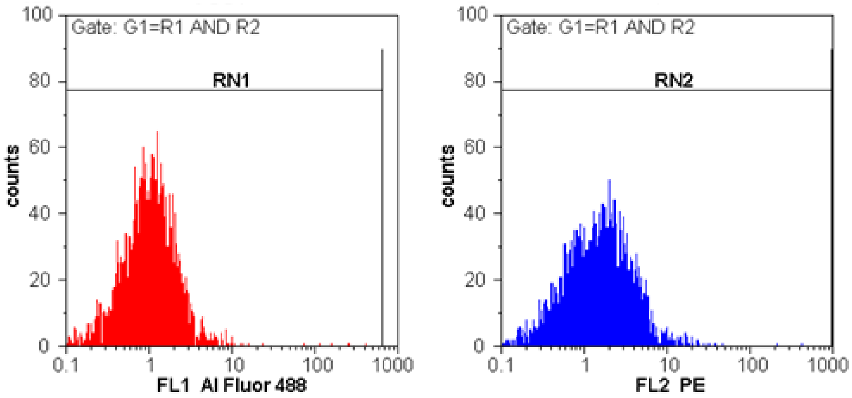

(B) Region Analysis.

Region	Gate	Ungated	Count	Count/mL	%Gated	Mean-x	CV-x%	Mean-y	CV-y%
R1	<None>	3303	3303	-	47.61	102.63	56.92	91.17	70.66
R2	<None>	2677	2677	-	38.59	80.79	76.57	2.18	12.69
RN1	G1	6937	2570	-	100.00	1.71	621.46	-	-
RN2	G1	6937	2570	-	100.00	2.52	395.68	-	-

Figure S60. Flow cytometric analysis of Akt2 expression on HT-29 cells treated with KA39 at GI$_{50}$ concentration (μM) for 24h. (**A**) RN1 region (red peaks) corresponds to total Akt2 expression and RN2 region (blue peaks) represents phosphoAkt2 expression. (**B**) The values included in the table are the calculations as resulted from the CyFlow® Partec Report software.

(A)

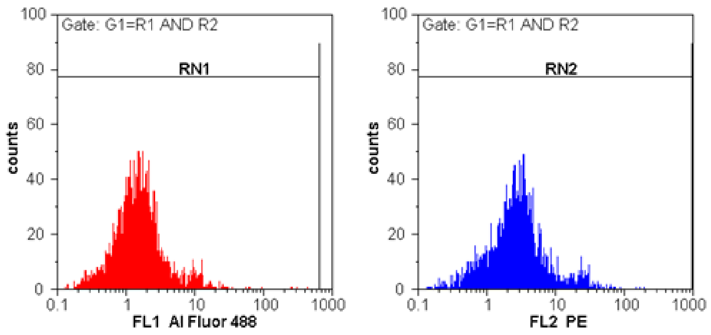

(B) Region Analysis.

Region	Gate	Ungated	Count	Count/mL	%Gated	Mean-x	CV-x%	Mean-y	CV-y%
R1	<None>	2679	2679	-	51.26	122.69	50.74	91.95	64.39
R2	<None>	2139	2139	-	40.93	98.80	71.26	2.25	13.83
RN1	G1	5226	2052	-	100.00	3.02	463.64	-	-
RN2	G1	5226	2052	-	100.00	5.18	195.48	-	-

Figure S61. Flow cytometric analysis of Akt2 expression on HT-29 cells treated with KA39 at TGI concentration (μM) for 3h. (**A**) RN1 (red peaks) and RN2 (blue peaks) regions display the expression of total Akt2 and phosphoAkt2, respectively. (**B**) The values included in the table are the calculations as resulted from the CyFlow® Partec Report software.

(A)

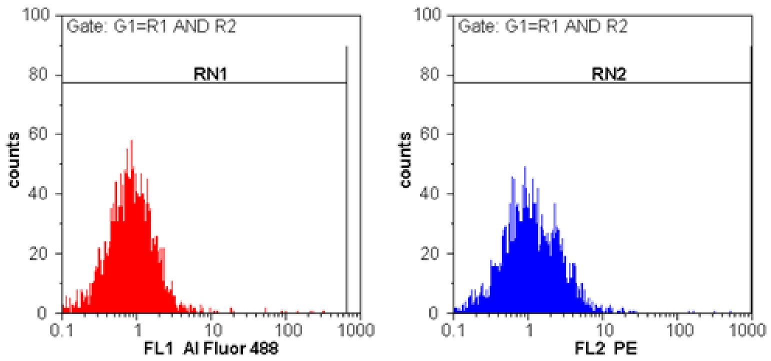

(B) Region Analysis.

Region	Gate	Ungated	Count	Count/mL	%Gated	Mean-x	CV-x%	Mean-y	CV-y%
R1	<None>	2756	2756	-	52.78	92.11	56.69	91.89	64.11
R2	<None>	2357	2357	-	45.14	66.07	88.09	2.12	12.36
RN1	G1	5220	2277	-	100.00	1.75	719.01	-	-
RN2	G1	5221	2277	-	100.00	2.12	657.39	-	-

Figure S62. Flow cytometric analysis of Akt2 expression on HT-29 cells treated with KA39 at TGI concentration (μM) for 24h. (**A**) RN1 region (red peaks) corresponds to total Akt2 expression and RN2 region (blue peaks) represents phosphoAkt2 expression. (**B**) The values included in the table are the calculations as resulted from the CyFlow® Partec Report software.

(A)

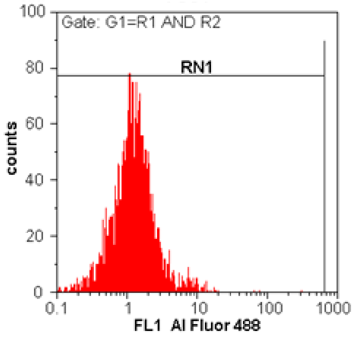

(B) Region Analysis.

Region	Gate	Ungated	Count	Count/ml	%Gated	Mean-x	CV-x%	Mean-y	CV-y%
R1	<None>	3346	3346	-	62.68	124.52	50.82	93.59	64.21
R2	<None>	2754	2754	-	51.59	102.76	72.79	2.24	13.45
RN1	G1	5338	2680	-	100.00	1.82	361.00	-	-
RN2	G1	5338	2680	-	100.00	3.28	200.85	-	-

Figure S63. Flow cytometric analysis of Akt2 expression on HT-29 cells treated with KA25 at GI_{50} concentration (μM) for 3h. (**A**) RN1 (red peaks) and RN2 (blue peaks) regions display the expression of total Akt2 and phosphoAkt2, respectively. (**B**) The values included in the table are the calculations as resulted from the CyFlow® Partec Report software.

(A)

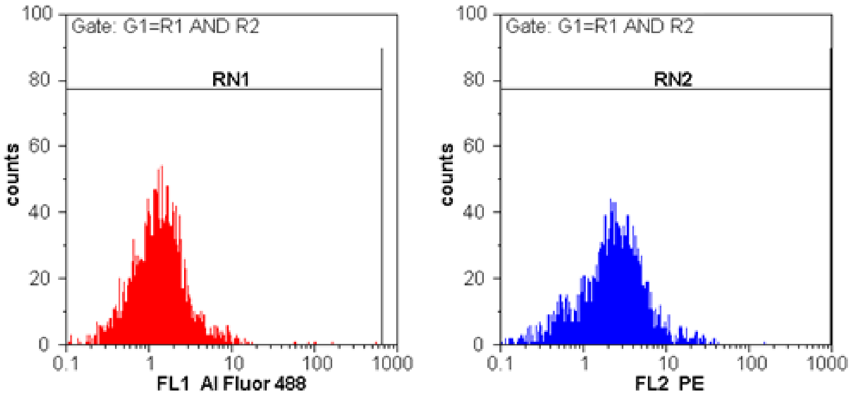

(B) Region Analysis.

Region	Gate	Ungated	Count	Count/ml	%Gated	Mean-x	CV-x%	Mean-y	CV-y%
R1	<None>	2612	2612	-	50.87	122.51	54.64	88.77	64.80
R2	<None>	2117	2117	-	41.23	99.80	77.27	2.24	13.70
RN1	G1	5135	2026	-	100.00	2.29	612.00	-	-
RN2	G1	5135	2026	-	100.00	3.46	154.02	-	-

Figure S64. Flow cytometric analysis of Akt2 expression on HT-29 cells treated with KA25 at GI_{50} concentration (μM) for 24h. (**A**) RN1 region (red peaks) corresponds to total Akt2 expression and RN2 region (blue peaks) represents phosphoAkt2 expression. (**B**) The values included in the table are the calculations as resulted from the CyFlow® Partec Report software.

(A)

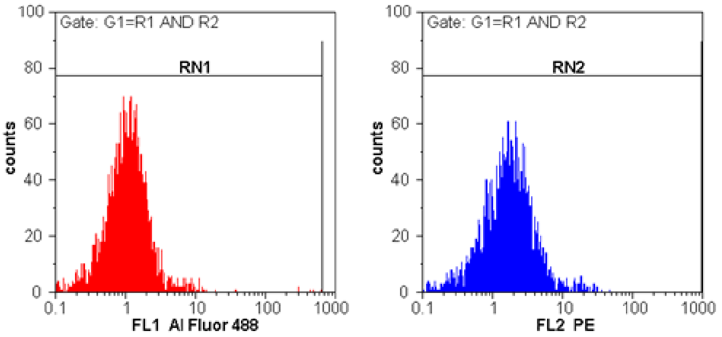

(B) Region Analysis.

Region	Gate	Ungated	Count	Count/ml	%Gated	Mean-x	CV-x%	Mean-y	CV-y%
R1	<None>	3289	3289	-	62.04	111.18	52.79	89.58	65.29
R2	<None>	2760	2760	-	52.07	85.37	75.93	2.19	12.81
RN1	G1	5301	2671	-	100.00	2.20	887.83	-	-
RN2	G1	5301	2671	-	100.00	2.51	133.89	-	-

Figure S65. Flow cytometric analysis of Akt2 expression on HT-29 cells treated with KA25 at TGI concentration (μM) for 3h. (**A**) RN1 (red peaks) and RN2 (blue peaks) regions display the expression of total Akt2 and phosphoAkt2, respectively. (**B**) The values included in the table are the calculations as resulted from the CyFlow® Partec Report software.

(A)

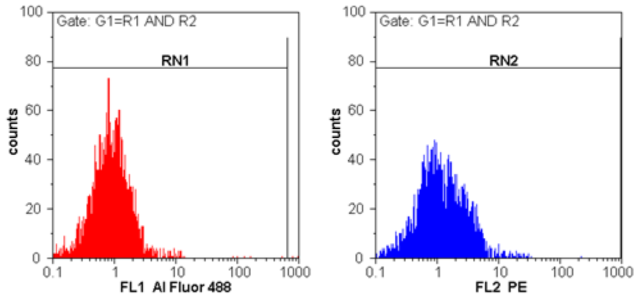

(B) Region Analysis.

Region	Gate	Ungated	Count	Count/ml	%Gated	Mean-x	CV-x%	Mean-y	CV-y%
R1	<None>	3068	3068	-	52.17	99.31	56.41	83.41	64.36
R2	<None>	2606	2606	-	44.31	73.59	82.47	2.16	12.49
RN1	G1	5879	2484	-	99.92	1.58	770.63	-	-
RN2	G1	5881	2486	-	100.00	2.01	260.05	-	-

Figure S66. Flow cytometric analysis of Akt2 expression on HT-29 cells treated with KA25 at TGI concentration (μM) for 24h. (**A**) RN1 region (red peaks) corresponds to total Akt2 expression and RN2 region (blue peaks) represents phosphoAkt2 expression. (**B**) The values included in the table are the calculations as resulted from the CyFlow® Partec Report software.

(A)

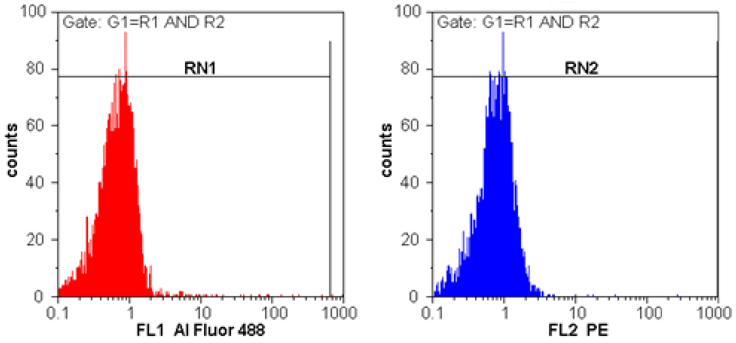

(B) Region Analysis.

Region	Gate	Ungated	Count	Count/ml	%Gated	Mean-x	CV-x%	Mean-y	CV-y%
R1	<None>	3628	3628	-	54.10	103.60	52.57	79.70	68.84
R2	<None>	3034	3034	-	45.24	79.91	73.18	2.18	12.69
RN1	G1	6704	2942	-	99.93	1.31	903.08	-	-
RN2	G1	6705	2944	-	100.00	0.96	538.13	-	-

Figure S67. Flow cytometric analysis of untreated HT-29 cells. (**A**) RN1 (red peaks) and RN2 (blue peaks) regions concern the isotype control of total Akt1/2 and phosphoAkt1/2, respectively. (**B**) The values included in the table are the calculations as resulted from the CyFlow® Partec Report software.

(A)

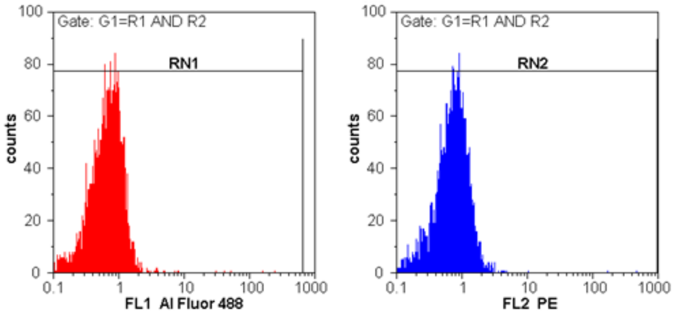

(B) Region Analysis.

Region	Gate	Ungated	Count	Count/ml	%Gated	Mean-x	CV-x%	Mean-y	CV-y%
R1	<None>	3316	3316	-	61.14	114.72	50.03	89.89	64.43
R2	<None>	2819	2819	-	51.97	90.88	73.63	2.20	12.76
RN1	G1	5424	2756	-	100.00	0.92	637.14	-	-
RN2	G1	5424	2756	-	100.00	1.02	933.00	-	-

Figure S68. Flow cytometric analysis of HT-29 cells treated with KA39 at GI_{50} concentration (μM) for 3h. (**A**) RN1 (red peaks) and RN2 (blue peaks) regions concern the isotype control of total Akt1/2 and phosphoAkt1/2, respectively. (**B**) The values included in the table are the calculations as resulted from the CyFlow® Partec Report software.

(A)

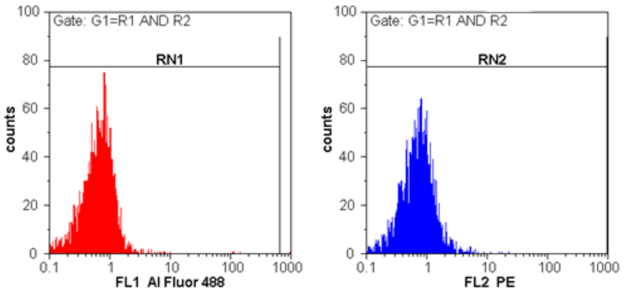

(B) Region Analysis.

Region	Gate	Ungated	Count	Count/ml	%Gated	Mean-x	CV-x%	Mean-y	CV-y%
R1	<None>	2699	2699	-	49.67	104.52	52.70	87.32	64.15
R2	<None>	2297	2297	-	42.27	78.20	77.20	2.17	12.12
RN1	G1	5433	2181	-	99.95	0.96	543.69	-	-
RN2	G1	5434	2182	-	100.00	0.86	114.50	-	-

Figure S69. Flow cytometric analysis of HT-29 cells treated with KA39 GI_{50} concentration (μM) for 24h. (**A**) RN1 (red peaks) and RN2 (blue peaks) regions concern the isotype control of total Akt1/2 and phosphoAkt1/2, respectively. (**B**) The values included in the table are the calculations as resulted from the CyFlow® Partec Report software.

(A)

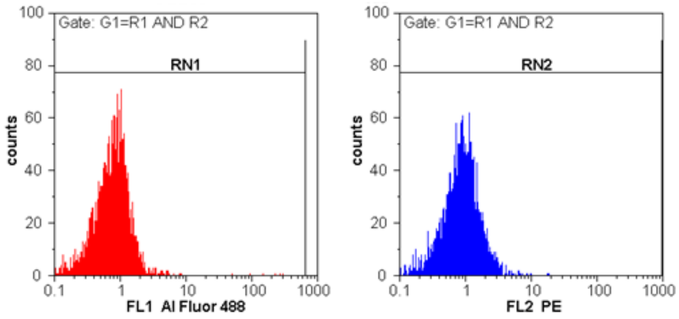

(B) Region Analysis.

Region	Gate	Ungated	Count	Count/ml	%Gated	Mean-x	CV-x%	Mean-y	CV-y%
R1	<None>	2713	2713	-	52.07	120.87	51.89	90.76	64.17
R2	<None>	2233	2233	-	42.86	98.06	73.66	2.23	13.33
RN1	G1	5210	2132	-	100.00	1.39	778.12	-	-
RN2	G1	5210	2132	-	100.00	1.07	93.93	-	-

Figure S70. Flow cytometric analysis of HT-29 cells treated with KA39 at TGI concentration (μM) for 3h. (**A**) RN1 (red peaks) and RN2 (blue peaks) regions concern the isotype control of total Akt1/2 and phosphoAkt1/2, respectively. (**B**) The values included in the table are the calculations as resulted from the CyFlow® Partec Report software.

(A)

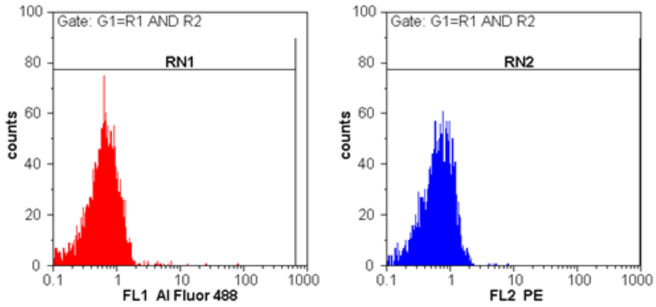

(B) Region Analysis.

Region	Gate	Ungated	Count	Count/ml	%Gated	Mean-x	CV-x%	Mean-y	CV-y%
R1	<None>	2519	2519	-	48.61	93.43	57.11	91.42	63.55
R2	<None>	2138	2138	-	41.26	68.66	86.07	2.12	12.08
RN1	G1	5182	2065	-	100.00	0.75	259.59	-	-
RN2	G1	5182	2065	-	100.00	0.71	63.85	-	-

Figure S71. Flow cytometric analysis of HT-29 cells treated with KA39 at TGI concentration (μM) for 24h. (**A**) RN1 (red peaks) and RN2 (blue peaks) regions concern the isotype control of total Akt1/2 and phosphoAkt1/2, respectively. (**B**) The values included in the table are the calculations as resulted from the CyFlow® Partec Report software.

(A)

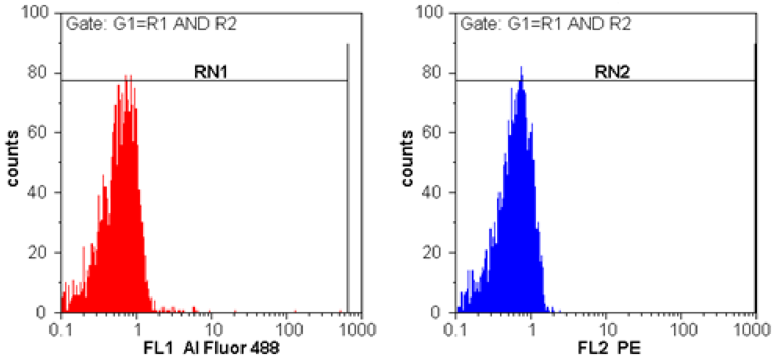

(B) Region Analysis.

Region	Gate	Ungated	Count	Count/ml	%Gated	Mean-x	CV-x%	Mean-y	CV-y%
R1	<None>	3239	3239	-	56.90	121.16	51.87	90.71	63.55
R2	<None>	2756	2756	-	48.42	97.40	74.13	2.22	13.33
RN1	G1	5692	2668	-	100.00	0.91	1155.35	-	-
RN2	G1	5692	2668	-	100.00	0.64	51.20	-	-

Figure S72. Flow cytometric analysis of HT-29 cells treated with KA25 at GI_{50} concentration (μM) for 3h. (**A**) RN1 (red peaks) and RN2 (blue peaks) regions concern the isotype control of total Akt1/2 and phosphoAkt1/2, respectively. (**B**) The values included in the table are the calculations as resulted from the CyFlow® Partec Report software.

(A)

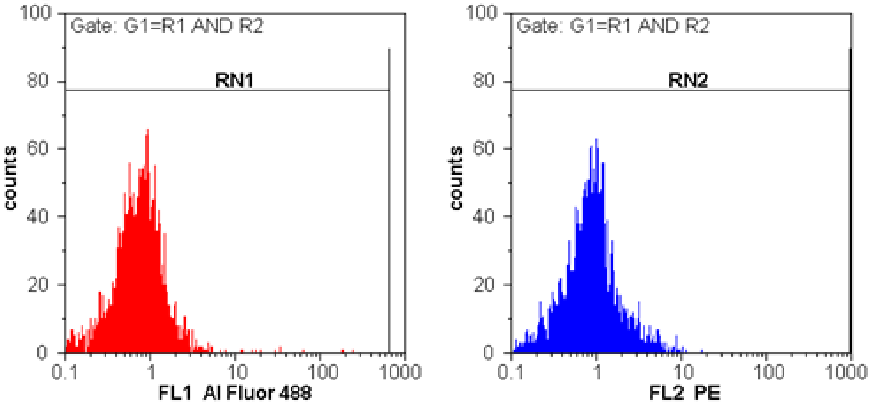

(B) Region Analysis.

Region	Gate	Ungated	Count	Count/ml	%Gated	Mean-x	CV-x%	Mean-y	CV-y%
R1	<None>	2788	2788	-	53.19	122.42	54.28	89.55	65.08
R2	<None>	2295	2295	-	43.78	101.36	77.12	2.23	13.35
RN1	G1	5242	2219	-	100.00	1.17	584.62	-	-
RN2	G1	5242	2219	-	100.00	1.15	106.20	-	-

Figure S73. Flow cytometric analysis of HT-29 cells treated with KA25 at GI_{50} concentration (μM) for 24h. (**A**) RN1 (red peaks) and RN2 (blue peaks) regions concern the isotype control of total Akt1/2 and phosphoAkt1/2, respectively. (**B**) The values included in the table are the calculations as resulted from the CyFlow® Partec Report software.

(A)

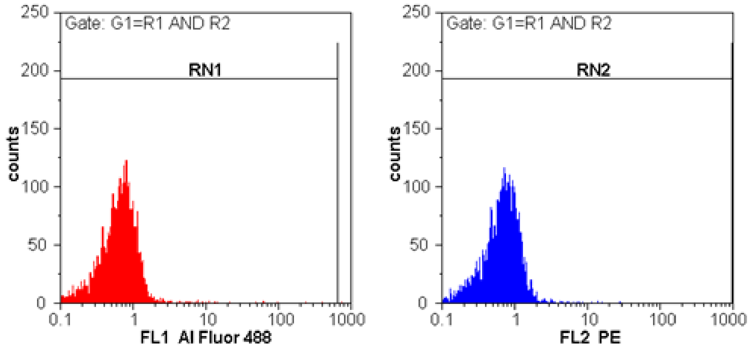

(B) Region Analysis.

Region	Gate	Ungated	Count	Count/ml	%Gated	Mean-x	CV-x%	Mean-y	CV-y%
R1	<None>	4680	4680	-	53.63	108.32	55.25	91.82	68.65
R2	<None>	3910	3910	-	44.80	87.75	75.45	2.19	12.56
RN1	G1	8726	3783	-	99.97	0.99	811.97	-	-
RN2	G1	8727	3784	-	100.00	0.76	108.03	-	-

Figure S74. Flow cytometric analysis of HT-29 cells treated with KA25 at TGI concentration (μM) for 3h.(**A**) RN1 (red peaks) and RN2 (blue peaks) regions concern the isotype control of total Akt1/2 and phosphoAkt1/2, respectively. (**B**) The values included in the table are the calculations as resulted from the CyFlow® Partec Report software.

(A)

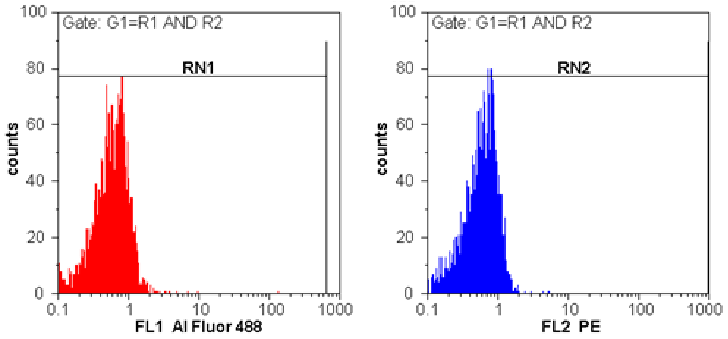

(B) Region Analysis.

Region	Gate	Ungated	Count	Count/ml	%Gated	Mean-x	CV-x%	Mean-y	CV-y%
R1	<None>	2866	2866	-	54.54	100.56	56.68	84.92	64.78
R2	<None>	2478	2478	-	47.16	76.50	83.02	2.15	11.87
RN1	G1	5255	2383	-	100.00	0.69	401.95	-	-
RN2	G1	5255	2383	-	100.00	0.62	56.97	-	-

Figure S75. Flow cytometric analysis of HT-29 cells treated with KA25 at TGI concentration (μM) for 24h. (**A**) RN1 (red peaks) and RN2 (blue peaks) regions concern the isotype control of total Akt1/2 and phosphoAkt1/2, respectively. (**B**) The values included in the table are the calculations as resulted from the CyFlow® Partec Report software.
